# Radiotherapy Fraction in Limited-Stage Small Cell Lung Cancer in the Modern Era: A Systematic Review and Meta-Analysis of 8006 Reconstructed Individual Patient Data

**DOI:** 10.3390/cancers15010277

**Published:** 2022-12-31

**Authors:** Jingjing Zhao, Linfang Wu, Chen Hu, Nan Bi, Luhua Wang

**Affiliations:** 1 Department of Radiation Oncology, National Cancer Center/National Clinical Research Center for Cancer/Cancer Hospital, Chinese Academy of Medical Sciences and Peking Union Medical College, Beijing 100021, China; 2 Cancer Research UK Cambridge Institute, University of Cambridge Li Ka Shing Center, Cambridge CB2 0RE, UK; 3 Division of Quantitative Sciences, Sidney Kimmel Comprehensive Cancer Center, Johns Hopkins University School of Medicine, Baltimore, MD 21205, USA; 4 Department of Radiation Oncology, National Cancer Center/National Clinical Research Center for Cancer/Cancer Hospital & Shenzhen Hospital, Chinese Academy of Medical Sciences and Peking Union Medical College, Shenzhen 518116, China

**Keywords:** small cell lung cancer, radiation therapy, dose fractionation, systematic review, meta-analysis

## Abstract

**Simple Summary:**

The optimal thoracic radiotherapy (TRT) dose and fractionation for limited-stage small cell lung cancer (LS-SCLC) remains debatable due to inconclusive evidence. With a comprehensive systematic review involving not only randomized controlled trials (RCTs) but real-world cohorts and single-arm trials, we conducted two principled yet distinctive meta-analyses of the efficacy and safety differences between hypofractionated TRT (HypoTRT), conventional TRT (ConvTRT), and hyperfractionated TRT (HyperTRT) regimens, especially in the modern era. In the one-stage meta-analysis using 8006 reconstructed individual patient data (IPD) from 53 studies, the overall survival (OS) rates were similar between the three fractionation regimens. In the modern era, no significant differences in OS or severe radiation-related toxicities were observed between altered schedules. Results of the aggregated data (AD)-based network meta-analysis were consistent with those of the IPD analysis. The three TRT fraction regimens are acceptable options for LS-SCLC in the modern radiation era.

**Abstract:**

The optimal thoracic radiotherapy (TRT) dose and fractionation for limited-stage small cell lung cancer (LS-SCLC) using modern techniques remain unclear. We conducted systematic review and meta-analyses of the efficacy and safety differences between definitive hypofractionated TRT (HypoTRT), conventional TRT (ConvTRT) and hyperfractionated TRT (HyperTRT), especially in the modern era. Eligible randomized controlled trials (RCTs), real-world cohorts, and single-arm trials published between 1990 and 2021 were identified. Two meta-analyses of overall survival (OS) were conducted: (i) a random-effects meta-analysis based on reconstructed individual-patient data (IPD) of all studies; and (ii) a Bayesian network meta-analysis based on study-level aggregated data (AD) of RCTs. The incidences of severe radiation-related toxicities were compared using the random-effects meta-regression model. Overall, 53 of the 30,031 publications met the inclusion criteria, and a total of 8006 IPD were reconstructed. After adjusting for key treatment variables and stratification by study type, there were no significant differences in the OS rates between the altered fractionation regimens (HypoTRT vs. HyperTRT, aHR [adjusted HR] = 1.05, 95% CI 0.93–1.19; ConvTRT vs. HyperTRT, aHR = 1.00, 95% CI 0·90–1.11; HypoTRT vs. ConvTRT, aHR = 1.05, 95% CI 0.91–1.20). In the modern era, the survival outcomes of all three schedules, while remaining comparable, have improved significantly. Results of the AD-based network meta-analysis were consistent with those of IPD analysis, and HypoTRT was ranked as the best regimen (SUCRA = 81%). There were no significant differences in toxicities between groups when using modern radiation techniques. In the modern era, no significant differences in OS or severe radiation-related toxicities were observed between altered schedules in LS-SCLC. HypoTRT may be associated with moderate and non-significant OS improvements, which should be further confirmed in prospective randomized phase III trials.

## 1. Introduction

Thoracic radiotherapy (TRT), in combination with chemotherapy, is regarded as the standard treatment for inoperable limited-stage small cell lung cancer (LS-SCLC) [1,2,3]. Altered TRT dose and fractionation regimens may have meaningful impacts on survival and radiation-related adverse events due to the unique tumor biology characteristics of SCLC such as rapid doubling time and the accelerated proliferation of tumor cells [4,5,6].

The clinical benefits of a hyperfractionated twice-daily TRT (HyperTRT) regimen was first established by the landmark Intergroup 0096 phase III study in the 1990s [7]. Since then, significant progress in imaging and radiotherapy techniques have led to improved clinical outcomes and reduced toxicities in lung cancer as well as continued exploration of optimal TRT regimens [8,9]. Among these efforts, the CONVERT trial was a randomized phase III trial using modern and precise RT techniques, and demonstrated that comparable survival and toxicity outcomes did not differ between HyperTRT and conventional TRT (ConvTRT) [10]. Most recently, the randomized phase III trial CALGB 30610/RTOG 0538 showed that high-dose ConvTRT (70 Gy in 35 daily fractions) might be an acceptable dose and fractionation regimen, with comparable outcomes to HyperTRT [11]. Collectively, the optimal TRT dose and fractionation have not yet been well-established, especially in the modern radiotherapy era [12,13].

Simultaneously, the once-daily hypofractionated TRT (HypoTRT) regimen, which is delivered with a higher radiation dose per fraction within a shorter overall treatment time, has been proven feasible for LS-SCLC with the development of modern diagnostic and RT techniques [14,15,16,17,18,19,20]. Despite its radiobiological efficiency and convenience, currently, only a handful of randomized phase II trials focusing on HypoTRT have been completed, and there are no phase III trials to support its wide adoption.

To date, no randomized controlled trials (RCTs) have provided head-to-head comparisons between the three altered fractionation schedules. Several meta-analyses have been conducted to fill this evidence gap, however, their applicability and relevance to contemporary patients may be questionable [21,22]. One existing individual patient data (IPD) meta-analysis was conducted in the era of the outdated 2D radiotherapy technique and could not reflect its modern practice [21]. A more recent aggregated data (AD) meta-analysis, which compared once-daily and twice-daily schedules using five RCTs that ranged over three decades, inappropriately pooled time-to-event survival data as dichotomous outcomes [22]. 

Because high-quality and well-conducted systematic reviews and meta-analyses in this field are still warranted, we conducted a comprehensive systematic review involving not only RCTs but real-world cohorts and single-arm trials. Furthermore, we conducted two principled yet distinctive meta-analyses: (i) a one-stage meta-analysis using reconstructed IPD, and (ii) two-stage network meta-analysis of the study-level AD to compare the three altered fractionation regimens directly and indirectly. Considering the rapid development of radiotherapy delivery, we also explored whether the outcome discrepancies, if any, might vary with modern radiation techniques as a pre-planned subgroup analysis. 

## 2. Methods and Materials

### 2.1. Search Strategy and Eligibility Criteria

The meta-analyses were conducted following the Cochrane Collaboration and Preferred Reporting Items for Systematic Reviews and Meta-Analyses (PRISMA) guidelines (Supplementary Table S1). A literature search was performed across the PubMed, EMBASE, and Web of Science databases from 1 January 1990 to 31 July 2021. Studies presented at major conferences were also searched. The Supplementary Materials show the detailed search strategy (Figure S1).

The eligibility criteria were as follows: (1) diagnosis of LS-SCLC; (2) TRT with chemotherapy administered as curative intent; (3) RCTs, observational studies, or single arm phase II trials; and (4) separate and high-resolution Kaplan–Meier curves of overall survival (OS) for different fraction modalities. Additional eligibility criteria are presented in the Supplementary Materials (Method S1).

The eligible studies were classified into three categories: (1) RCTs; (2) comparative observational studies that directly compared different dose and fractionated schedules; and (3) single-fractionation prospective trials (aka prospective non-RCT studies).

### 2.2. Assessment of Study Quality 

Two reviewers assessed the quality of the included studies, and a third author resolved any disagreements in the assessments. The Cochrane Collaboration risk-of-bias-tool 2 (ROB 2), Newcastle–Ottawa scale (NOS), and methodological index for non-randomized studies (MINORS) criteria were used to evaluate the quality of the RCTs, observational studies, and single-arm trials, respectively. The quality assessment results of the included studies are summarized in the Supplementary Materials (Tables S2–S4). 

### 2.3. Data Extraction and IPD Reconstruction

Data were reviewed and extracted by two independent authors including the study characteristics, patient demographics, treatment details, and survival outcomes. Radiation esophagitis (RE) and radiation pneumonitis (RP) were recorded as primary radiation-related adverse events.

The R package (IPD from KM) was used to preprocess the raw data and reconstruct the IPD [23]. Raw data coordinates (time and survival probability) were extracted from published K–M survival curves using the Engauge Digitizer. The number of patients at risk and the total number of events were required for an accurate estimation if available. The accuracy of the reconstruction was quantified using several summary statistics including the root mean square error, maximum absolute error, mean absolute error, and Kolmogorov–Smirnov test [23]. The visualized and quantitative comparisons between the reconstructed and original curves are shown in the Supplementary Materials (Figure S2). 

### 2.4. Treatment Characteristics 

Eligible modalities of TRT were grouped into three types of fractionations: HypoTRT, ConvTRT, and HyperTRT (twice daily, with a minimum of 4–6 h between fractions). To account for the known prognostic impacts of overall treatment time, we calculated the biologically effective dose (BED) corrected for the time factor and adjusted in the analysis accordingly (Supplementary Method S2) [4,5].

### 2.5. Outcomes

The primary endpoint was OS, whose differences were quantified through hazard ratios (HRs) and the 95% confidence interval (CI). The secondary endpoint was the incidence of Grades 3–5 RE and RP. The Common Terminology Criteria for Adverse Events (CTCAE)/Radiation Therapy Oncology Group (RTOG) grading system was applied to assess the treatment related toxicities, as reported by each study. 

### 2.6. Data Analysis

A one-stage meta-analysis was employed to analyze OS using the reconstructed IPD. A random-effect shared frailty Cox proportional hazard model was used to account for the heterogeneity within and across studies, with non-parametric penalized likelihood estimation and spline smoothing for the baseline hazard functions [24,25]. To further account for the heterogeneity across different study types, a frailty model stratified by study type was used. Key study-level treatment factors were also adjusted as fixed effects in the aforementioned shared frailty Cox model. To confirm the robustness of the IPD meta-analysis, a Bayesian network meta-analysis was also performed based on the study-level AD of RCTs, which permits indirect comparisons of altered fractionation groups using existing pairwise comparisons of HypoTRT vs. HyperTRT, ConvTRT vs. HyperTRT, and HypoTRT vs. ConvTRT. The resulting HR and associated 95% Bayesian credible intervals (CrI) were presented for statistical inference (Supplementary Method S3). The R package frailtypack was used for one-stage IPD analysis, and gemtc was used for Bayesian network AD meta-analysis. 

The incidences of radiation-related toxicities between the TRT fraction modalities were compared using the random effects meta-regression model (Supplementary Method S3) [26,27]. The R package meta and metafor were used for the analysis of adverse events. A *p*-value < 0.05 was considered statistically significant. All analyses were performed using R Studio (version 4.0.5). The study protocol was prospectively registered in PROSPERO (CRD42022343063).

### 2.7. Subgroup Analysis

Subgroup analyses were conducted to assess if and how findings may differ across study types, especially focusing on analysis in the subset of patients who underwent modern and precise techniques including three-dimensional conformal radiotherapy or intensity-modulated radiotherapy (3D-CRT/IMRT).

## 3. Results

### 3.1. Study Selection and Characteristics

**Figure 1:**
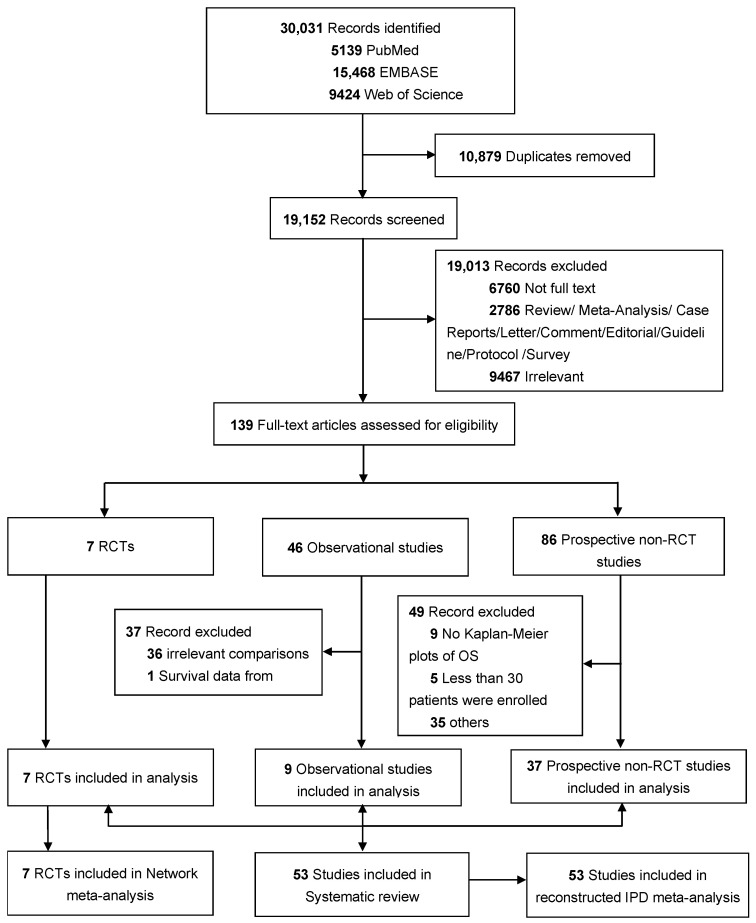
Study selection. Abbreviations: RCT, randomized controlled trials; OS, overall survival; NCDB, National Cancer Database; IPD, individual patient data.

The systematic review retrieved 19,152 studies from a total of 30,031 after removing duplicates. A total of 138 full-text articles and one conference abstract were carefully reviewed and divided into three predefined subgroups depending on the study design and comparative information: RCTs, observational studies, and prospective non-RCT studies. Among them, 86 papers were excluded and the detailed reasons are displayed in Figure 1.

Ultimately, a total of 53 studies with 8006 patients were identified from 30,031 study records; the PRISMA flow diagram is shown in Figure 1. Among the enrolled studies, seven [7,10,11,18,20,28,29] were classified into the RCTs category, nine [14,15,16,17,30,31,32,33,34] into the comparative observational studies category, and 37 [35,36,37,38,39,40,41,42,43,44,45,46,47,48,49,50,51,52,53,54,55,56,57,58,59,60,61,62,63,64,65,66,67,68,69,70,71] into prospective non-RCT studies. A total of 1689 (21%) patients from 13 studies were treated with HypoTRT, 3118 (39%) patients from 27 studies were treated with ConvTRT, and 3199 (40%) patients from 29 studies were treated with HyperTRT. 3D-CRT or IMRT was used in 21 studies with 3493 (44%) patients. Table 1 summarizes the patient demographics and treatment details of the enrolled studies [7,10,11,14,15,16,17,18,20,28,29,30,31,32,33,34,35,36,37,38,39,40,41,42,43,44,45,46,47,48,49,50,51,52,53,54,55,56,57,58,59,60,61,62,63,64,65,66,67,68,69,70,71]. Detailed descriptions of the main characteristics of each study are provided in Supplementary Table S5.

Based on the study-level AD, 5178 (65%) participants were male and 2828 (35%) were female. A total of 91% patients underwent concurrent chemoradiotherapy (CCRT) in the all included studies and 100% in the RCTs. A total of 52% patients received TRT within the first two cycles of induction chemotherapy, while the proportion varied widely across study types (81% in RCTs, 12% in comparative observational studies, and 47% in prospective non-RCT studies). The use of prophylactic cranial irradiation (PCI) ranged from 12% to 100% across studies. Most studies in the HypoTRT group were administered 2.5–3.0 Gy per fraction once daily, with a total dose ranging from 37.5 Gy to 65.0 Gy, which corresponded to 39.04–66.40 Gy corrected BED10. The total dose administered in the ConvTRT group was 45.0–70.0 Gy of 1.8–2.1 Gy per fraction, and the corrected BED10 ranged from 39.49 to 64.61 Gy. Studies in the HyperTRT group was delivered with 45.0–60.0 Gy twice daily (1.4–1.5 Gy per fraction) with a minimum of 4–6 h between fractions, and the corrected BED10 ranged from 39.53 Gy to 58.28 Gy.

### 3.2. IPD Meta-Analysis

**Figure 2:**
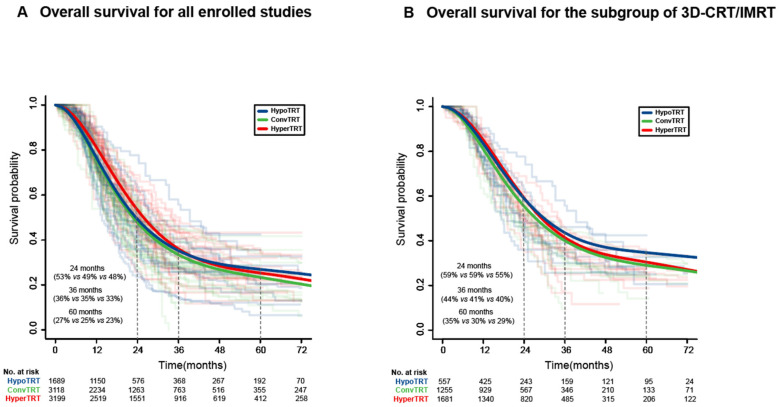
Individual patient data frailty model survival curves of the different fractionated radiotherapy, along with those from each study. (**A**), Overall survival for all enrolled studies; (**B**), Overall survival for the subgroup of 3D-CRT/IMRT. Abbreviations: 3D-CRT, three-dimensional conformal radiotherapy; IMRT, intensity-modulated radiotherapy; HypoTRT, hypofractionated thoracic radiotherapy; ConvTRT, conventional fractionated thoracic radiotherapy; HyperTRT, hyperfractionated thoracic radiotherapy.

**Figure 3:**
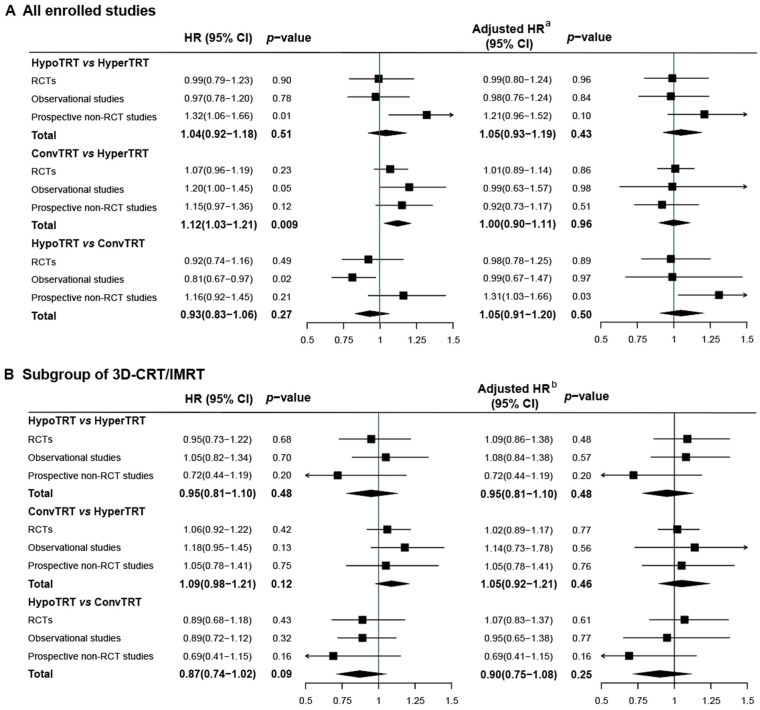
Forest plots showing the hazard ratio estimates based on the shared-frailty model. (**A**), Forest plot for all enrolled studies; (**B**), Forest plot for subgroup of 3D-CRT/IMRT. Abbreviations: HR, hazard ratio; CI, confidence interval; HypoTRT, hypofractionated thoracic radiotherapy; ConvTRT, conventional fractionated thoracic radiotherapy; HyperTRT, hyperfractionated thoracic radiotherapy; RCTs, randomized controlled studies; 3D-CRT, three-dimensional conformal radiotherapy; IMRT, intensity-modulated radiotherapy. ^a^^,b^ Adjusted for concurrent chemoradiotherapy, thoracic radiotherapy timing, and corrected biologically effective dose (all characteristics were regarded as binary variables).

For the entire cohort of 8006 patients from 53 studies, the median follow-up was 60.0 months (IQR 40.67–88.23), with a total of 5795 deaths occurring. Based on the shared frailty Cox model stratified by study types, the estimated survival curves of each fractionation regimen, along with those from each study, are shown in Figure 2A. The overall 2-, 3-, and 5-year survival rates were 49%, 35%, and 27% in the HypoTRT group, 48%, 33%, and 23% in the ConvTRT group, and 53%, 36%, and 25% in the HyperTRT group, respectively. The OS rates were comparable between HypoTRT and HyperTRT (HR = 1.04, 95% CI 0.92–1.18) or ConvTRT (HR = 0.93, 95% CI 0.83–1.06); meanwhile ConvTRT was inferior to HyperTRT (HR = 1.12, 95% CI 1.03–1.21, Figure 3A). However, after adjusting for the corrected BED10, CCRT, and TRT timing [5,10,67,72], the OS rates became similar between the three groups (HypoTRT vs. HyperTRT, adjusted HR = 1.05, 95% CI 0.93–1.19; ConvTRT vs. HyperTRT, adjusted HR = 1.00, 95% CI 0.90–1.11; HypoTRT vs. ConvTRT, adjusted HR = 1.05, 95% CI 0.91–1.20, Figure 3A). 

### 3.3. AD Network Meta-Analysis

**Figure 4:**
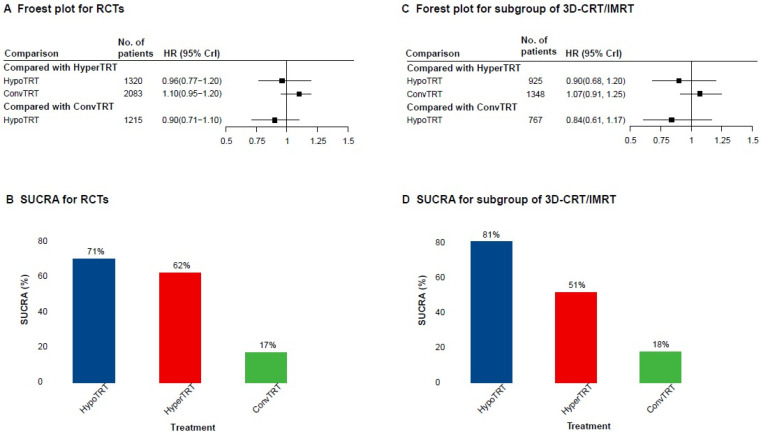
Forest plots for the hazard ratio and ranking plots with the surface under the cumulative ranking curve (SUCRA) in the network meta-analysis. (**A**), Forest plot for RCTs; (**B**), Forest plot for subgroup of 3D/CRT-IMRT; (**C**), SUCRA for RCTs; (**D**), SUCRA for subgroup of 3D-CRT/IMRT. Abbreviations: RCT, randomized controlled studies; HR, hazard ratio; CrI, credible interval; HypoTRT, hypofractionated thoracic radiotherapy; ConvTRT, conventional fractionated thoracic radiotherapy; HyperTRT, hyperfractionated thoracic radiotherapy; 3D-CRT, three-dimensional conformal radiotherapy; IMRT, intensity-modulated radiotherapy; SURA, surface under the cumulative ranking curve.

Similar results were found when using a Bayesian network meta-analysis based on RCTs (Supplementary Figure S3). Results from the direct comparisons are summarized in the Supplementary Materials (Table S6). The OS rates were similar between HypoTRT, ConvTRT, and HyperTRT, respectively (HypoTRT vs. HyperTRT, HR = 0.96, 95% CrI 0.77–1.20; ConvTRT vs. HyperTRT, HR = 1.10, 95% CrI 0.95–1.20; HypoTRT vs. ConvTRT, HR = 0.90, 95% CrI 0.71–1.10; Figure 4A). The results of the ranking plot with the surface under the cumulative ranking curve (SUCRA), HypoTRT (SUCRA = 71%), and HyperTRT (SUCRA = 62%) were found to be most likely the best among the three regimens (Figure 4B). Furthermore, no treatment factors were found to be significantly associated with OS (Supplementary Table S7).

### 3.4. Subgroup Analysis of 3D-CRT or IMRT

Among 3493 patients treated with modern radiotherapy, 2281 deaths occurred over a follow-up period of 55.10 months (IQR 36.10–71.90). In total, 557 patients across eight studies were allocated to the HypoTRT group, 1255 patients across 10 studies to the ConvTRT group, and 1681 patients across 14 studies to the HyperTRT group. 

Using a one-stage random-effect meta-analysis model based on reconstructed IPD, we found that the 2-, 3-, and 5-year OS rates were 59%, 44%, and 35% in the HypoTRT group, 55%, 40%, and 29% in the ConvTRT group, and 59%, 41%, and 30% in the HyperTRT group, respectively (Figure 2B). The OS results with the HypoTRT (HR = 0.51, 95% CI 0.36–0.73), ConvTRT (HR = 0.84, 95% CI 0.70–0.99), and HyperTRT regimens (HR = 0.95, 95% CI 0.79–1.15) (HR = 0.95, 95% CI 0.79–1.15) were significantly higher than those in the 2D era.

HyperTRT was comparable with either HypoTRT or ConvTRT in the OS rates (HypoTRT vs. HyperTRT, HR = 0.95, 95% CI 0.81–1.10; ConvTRT vs. HyperTRT, HR = 1.09, 95% CI 0.98–1.21). HypoTRT was associated with a marginally higher OS than ConvTRT (HR = 0.87, 95%CI 0.74–1.02) (Figure 3B). After adjusting the aforementioned treatment characteristics, these conclusions did not change (HypoTRT vs. HyperTRT, adjusted HR = 0.95, 95% CI 0.81–1.10; ConvTRT vs. HyperTRT, adjusted HR = 1.05, 95% CI 0.92–1.21; HypoTRT vs. ConvTRT, adjusted HR = 0.90, 95%CI 0.75–1.08) (Figure 3B). 

Similar findings of pairwise comparisons in the two-stage Bayesian network meta-analysis were observed (Figure 4C). In terms of the results from the ranking plot with SUCRA, the beneficial orders for OS from the greatest to the least were HypoTRT (SUCRA = 81%), HyperTRT (SUCRA = 51%), and ConvTRT (SUCRA = 18%) (Figure 4D). 

### 3.5. Incidences of RE and RP

A total of 44 studies that reported severe RE (10, 20, and 26 in HypoTRT, ConvTRT, and HyperTRT, respectively) were included. The rates of pooled grades 3–5 RE incidence were 9% (95% CI 4–16), 11% (95% CI 7–16), and 18% (95% CI 13–23) for the HypoTRT, ConvTRT, and HyperTRT group, respectively. Meta-regression analysis showed that severe (grades 3–5) RE incidence were similar between HypoTRT and ConvTRT (*p* = 0.62) while there was a higher incidence with HyperTRT (vs HypoTRT, *p* = 0.03; vs. ConvTRT, *p* = 0.04). A total of 38 studies reported severe grade 3–5 RP (9, 18, and 23 in HypoTRT, ConvTRT, and HyperTRT, respectively). The rates of severe RP were 3% (95% CI 1–6), 4% (95% CI 2–6), and 3% (95% CI 2–5), respectively, which were similar across the different groups (Supplementary Figure S4). There was no publication bias for the enrolled studies (RE, *p* = 0.49; RP, *p* = 0.08) (Supplementary Figure S5).

In the modern era, there is no pronounced difference in either severe RE (HypoTRT vs. HyperTRT, 14% vs. 17%, *p* = 0.49; ConvTRT vs. HyperTRT, 12% vs. 17%, *p* = 0.21; HypoTRT vs. ConvTRT, 14% vs. 12%, *p* = 0.77) or RP (HypoTRT vs. HyperTRT, 5% vs. 3%, *p* = 0.24; ConvTRT vs. HyperTRT, 5% vs. 3%, *p* = 0.30; HypoTRT vs. ConvTRT, 5% vs. 5%, *p* = 0.95) (Supplementary Figure S4). 

Additionally, a total of ten studies have reported the incidence of late grade 3–5 RE and RP (supplementary Table S8). The incidence of the most frequently recorded event for the overall cohort was no more than 5%. Only one earlier study utilizing the 2D technique and HypoTRT regimen reported an extremely high risk of severe late lung toxicity (38%) [41]. 

## 4. Discussion

To our best knowledge, this is the first and largest meta-analysis (8006 patients) of reconstructed time-to-event analysis of TRT fractionation for LS-SCLC. In this systematic review and meta-analyses, HyperTRT yielded similar survival outcomes as ConvTRT with advanced radiotherapy techniques, which confirms the generalizability of the existing landmark phase III RCTs by synthesizing evidence across all study types [10,11]. Our findings further support that HypoTRT should be considered as an acceptable regimen, which provide powerful evidence for the updated recommendations in the latest NCCN guidelines, especially in the absence of phase III RCTs [13]. It is worth noting that in the network meta-analysis, HypoTRT was ranked the best regimen, far beyond the two other modalities within the 3D-CRT/IMRT subgroup (SUCRA = 81%). Therefore, HypoTRT is an attractive alternative because of its favorable treatment effects, toxicity tolerance, decreased resource utilization, and patient convenience. 

Several unique features make our work distinct from the existing meta-analysis in this field. The Meta-Analysis of Radiotherapy in Lung Cancer (MAR-LC) collaborative group conducted an IPD meta-analysis based on two trials published in the 1990s. The results showed a non-significant difference in OS between HyperTRT and ConvTRT (3- and 5- year OS rates, 31% vs. 30% and 24% vs. 22%, HR= 0.87, 95% CI 0.74–1.02) and an increased incidence of acute severe RE in the HyperTRT regimen (HyperTRT vs. ConvTRT, 25% vs. 12%, *p* < 0.01) when non-contemporary radiotherapy techniques were used [21]. In contrast, our work showed more recent and relevant results with significantly improved OS (ConvTRT vs. HyperTRT, 3- and 5- year OS rates, 40% vs. 41% and 29% vs. 30%, adjusted HR = 1.05, 95% CI 0.92–1.21) and a decreased incidence of severe RE in the HyperTRT group (vs ConvTRT, 17% vs. 12%, *p* = 0.21) in the modern era. More recently, Viani et al. reported a meta-regression analysis based on the study-level information of five RCTs with limited sample size, where the HypoTRT group consisted of only 172 patients from two phase II trials. They concluded that the OS rates were similar between ConvTRT and HyperTRT, while HypoTRT yielded more survival benefits (HyperTRT vs. HypoTRT, HR = 1.45, or equivalently, HypoTRT vs. HyperTRT, HR = 0.69, *p* = 0.03) in the subgroup analysis [22]. In contrast, our two-stage analysis based on study-level information yielded a consistent, although somewhat attenuated effect of HypoTRT when compared with HyperTRT in the modern era (HR = 0.90, 95% CrI 0.68–1.20). When including more studies and expanding the study types to observational cohorts and non-randomized trials, our findings may represent less optimistic but probably more realistic benefits of HypoTRT in the real-world after accounting for other treatment characteristics including corrected BED10. Nevertheless, a moderate survival benefit of HypoTRT, coupled with the favorable safety profile and treatment convenience, could still be a preferred option that warrants prospective randomized phase III trials to confirm its true benefit/risk profile.

Apart from its inclusivity and large sample size, a key strength of the present work was our use of a reconstructed time-to-event IPD to granularly assess the survival differences between altered fractionations. An IPD meta-analysis is highly desirable in evidence synthesis, and plays a critical role in defining practice in the absence of large, randomized trials [73,74]. When carefully conducted, reconstructed IPD provides an alternative approach to access individual time-to-event survival data from an unrestricted pool of studies. To robustly evaluate the comparative effectiveness of the three fractionation regimens, we included not only RCTs but real-world cohorts and single-fractionation prospective trials in one-stage IPD analysis. This approach not only mitigated the challenges due to the limited number of RCTs, but also enhanced the generalizability and relevance of our findings in the modern era. Admittedly, the heterogeneity caused by the reported and unreported covariates cannot be ignored. Several measures were taken to mitigate such a risk. First, the shared frailty model stratified by study type was used for one-stage IPD analysis that accounted for the heterogeneity within and across studies. Second, correction for study-level key factors reduced the bias caused by various treatment characteristics, especially important for prospective trials without related head-to-head comparisons in TRT fractionation. Third, given the value of the time factor in rapidly growing SCLC, we calculated and adjusted for the time corrected BED10, which allowed for the comparisons between the altered fractionation schedules [5]. In addition, a two-stage approach was used for a validation analysis, and the results confirmed our conclusions from the study-level analysis. Furthermore, considering the long time-span of the enrolled studies, we performed a subgroup analysis in the modern era for both the survival and toxicity files. Notably, regarding severe RE, increased rates were observed in HypoTRT (9% change to 14%) and ConvTRT (11% change to 12%) in the 3D-CRT/IMRT subgroup, and the differences narrowed between the three regimens (HypoTRT vs. HyperTRT, 9% vs. 18%, *p* = 0.03 change to 14% vs. 17%, *p* = 0.49; ConvTRT vs. HyperTRT, 11% vs. 18%, *p* = 0.04 change to12% vs. 17%, *p* = 0.21). This might be explained in part by the decrease in the incidence of severe RE in the HyperTRT group in the modern radiation era, and on the other hand, the application of advanced RT techniques allowing for a higher TRT dose delivery in the HypoTRT and ConvTRT groups, which could cause a higher toxicity incidence. 

There were some limitations in this work. First, although the reconstructed IPD provides an accurate estimation of individual patient time-to-event, this algorithm is limited in its ability to obtain additional patient-level characteristics. To correct for treatment-related confounding factors and accommodate for the heterogeneity across studies, we extracted some important study-level covariates including the corrected BED10, CCRT, TRT timing, and radiation technique. However, these statistical adjustments may or may not adequately isolate their impacts when estimating the possibly incremental but clinically meaningful improvements due to HypoTRT. Second, because the toxicities were reported inconsistently across all studies, we chose to focus on grade 3–5 RE and RP and thus exclude studies reporting symptomatic or any grade of toxicities. Third, acute and late radiation-related toxicities, which potentially reflect different tissue responses to modified fractionation, have not been listed separately and clearly in some studies, which may cause bias in event statistics. Fourth, considering that there are so many factors associated with the occurrence of hematologic toxicities, especially the chemotherapy regimens, which had the greatest impact in pancytopenia, we did not summarize the incidence of hematologic toxicities in this research. Finally, the various study types in the enrolled literature potentially increased the heterogeneity. Although a number of measures have been taken, the inherent biases of enrollment such as population selection bias, attrition, and data quality cannot be completely eliminated by study design or statistical methods. 

To our knowledge, a phase III multicenter RCT is currently underway to compare the effects between HypoTRT (45 Gy in 15 fractions over three weeks) and ConvTRT (60 Gy in 30 fractions over six weeks) concomitant with chemotherapy for inoperable LS-SCLC (NCT02688036), which will provide robust evidence for the effect of HypoTRT using advanced techniques. Similarly, the final results of the CALGB 30610/RTOG 0538 trial, which compares high-dose ConvTRT and standard HyperTRT, are expected to be reported soon. 

## 5. Conclusions

In conclusion, HypoTRT, ConvTRT, and HyperTRT had comparable survival outcomes in LS-SCLC, while HyperTRT was associated with higher rates of severe RE. In the modern radiation therapy era, no significant differences in OS rates and severe radiation-related adverse events were observed between the altered schedules. HypoTRT may be associated with a moderate and non-significant survival benefit while prospective randomized phase III trials are warranted. 

## Figures and Tables

**Table 1:** Characteristics of the studies and participants.

	HypoTRT			ConvTRT			HyperTRT			Total			
Study Types	RCTs	Obs	P non-RCT	RCTs	Obs	P non-RCT	RCTs	Obs	P non-RCT	RCTs	Obs	P non-RCT	All Types
Studies	3	5	5	5	7	15	6	6	17	7	9	37	53
Participants	226	326	1137	989	508	1621	1094	351	1754	2309	1185	4512	8006
Sex, No. (%)													
Male	159 (70)	193 (59)	767 (67)	531 (54)	357 (70)	1044 (64)	615 (56)	216 (62)	1296 (74)	1305 (57)	766 (65)	3107 (69)	5178 (65)
Female	67 (30)	133 (41)	370 (33)	458 (46)	151 (30)	577 (36)	479 (44)	135 (38)	458 (26)	1004 (43)	419 (35)	1405 (31)	2828 (35)
Median age, years	58–63	59–69	58–62	63	55–71	49–66	58–64	54–66	54–66	58–64	54–71	49–66	49–71
TRT technique, No. (%)													
2DRT	54 (24)	0	874 (77)	394 (40)	172 (34)	651 (40)	341 (31)	37 (11)	493 (28)	789 (34)	209 (18)	2018 (45)	3016 (38)
3D-CRT/IMRT	172 (76)	326 (100)	59 (5)	595 (60)	336 (66)	324 (20)	753 (69)	314 (89)	614 (35)	1520 (66)	976 (82)	997 (22)	3493 (44)
Unreported	0	0	204 (18)	0	0	646 (40)	0	0	647 (37)	0	0	1497 (33)	1497 (19)
Corrected BED_10_, No. (%)													
High-dose group	226 (100)	270 (83)	736 (65)	651 (66)	0	201 (12)	964 (88)	351 (100)	1754 (100)	1841 (80)	621 (52)	2691 (60)	5153 (64)
Low-dose group	0	56 (17)	401 (35)	338 (34)	508 (100)	1420 (88)	130 (12)	0	0	468 (20)	564 (48)	1821 (40)	2853 (36)
CCRT, No. (%)													
Yes	226 (100)	226 (69)	972 (85)	989 (100)	325 (64)	1546 (95)	1094 (100)	351 (100)	1583 (90)	2309 (100)	902 (76)	4101 (91)	7312 (91)
No/unreported	0	100 (31)	165 (15)	0	183 (36)	75 (5)	0	0	171 (10)	0	283 (24)	411 (9)	694 (9)
TRT timing, No. (%)													
Yes	138 (61)	111 (34)	588 (52)	857 (87)	0	550 (34)	870 (80)	26 (7)	997 (57)	1865 (81)	137 (12)	2135 (47)	4137 (52)
No/unreported	88 (39)	215 (66)	549 (48)	132 (13)	508 (100)	1071 (66)	224 (20)	325 (93)	757 (43)	444 (19)	1048 (88)	2377 (53)	3869 (48)
PCI completion rates (%)	51	52–67	64–100	60–85	21–67	12–81	81	54–65	32–90	51–85	21–67	12–100	12–100
Median follow-up (months)	14.7–59	20.4–162	19.5–60	14.7–96	22–67	15–69	24.3–96	20.4–34	16.3–75.6	14.7–96	20.4–162	15–75.6	14.7–162

Abbreviations: HypoTRT, hypofractionated thoracic; ConvTRT, conventional fractionated thoracic radiotherapy; HyperTRT, hyperfractionated thoracic radiotherapy; RCTs, randomized controlled trials; Obs, observational studies; P non-RCT, prospective non-RCT studies; TRT, thoracic radiotherapy; 2DRT, two-dimensional radiotherapy; 3D-CRT, three-dimensional conformal radiotherapy; IMRT, intensity-modulated radiotherapy; BED_10_, biologically effective dose; CCRT, concurrent chemoradiotherapy; PCI, prophylactic cranial irradiation.

## Supplementary Materials

The following supporting information can be downloaded at: https://www.mdpi.com/article/10.3390/cancers15010277/s1, Method S1: Eligibility criteria; Method S2: Data extraction; Method S3: Statistical analysis; Table S1: PRISMA checklist; Table S2: Risk of bias assessment according to ROB-2; Table S3: Risk of bias assessment according to NOS; Table S4: Risk of bias assessment according to the MINORS criteria; Table S5: Characteristics of each enrolled study; Table S6: Summary of the results from direct comparisons for overall survival; Table S7: Network meta-regression results; Table S8: Late severe radiation-related adverse events; Figure S1: Search strategy; Figure S2: Comparisons of the reconstructed and original curves; Figure S3: Network plots; Figure S4: Forest plots of severe radiation related events for altered fractionation modalities; Figure S5: Funnel plot according to the double arcsine transformed proportion for publication bias.
