# Supplementary material for: Radiotherapy Fraction in Limited-Stage Small Cell Lung Cancer in the Modern Era: A Systematic Review and Meta-Analysis of 8006 Reconstructed Individual Patient Data
Source: Cancers (Basel). 2022 Dec 31;15(1):277. doi: 10.3390/cancers15010277 (PMC9818135; doi:10.3390/cancers15010277)
Supplement: Supplementary file 1 [file cancers-15-00277-s001.zip › cancers-2082809-supplementary.pdf]

Supplementary Online Content

Radiotherapy fraction in limited-stage small cell lung cancer in the modern era: a systematic review and meta-analysis of 8006 reconstructed individual patient data

Methods

Method S1. Eligibility criteria
Method S2. Data extraction
Method S3. Statistical analysis

Tables

Table S1. PRISMA checklist.
Table S2. Risk of bias assessment according to ROB-2.
Table S3. Risk of bias assessment according to NOS.
Table S4. Risk of bias assessment according to MINORS criteria.
Table S5. Characteristics of each enrolled study.
Table S6. Summary of results from direct comparisons for overall survival
Table S7. Network meta-regression results.
Table S8. Late severe radiation-related adverse events.

Figures

Figure S1. Search strategy.
Figure S2. Comparisons of reconstructed and original curves
Figure S3. Network plots.
Figure S4. Forest plots of severe radiation related events for altered fractionation modalities.
Figure S5. Funnel plot according to double arcsine transformed proportion for publication bias

Supplementary References

Method S1. Eligibility criteria

To avoid re-analyses or overlapping populations, we selected more pertinent results from those reporting both interim and long-term follow-up outcomes. Survival data obtained from public databases such as the National Cancer Database (NCDB) or Surveillance, Epidemiology, and End Results (SEER) were excluded for fear of potential duplication. For publications that used propensity scores, stratification or covariance adjustment to control for confounding factors, we adopted initial data considering the ineffective balancing effect when delivering meta-analysis employed reconstructed individual patient data (IPD). Furthermore, studies reporting survival endpoints with a follow-up of less than 12 months were also excluded on due to the insufficient overall survival (OS) outcomes within the short duration.

Method S2. Data extraction

Several treatment characteristics, including radiation techniques, concurrent chemoradiotherapy (CCRT) (yes or no), and whether thoracic radiotherapy (TRT) was started within two cycles of induction chemotherapy were extracted as binary variables for analysis. Owing to the mixed quality of the enrolled studies, sometimes the original article did not provide precise information and the aforementioned variables could not be converted to binary classifications as expected. In these cases, if the proportion the population receiving CCRT or TRT started within two cycles induction chemotherapy ≥ 70% in the literature, we assigned them into the "yes" group in study level. Simultaneously, only studies that clearly described using three-dimensional conformal radiotherapy (3D-CRT) or intensity-modulated radiotherapy (IMRT) were regarded as applying modern radiation techniques.

A linear-quadratic equation with a basic time factor to allow for proliferation was used to calculate the biologically effective dose (BED): BED = (nd) [1+d / (α/β)] – ($0.693t/\alpha T_{pot}$), where n is the total number of fractions delivered; d is the fraction size; α/β = 10; α = 0.3 Gy; t is total irradiation days; and T_{pot} = potential doubling time (5.6 days in small-cell lung cancer) [1-3]. Based on the recommended dose and fractionation of HyperTRT (45 Gy in 30 twice-daily fractions over 19-21days), ConvTRT (66-70 Gy in 6.5-7 weeks/2 Gy daily), and HypoTRT (40-42 Gy in 3 weeks given in once-daily fractionation), we calculated the corrected BED_{10} for the three regimens: 43.09-43.91 Gy, 60.64-64.61 Gy, and 40.93-45.38 Gy, respectively [4]. The enrolled patients were divided into two groups according to the corrected BED_{10}: high-dose group (corrected BED_{10} ≥ 43.09 Gy in the HyperTRT group, ≥ 60.64 Gy in the ConvTRT group, and ≥ 40.93 Gy in the HypoTRT group) and low-dose group (corrected BED_{10} < 43.09 Gy in the HyperTRT group, < 60.64 Gy in the ConvTRT group, and < 40.93 Gy in the HypoTRT group).

Method S3. Statistical analysis

To perform the Bayesian network meta-analysis, we calculated the logarithm of the hazard ratios (HRs) and standard errors for each study to estimate the pooled HRs [5]. Both fixed- and random-effects models were used. In view of the consistent results, we finally reported the results from the fixed-effects model with narrower 95% CrI (credible interval). R package *gemtc* employed Markov Chain Monte Carlo algorithms for treatment effects estimation, with four parallel Markov chains consisting of 100000 samples after a 50000-sample burn-in. We calculated the surface under the cumulative probability ranking (SUCRA) to measure the effects of various fractionation schedules. Higher SUCRA value indicates a higher ranking, as well as higher efficacy of the fractionation radiotherapy regimen. In addition, network meta-regression was conducted to evaluate whether treatment characteristics influenced these effects.

The results are presented as proportions of the incidence of severe radiation esophagitis (RE) and radiation pneumonitis (RP). Heterogeneity was assessed using the Higgins' I^2 statistics and the Cochran Q test [6]. Significant heterogeneity was considered present if the I^2 statistic was > 50%, the P-value of the Q test was < 0.05; the random-effects model with inverse variance weighting was used in this case. Since severe toxicity was not common and zero adverse events were reported in several studies, we used a Freeman-Tukey Double arcsine transformation for the meta-analysis of signal rates with zero events [7]. Forest plots were generated based on this transformation for the grade 3-5 adverse events in different TRT fraction regimen. Publication bias was assessed using the Egger test, and Duval and Tweedie's trim-and-fill method was performed to assess potential bias. We did a sensitivity analysis to explore the robustness of the results using the leave-one-

out method. Meta-regression was used to estimate the differences in the incidence of toxicity in various fractionated schedules. The median follow-up time was calculated by the reverse Kaplan-Meier method

Table S1. PRISMA checklist.

PRISMA Main Checklist

Topic	No.	Item	Location where item is reported
**TITLE**			
**Title**	1	Identify the report as a systematic review.	Page 1 main MS
**ABSTRACT**			
**Abstract**	2	See the PRISMA 2020 for Abstracts checklist	
**INTRODUCTION**			
**Rationale**	3	Describe the rationale for the review in the context of existing knowledge.	Page 2 main MS
**Objectives**	4	Provide an explicit statement of the objective(s) or question(s) the review addresses.	Page 2 main MS
**METHODS**			
**Eligibility criteria**	5	Specify the inclusion and exclusion criteria for the review and how studies were grouped for the syntheses.	Page 3 main MS; Methods S1 in the supplement
**Information sources**	6	Specify all databases, registers, websites, organisations, reference lists and other sources searched or consulted to identify studies. Specify the date when each source was last searched or consulted.	Page 3 main MS; Figure S1 in the supplement
**Search strategy**	7	Present the full search strategies for all databases, registers and websites, including any filters and limits used.	Figure S1 in the supplement
**Selection process**	8	Specify the methods used to decide whether a study met the inclusion criteria of the review, including how many reviewers screened each record and each report retrieved, whether they worked independently, and if applicable, details of automation tools used in the process.	Page 3 main MS; Method S1 in the supplement

Topic	No.	Item	Location where item is reported
**Data collection process**	9	Specify the methods used to collect data from reports, including how many reviewers collected data from each report, whether they worked independently, any processes for obtaining or confirming data from study investigators, and if applicable, details of automation tools used in the process.	Page 3 main MS; Method S2 and Figure S2 in the supplement
**Data items**	10a	List and define all outcomes for which data were sought. Specify whether all results that were compatible with each outcome domain in each study were sought (e.g. for all measures, time points, analyses), and if not, the methods used to decide which results to collect.	Page 3 main MS; Method S3 in the supplement
	10b	List and define all other variables for which data were sought (e.g. participant and intervention characteristics, funding sources). Describe any assumptions made about any missing or unclear information.	Page 3 main MS; Method S2 in the supplement
**Study risk of bias assessment**	11	Specify the methods used to assess risk of bias in the included studies, including details of the tool(s) used, how many reviewers assessed each study and whether they worked independently, and if applicable, details of automation tools used in the process.	Page 4 main MS
**Effect measures**	12	Specify for each outcome the effect measure(s) (e.g. risk ratio, mean difference) used in the synthesis or presentation of results.	Page 4 main MS
**Synthesis methods**	13a	Describe the processes used to decide which studies were eligible for each synthesis (e.g. tabulating the study intervention characteristics and comparing against the planned groups for each synthesis (item 5)).	Page 4 main MS; Table S6, Figure 1 in the supplement
	13b	Describe any methods required to prepare the data for presentation or synthesis, such as handling of missing summary statistics, or data conversions.	Page 4 main MS; Method S2, Method S3 in the supplement

Topic	No.	Item	Location where item is reported
	13c	Describe any methods used to tabulate or visually display results of individual studies and syntheses.	Page 4 main MS; Method S3, Figure S2 in the supplement
	13d	Describe any methods used to synthesize results and provide a rationale for the choice(s). If meta-analysis was performed, describe the model(s), method(s) to identify the presence and extent of statistical heterogeneity, and software package(s) used.	Page 4 main MS; Method S3 in the supplement
	13e	Describe any methods used to explore possible causes of heterogeneity among study results (e.g. subgroup analysis, meta-regression).	Page 4main MS; Method S3 in the supplement
	13f	Describe any sensitivity analyses conducted to assess robustness of the synthesized results.	Page 4 main MS; Method S3 in the supplement
**Reporting bias assessment**	14	Describe any methods used to assess risk of bias due to missing results in a synthesis (arising from reporting biases).	Method S3 in the supplement
**Certainty assessment**	15	Describe any methods used to assess certainty (or confidence) in the body of evidence for an outcome.	Page 4 main MS; Method S3 in the supplement
**RESULTS**			
**Study selection**	16a	Describe the results of the search and selection process, from the number of records identified in the search to the number of studies included in the review, ideally using a flow diagram.	Page 4 main MS; Figure 1
	16b	Cite studies that might appear to meet the inclusion criteria, but which were excluded, and explain why they were excluded.	Page 4 main MS; Figure 1
**Study characteristics**	17	Cite each included study and present its characteristics.	Page 5 main MS; Table S5 in the supplement
**Risk of bias in studies**	18	Present assessments of risk of bias for each included study.	Table S2-4 in the supplement

Topic	No.	Item	Location where item is reported
**Results of individual studies**	19	For all outcomes, present, for each study: (a) summary statistics for each group (where appropriate) and (b) an effect estimate and its precision (e.g. confidence/credible interval), ideally using structured tables or plots.	Page 6 main MS; Table 1; Table S5 in the supplement
**Results of syntheses**	20a	For each synthesis, briefly summarise the characteristics and risk of bias among contributing studies.	Page 7-10 main MS; Table S6, 8 and Figure S5 in the supplement
	20b	Present results of all statistical syntheses conducted. If meta-analysis was done, present for each the summary estimate and its precision (e.g. confidence/credible interval) and measures of statistical heterogeneity. If comparing groups, describe the direction of the effect.	Page 7-10 main MS; Table S7 and Figure S4 in the supplement; Figure 2-4
	20c	Present results of all investigations of possible causes of heterogeneity among study results.	Page 8-10 main MS; Table S7 and Figure S5 in the supplement
	20d	Present results of all sensitivity analyses conducted to assess the robustness of the synthesized results.	Page 8 main MS; Table S6-7; Figure S3 in the supplement; Figure 4
**Reporting biases**	21	Present assessments of risk of bias due to missing results (arising from reporting biases) for each synthesis assessed.	Page 10 main MS; Figure S5 in the supplement
**Certainty of evidence**	22	Present assessments of certainty (or confidence) in the body of evidence for each outcome assessed.	Page 7-10 main MS; Table S6-7 and Figure S4 in the supplement; Figure 3, 4
**DISCUSSION**			
**Discussion**	23a	Provide a general interpretation of the results in the context of other evidence.	Page 10 main MS
	23b	Discuss any limitations of the evidence included in the review.	Page 11, 12 main MS
	23c	Discuss any limitations of the review processes used.	Page 11, 12 main MS
	23d	Discuss implications of the results for practice, policy, and future research.	Page 10-12 main MS

Topic	No.	Item	Location where item is reported
**OTHER INFORMATION**			
**Registration and protocol**	24a	Provide registration information for the review, including register name and registration number, or state that the review was not registered.	Page 4 main MS
	24b	Indicate where the review protocol can be accessed, or state that a protocol was not prepared.	Page 4 main MS
	24c	Describe and explain any amendments to information provided at registration or in the protocol.	Page 4 main MS
**Support**	25	Describe sources of financial or non-financial support for the review, and the role of the funders or sponsors in the review.	Page 12 Title page
**Competing interests**	26	Declare any competing interests of review authors.	Page 12 Title page
**Availability of data, code and other materials**	27	Report which of the following are publicly available and where they can be found: template data collection forms; data extracted from included studies; data used for all analyses; analytic code; any other materials used in the review.	Page 12 Title page

PRIMSA Abstract Checklist

Topic	No.	Item	Reported?
**TITLE**			
**Title**	1	Identify the report as a systematic review.	Yes
**BACKGROUND**			
**Objectives**	2	Provide an explicit statement of the main objective(s) or question(s) the review addresses.	Yes
**METHODS**			
**Eligibility criteria**	3	Specify the inclusion and exclusion criteria for the review.	Yes
**Information sources**	4	Specify the information sources (e.g. databases, registers) used to identify studies and the date when each was last searched.	Yes
**Risk of bias**	5	Specify the methods used to assess risk of bias in the included studies.	Yes
**Synthesis of results**	6	Specify the methods used to present and synthesize results.	Yes
**RESULTS**			
**Included studies**	7	Give the total number of included studies and participants and summarise relevant characteristics of studies.	Yes
**Synthesis of results**	8	Present results for main outcomes, preferably indicating the number of included studies and participants for each. If meta-analysis was done, report the summary estimate and confidence/credible interval. If comparing groups, indicate the direction of the effect (i.e. which group is favoured).	Yes
**DISCUSSION**			
**Limitations of evidence**	9	Provide a brief summary of the limitations of the evidence included in the review (e.g. study risk of bias, inconsistency and imprecision).	Yes
**Interpretation**	10	Provide a general interpretation of the results and important implications.	Yes
**OTHER**			
**Funding**	11	Specify the primary source of funding for the review.	Yes
**Registration**	12	Provide the register name and registration number.	Yes

From: Page MJ, McKenzie JE, Bossuyt PM, Boutron I, Hoffmann TC, Mulrow CD, et al.
MS: manuscript.

Table S2. Risk of bias assessment according to ROB-2.

Study(published)	Randomization process	Deviations from intended interventions	Missing outcome data	Measurement of the outcome	Selection of the reported result	Overall Bias
Grønberg et al., 2016	Low	Some concerns	Low	Low	Low	Some concerns
Qiu et al., 2021	Low	Low	Low	Low	Low	Low
Andrew et al., 1999	Low	Low	Low	Low	Low	Low
Bonner et al., 1999	Low	Low	Low	Low	Low	Low
Faivre-Finn et al., 2017	Low	Low	Low	Low	Low	Low
Bogart et al., 2021	Low	Low	Low	Low	Low	Low
Blackstock et al., 2005	Some concerns	Low	Low	Low	High	High

Table S3. Risk of bias assessment according to NOS.

Study (published)	Representativeness of cohort	Selection of the non-exposed cohort	Ascertainment of exposure	Outcome was not present at the start of study	Comparability of cohorts	Assessment of outcome	Length of follow-up	Adequacy of follow-up	Quality score (max 9)
Bettington et al., 2012	1	1	1	1	0	1	0	1	6
Yan et al., 2021	1	1	1	1	2	1	1	1	9
Gazula et al., 2014	1	1	1	1	0	1	1	1	7
Han et al., 2015	1	1	1	1	1	1	1	1	8
Tan et al., 2021	0	1	1	1	1	1	1	1	6
Tomita et al., 2010	1	0	1	1	1	1	1	1	7
Socha et al., 2015	1	0	1	1	0	1	1	1	6
Zayed et al., 2020	1	1	1	1	0	1	1	1	8
Zhang et al., 2017	1	1	1	1	1	1	1	1	8

Table S4. Risk of bias assessment according to MINORS criteria.

Study (published)	clearly stated aim	Inclusion of consecutive patients	Prospective collection of data	Appropriate endpoints	Unbiased assessment of endpoints	Appropriate follow-up period	Loss to follow up less than 5%	Prospective calculation of the study size	Quality score (max 16)
Xia et al., 2015	2	2	2	1	1	2	2	2	14
Baas et al., 2006	2	2	2	1	1	2	2	2	14
Bogart et al., 2004	2	2	2	2	1	2	2	2	15
Elias et al., 1999	2	2	2	1	1	2	2	1	13
Hanna et al., 2002	2	2	2	1	1	2	2	2	14
Kakolyris et al., 2006	2	2	2	2	1	0	2	2	13
Kelley et al., 2013	2	2	2	1	2	2	2	2	15
Le et al., 2009	2	2	2	0	1	2	2	2	13
Miller et al., 2007	2	2	2	1	2	2	2	2	15
Sohn et al., 2007	2	2	2	1	1	2	2	2	14
Wahba et al., 2012	2	2	2	1	1	2	2	0	12
Xenidis et al., 2010	2	2	2	2	1	2	2	2	15
Chen et al., 2005	2	2	2	2	1	2	2	1	14
Ettinger et al., 2005	2	2	2	1	1	2	2	2	14

Han et al., 2005	2	2	2	1	1	2	2	2	14
Hügli et al., 2000	2	2	2	1	1	2	2	1	13
Johnson et al., 1996	2	2	2	1	1	2	2	2	14
Mennecier et al., 2000	2	2	2	1	1	2	2	0	12
Saito et al., 2006	2	2	2	2	1	2	2	2	15
Schild et al., 2007	2	2	2	1	1	2	2	2	14
Sorensen et al., 2008	2	2	2	2	1	2	2	2	15
van Loon et al., 2010	2	2	2	2	1	2	2	2	15

Table S5. Characteristics of each enrolled study.

Study（published)	Inclusion period	Study design	Country	Group	Sample size(n)	Patient characteristics	Total dose (Gy)	Total fractions	Gy/fraction	Total radiation time(days)	CorrectedBED$_{10}$ (Gy)	TRT technique	Interventions	PCI completion (%)	Median follow-up(months)	Survival
**Randomized controlled trials**																
Grønberg et al., 2016[8]	2005-2011	Phase II	Norway	HypoTRT	84	Median age(years): 63(40-85); Sex: male(54%), female(46%); Stage: I(8%), II-III(85%), unknown(7%)	42	15	2.8	19	45.92	3D-CRT	HypoTRT delivered with the second and the third cycle of EP (a total of 4 cycles)	NA	59	Median OS: 18.8months; 2-yr OS: 42%; 4-yr OS: 25%
				HyperTRT	73	Median age(years): 63(44-79); Sex: male(49%), female(51%); Stage: I(8%), II-III(80%), unknown(12%)	45	30	1.5	19	43.91		HyperTRT delivered with the second and the third cycle of EP (a total of 4 cycles)			Median OS: 25.1months; 2-yr OS: 53%; 4-yr O: 25%
Qiu et al., 2021[9]	2015-2019	Phase II	China	HypoTRT	88	Median age(years): 58(35-75); Sex: male(84%), female(16%); Stage: I(1%), II-III(99%)	65	26	2.5	36	66.40	IMRT	HypoTRT concurrent with EP, 81% patients received HypoTRT starting with the third cycle of EP (a total of 4-6 cycles)	NA	24.3	Median OS: 39.3months; 2-yr OS: 74%
				HyperTRT	94	Median age(years): 58(19-75); Sex: male(88%), female(12%); Stage: I(2%), II-III(88%)	45	30	1.5	19	43.91		HypoTRT concurrent with EP, 80% patients received HypoTRT starting with the third cycle of EP (a total of 4-6 cycles)			Median OS: 33.6months; 2-yr OS: 70%
Turrisi et al., 1999[10]	1989-1992	Phase III	USA	ConvTRT	206	Median age(years): 63(34-80); Sex: male(59%), female(41%)	45	25	1.8	33	39.49	2D-RT	ConvTRT concurrent with the first cycle of EP(a total of 4 cycles)	NA	96	Median OS: 19months; 2y-OS: 41%;5y-OS:16%
				HyperTRT	211	Median age (years): 61 (30-82);Sex: male(58%), female(42%)	45	30	1.5	19	43.91		HyperTRT concurrent with the first cycle of EP(a total of 4 cycles)			Median OS: 23months; 2y-OS: 47%;5y-OS:26%
Bonner et al., 1999[11]	1990-1996	Phase III	USA	ConvTRT	132	Age (years): <60(35%); ≥60(65%);Sex: male(58%), female(42%)	50.4	28	1.8	38	43.80	2D-RT	ConvTRT concurrent with the forth cycle of EP(a total of 6 cycles)	NA	39	2y-OS: 47%;3y-OS:34%
				HyperTRT	130	Age (years): <60(35%); ≥60(65%);Sex: male(57%), female(43%)	48	32	1.5	38	39.53		HyperTRT(with a 2.5-week break after the initial 24Gy) concurrent with the forth cycle of EP(a total of 6 cycles)			2y-OS: 45%;3y-OS:29%

Faivre-Finn et al., 2017[12]	2008-2013	Phase III	UK	ConvTRT	270	Median age(years): 63(34-81); Sex: male(55%), female(45%); stage: I(8%), II-III(92%), unknow(6%)	66	33	2	45	60.64	3DCRT, IMRT	ConvTRT concurrent with the second cycle of EP (a total of 6 cycles)	85	45	Median OS: 25months; 2-yrOS: 51%; 5-yrOS: 31%
				HyperTRT	273	Median age(years): 62(29-84); Sex: male(54%), female(46%); Stage: I(<1%), II-III(92%), unknow(7%)	45	30	1.5	19	43.91		HyperTRT concurrent with the second cycle of EP (a total of 6 cycles)	81		Median OS: 30months; 2-yr OS: 56%; 5- yr OS: 34%
Bogart et al., 2021[13]	2008-2019	Phase III	USA	ConvTRT	325	Median age(years): 63(37-80); Sex: male(48%), female(52%);	70	35	2	47	64.61	3D-CRT, IMRT	ConvTRT concurrent with the first or the second cycle of EP or EC(a total of 4 cycles)	NA	48	Median OS: 30.5months; 2-yr OS: 56%; 5-yr OS: 34%
				HyperTRT	313	Median age(years): 64(42-81); Sex: male(49%), female(51%);	45	30	1.5	19	43.91		HyperTRT concurrent with the first or the second cycle of EP or EC(a total of 4 cycles)			Median OS: 28.7months; 2-yr OS: 59%; 5-yr OS: 29%
Blackstock et al., 2005[14]	1987-1992	Phase III	USA	ConvTRT	56	Median age(years): 63(44-78); Sex: male(48%), female(52%)	50	25	2	33	46.39	2D-RT	ConvTRT concurrent with the first 2 cycles and adjuvant 4 cycles of EP or CAV	60	14.7	Median OS: 15months; 2-yr OS: 31%; 5-yr OS: 17%
				HypoTRT	54	Median age(years): 60(41-75); Sex: male(74%), female(26%)	50	20	2.5	46	43.53		HypoTRT delivered via an interdigitating split course in 2.5 Gy fractions (8 weeks) concurrent with the first 3 cycles of EP or CAV	51		Median OS: 14months; 2-yr OS: 36%; 5-yr OS: 18%
**Observational studies**																
Bettington et al., 2012[15]	2000-2009	Retrospective	Australia	HypoTRT	38	Median age(years): 67(42-80); Sex: male(50%), female(50%)	40	15	2.67	19	42.84	3D-CRT	HypoTRT concurrent with EP or EC，24% patients received TRT starting within the 2 cycles of induction chemotherapy	NA	NA	Median OS: 21months; 5-yr OS: 20%
				HyperTRT	41	Median age(years): 61(37-74); Sex: male(46%), female(54%)	45	30	1.5	19	43.91		HypoTRT concurrent with EP or EC，51% patients received TRT starting within the 2 cycles of induction chemotherapy			Median OS: 26months; 5-yr OS: 25%
Yan et al., 2021[16]	2007-2019	Retrospective	Canada	HypoTRT	63	Median age(years): 69; Sex: male(54%), female(46%); Stage: I(22%), II-III(78%)	40	15	2.67	19	42.84	3D-CRT	79% patients received concurrent CCRT(a total of 4-6 cycles), mean SER was 65.38days	NA	20.4	5-yr OS: 29%
				HyperTRT	110	Median age(years): 66; Sex: male(58%), female(42%); Stage: I(6%), II-III(94%)	45	30	1.5	19	43.91		95% patients received concurrent CCRT(a total of 4-6 cycles), mean SER was 65.38days			5-yr OS: 26%

Gazula et al., 2014[17]	2005-2010	Retrospective	USA	ConvTRT	20	Median age(years): 65(49-87); Sex (total cohort): male(41%), female(59%); Stage: II-III(100%)	Median 61.2 (50-66.6)	25-37	1.8-2	33-51	46.39-57.55	3D-CRT, IMRT	75% patients received ConvTRT concurrent with EP or EC, 53% HyperTRT concurrent with first or the second cycle of chemotherapy	54	30	Median OS: 1.92 years; 5-yr OS: 32%
				HyperTRT	26	Median age(years): 59(44-73); Stage: I(4%), II-III(96%)	45	30	1.5	19	43.91		96% patients received HyperTRT concurrent with EP or EC, 81% HyperTRT concurrent with first or the second cycle of chemotherapy		33.6	Median OS: 1.98 years; 5-yr OS: 28%
Han et al., 2015[18]	2008-2013	Retrospective	China	ConvTRT	80	Median age(years): 55(35-74); Sex: male(71%), female(29%); Stage: I(5%), II-III(95%)	60	30	2	40	55.50	3D-CRT	89% patients received ConvTRT concurrent with EP or EC or IP or IC, 57.5% TRT concurrent with the first or the second cycle of chemotherapy	50	27.1	Median OS: 29.5months; 2-yr OS: 43%; 5- yr OS: 13%
				HyperTRT	63	Median age(years): 58(45-71); Sex: male(71%), female(29%); Stage: I(6%), II-III(94%)	45	30	1.5	19	43.91		89% patients received ConvTRT concurrent with EP or EC or IP or IC, 66.7% TRT concurrent with the first or the second cycle of chemotherapy	58.7		Median OS: 31.4months; 2-yr OS: 49%; 5- yr OS: 20%
Tan et al., 2021[19]	NA-2020	Retrospective	China	ConvTRT	74	Age(years): 55±10; Sex: male(70%), female(30%)	56	28	2	38	51.53	IMRT	ConvTRT concurrent with 2 cycle of EP(a total of 4 cycles)	NA	29.6	Median OS: 20.2months; 2-yr OS: 39%; 3- yr OS: 15%
				HyperTRT	74	Age(years): 54±10; Sex: male(64%), female(36%)	50	34	1.5	23	48.01		HyperTRT concurrent with 2 cycle of EP(a total of 4 cycles)			Median OS: 23.6months; 2-yr OS: 47%; 3- yr OS: 22%
Tomita et al., 2010[20]	1997-2007	Retrospective	Japan	ConvTRT	61	Median age(years): 66(29-81); Sex: male(82%), female(18%); Stage: I(10%), II-III(90%)	Median 56(54-63)	27-35	1.8-2.0	43(36-59)	49.46	2D-RT (83%)	ConvTRT concurrent with at least 1 cycle of EP or EC, and IP was performed sequentially with ConvTRT(a total of 4 cycles of chemotherapy)	30	22	Median OS: 41months; 3-yr OS: 53%
					29	Median age(years): 71(51-82); Sex: male(86%), female(14%); Stage: I(3%), II-III(97%)	Median 50(39.6-52.2)	20-29	1.8-2.0	41(30-56)	43.09			21	67	Median OS: 14months; 3-yr OS: 14%
				HyperTRT	37	Median age(years): 58(40-68); Sex: male(81%), female(19%); Stage: II-III(100%)	45	30	1.5	19	43.91		HyperTRT concurrent with at least 1 cycle of EP or EC, and IP was performed sequentially with HyperTRT(a total of 4 cycles of chemotherapy)	65	34	Median OS: 30months; 3-yr OS: 44%

Socha et al., 2015[21]	1997-2012	Retrospective	Poland	ConvTRT	82	Median age(years): 59(44-78); Sex: male(57%), female(43%)	56	28	2	38	51.53	2D-RT (50%)/3D-CRT	Induction EP or EC(4-6 cycles) and sequential ConvTRT	45	31	Median OS: 18months; 3-yr:19%
				HypoTRT	42(early)	Median age(years): 59(41-81); Sex: male(52%), female(48%)	42	15	2.8	19	45.92	3D-CRT	HypoTRT delivered sequentially before the third cycles of EP or EC	52	31	Median OS: 27months; 3-yr:40%
					58(late)		42	15	2.8	19	45.92	3D-CRT	HypoTRT delivered sequentially after the third cycle of EP or EC			Median OS: 22months; 3-yr:39%
Zayed et al., 2020[22]	2000-2013	Retrospective	Canada	ConvTRT	61	Median age(years): 68.2±7.2; Sex: male(51%), female(49%); Stage(6th edition): I(3%), II-IV(57%), IV(34%), missing(5%)	60/66	30/33	2	40/45	55.5(82%)/60.64(3%)/Other(15%)	3D-CRT, IMRT, VMAT	The median cycle was 5 cycles of chemotherapy, 97% patients received concurrent CCRT	66.7	60	5-yr OS: 24%
				HypoTRT	56	Median age(years): 63.3±9.2; Sex: male(55%), female(45%); Stage(6th edition): I(9%); II-III(57%), IV(23%), missing(11%)	40/45/45	15/15/20	2.67/3/2.25	19/19/26	42.84(38%)/50.66(7%)/44.4(46%)		The median cycle was 6 of chemotherapy, 80%pantients received concurrent CCRT	58.3	162	5-yr OS: 26%
Zhang et al., 2017[23]	2010-2013	Retrospective	China	ConvTRT	101	Median age(years): 57(38-83); Sex: male(87%), female(13%)	Median 60(56-66)	30(28-33)	2	40(38-45)	55.5(51.53-60.64)	3D-CRT, IMRT	4-6 cycles of EP/EC, 50% patients received concurrent CCRT, 34% HypoTRT started with the first to the third cycle of chemotherapy	47.5	30	Median OS: 25.3months; 1-yr OS: 82%; 2-yr OS: 57%
				HypoTRT	69	Median age(years): 59(31-76); Sex: male(83%), female(17%)	55	22	2.5	30	56.38		4-6 cycles of EP/EC, 91% patients received concurrent CCRT, 77% HypoTRT started with the first to the third cycle of chemotherapy	66.7		Median OS: 27.2months; 1-yr OS: 87%; 2-yr OS: 62%
**Prospective non-RCT studies**																
Gregor et al., 1997[24]	1989-1995	Phase III	UK	HypoTRT	170(A)	Median age(years): 61(34-74); Sex: male(66%), female(34%)	50	20	2.5	89	25.79(A)	2D-RT	CDE and HypoTRT split into 4 courses delivered on days 14 to 21 of the second and subsequent chemotherapy courses(a total of 5 cycles)	NA	43	Median OS: 15months; 2-yr OS: 25%; 3-yr OS: 14%
					165(S)	Median age(years): 61(33-75); Sex: male(68%), female(32%)	50	20	2.5	26	51.78(S)		Induction 5 cycles of CDE and sequential HypoTRT			
Jett et al., 1990[25]	1979-1986	Phase III	USA	HypoTRT	231	Age range(years): 37-79; Sex: male(60%), female(40%)	37.5	15	2.5	19	39.04	2D-RT	HypoTRT concurrent with the fourth cycles of CAV or CAVE	100	NA	CAVE arm, median OS: 15.1months; 2-yr OS: 28%; 5-yr OS:

																13%; CAV arm, median OS: 12.4months; 2-yr OS: 19%; 5-yr OS: 10%
Murray et al., 1993[26]	1985-1988	Phase III	Canada	HypoTRT	155(early)	Median age(years): 62; Sex: male(59%), female(41%)	40	15	2.67	19	42.84	2D-RT	CAV alternating with EP(a total of 6 cycles), HypoTRT concurrent with the second cycles of chemotherapy	NA	60	Median OS: 21.2months; 2-yr OS: 40%; 3-yr:30%; 5-yr OS: 20%
					153(late)	Median age(years): 62; Sex: male(65%), female(35%)	40	15	2.67	19	42.84		CAV alternating with EP(a total of 6 cycles), HypoTRT concurrent with the last cycle of chemotherapy			Median OS: 16months; 2-yr OS: 34%; 3-yr OS: 22%; 5-yr OS: 11%
Sculier et al., 2008[27]	1993-2006	Phase III	Belgium	HypoTRT	204	Age rang(years): 32-75; Sex: male(80%), female(20%)	39.9	15	2.66	19	42.68	No clearly description	HypoTRT concurrent with standard EP or concurrent with daily EP	NA	54	Standard arm, median OS: 15.5months; 2-yr OS: 35%; 5-yr OS: 18%; Daily arm, median OS: 17months; 2-yr OS: 38%; 5-yr OS: 21%
Xia et al., 2015[28]	2007-2012	Phase II	China	HypoTRT	59	Median age(years): 58(33-74); Sex: male(86%), female(14%)	55	22	2.5	30	56.38	IMRT	HypoTRT concurrent with the second or the third cycles of EP(a total of 4-6 cycles)	64	19.5	Median OS: 28.5months; 1-yr OS: 81%; 2-yr OS: 58%
Baas et al., 2006[29]	1999-2001	Phase II	Netherlands	ConvTRT	37	Median age(years): 65(46-82); Sex: male(58%), female(42%)	45	25	1.8	33	39.49	3D-CRT	ConvTRT concurrent with second cycle of carboplatin/paclitaxel/etoposide(a total of 4 cycles)	81.1	NA	Median OS: 19.5months; 2-yr OS: 46%; 5-yr OS: 27%
Bogart et al., 2004[30]	1999-2000	Phase II	USA	ConvTRT	63	Median age(years): 60(42-73); Sex: male(54%), female(46%)	70	35	2	47	64.61	2D-RT/3D-CRT(no clear proportion)	Induction paclitaxel/topotecan(2 cycles) and ConvTRT concurrent with the first cycle of EC(3 cycles)	52.4	24.7	Median OS: 22.4months; 2-yr OS: 48%
Bunn et al., 1995[31]	1989-1991	Phase II	USA	ConvTRT	215	Age range(years):38-78; Sex: male(59%), female(41%)	45	25	1.8	33	39.49	2D-RT	ConvTRT concurrent with the first or the second cycle of EP(a total of 6 cycles)	NA	NA	Median OS: 17months for the no-GM-CSF arm and14 months for the GM-CSF arm
Elias et al., 1999[32]	1985-1994	Phase II	USA	ConvTRT	36	Median age(years): 49(25-59); Sex: male(64%), female(36%)	50-56	25-28	2	33-38	46.39-51.53	2D-RT	Induction chemotherapy (generally 4 cycles) and sequential ConvTRT	72.2	61	Median OS: 26months, 2-yr OS:53%; 5-yr OS: 41%

Hanna et al., 2002[33]	1993-1995	Phase II	USA	ConvTRT	53	Median age(years): 63(39-76); Sex: male(57%), female(43%);	45	25	1.8	33	39.49	2D-RT	ConvTRT concurrent with first cycle of etoposide/cisplatin/ifosfamide (a total of 4 cycles)	NA	60	2-yr OS:36%; 5-yr OS: 13%
Kakolyris et al., 2006[34]	2001-2004	Phase II	Greece	ConvTRT	39	Median age(years): 56(41-75); Sex: male(85%), female(15%);	45-56	25-28	1.8-2.0	33-38	39.49-51.53	No clearly description	Induction EP(3 cycles) and sequential ConvTRT and adjuvant paclitaxel/cisplatin(3 cycles)	NA	15	Median OS: 15months, 1-yr OS: 54%; 2-yr OS: 33%
Kelley et al., 2013[35]	2003-2005	Phase II	USA	ConvTRT	75	Median age(years): 61(41-79); Sex: male(56%), female(44%)	70	35	2	47	64.61	2D-RT/3D-CRT(no clear proportion)	Induction IP(2 cycles) and ConvTRT concurrent with EP(3 cycles）	NA	57	Median OS: 18.1months, 1-yr OS: 69%; 2-yr OS: 31%
Le et al., 2009[36]	2003-2006	Phase II	USA	ConvTRT	68	Median age(years): 63(39-86); Sex: male(44%), female(56%);	61	33	45Gy at 1.8Gy /f and 16Gy boost at 2 Gy/f	45	53.74	3D-CRT	ConvTRT concurrent with the first cycle of etoposide/cisplatin/tirapazamine (2 cycles) and adjuvant EP(2 cycles）	NA	35	Median OS: 21months
Maurer et al., 1997[37]	1986-1992	Phase III	Lebanon	ConvTRT	347	Median age range(years): 60-69; Sex: male(65%), female(35%)	50	25	2	33	46.39	2D-RT	Induction CAE(3 cycles) and ConvTRT concurrent with PCE(2 cycles) and adjuvant CAE(3 cycles)	NA	69	Median OS: 21.4months for the warfarin arm and 18.6 months for the no warfarin arm
McClay et al., 2005[38]	1993-1999	Phase III	USA	ConvTRT	307	Median age(years): 62(34-81); Sex: male(56%), female(44%);	50	25	2	33	46.39	No clearly description	Induction EP with or without tamoxifen(3 cycles) and ConvTRT concurrent EP with or without tamoxifen(2 cycles)	42.7	NA	Median OS: 18.4months for the tamoxifen arm and 20.6months for the without tamoxifen arm
Miller et al., 2007[39]	2001-2003	Phase II	USA	ConvTRT	63	Median age(years): 62(38-78); Sex: male(43%), female(57%)	70	35	2	47	64.61	2D-RT/3D-CRT(no clear proportion)	Induction paclitaxel/topotecan/etoposide(2 cycles) and ConvTRT concurrent with EC(3 cycles)	44.4	48	Median OS: 20months
Sohn et al., 2007[40]	2002-2005	Phase II	Korea	ConvTRT	33	Median age(years): 60(36-76); Sex: male(85%), female(15%);	median 54Gy(45-64.8Gy	30	1.8	40	47.22	No clearly description	ConvTRT concurrent with the second cycle of IP(a total of 6 cycles)	36.4	27	Median OS: 26.1months; 1-yr OS: 77%; 2-yr OS: 55%

Sun et al., 2013[41]	2003-2010	Phase III	Korea	ConvTRT	111(early)	Median age(years): 60(41-75); Sex: male(88%), female(12%)	52.5	25	2.1	33	49.91	3D-CRT	ConvTRT concurrent with the first cycle of EP(a total of 4 cycles)	52.5	59.4	Median OS: 24.1months; 2-yr OS: 51%; 5-yr OS: 24%
					108(late)	Median age(years): 61(39-75); Sex: male(89%), female(11%)							ConvTRT concurrent with the third cycle of EP(a total of 4 cycles)			Median OS: 26.8months; 2-yr OS: 56%; 5-yr OS: 24%
Wahba et al., 2012[42]	2005-2008	Phase II	Egypt	ConvTRT	33	Median age(years): 56(41-75); Sex: male(82%), female(18%);	54	30	1.8	40	47.22	No clearly description	Induction IP(2 cycles) and ConvTRT concurrent with weekly cisplatin	66.7	27	Median OS: 25months; 1-yr OS: 83%; 2-yr OS: 55%
Xenidis et al., 2010[43]	2003-2007	Phase II	Greece	ConvTRT	33	Median age(years): 66(47-75); Sex: male(91%), female(9%)	55	28-31	1.8-2.0	38-43	47.16-50.33	No clearly description	Induction EP(1 cycle) and ConvTRT concurrent with weekly EP and adjuvant IP(3 cycles)	12.1	35.7	Median OS: 19months; 1-yr OS: 72%; 2-yr OS: 28%
Chen et al., 2005[44]	1997-2000	Phase II	China	HyperTRT	57	Median age(years): 60(25-70); Sex: male(88%), female(12%)	56	40	1.4	26	53.12	2D-RT	Induction EP(3 cycles) and sequential HyperTRT and adjuvant EP(3 cycles)	NA	28	Median OS: 24 months; 1-yr OS:81%; 2-yr OS: 49%; 3-yr OS: 21%
Ettinger et al., 2005[45]	1996-1998	Phase II	USA	HyperTRT	53	Median age(years): 56(39-76); Sex: male(51%), female(49%); Stage: I(5%), II-III(95%)	45	30	1.5	19	43.91	No clearly description	HyperTRT concurrent with the first cycle of chemotherapy(paclitaxel/etoposide/cisplatin) (a total of 4 cycles)	32.1	24.7	Median OS: 24.7 months; 2-yr OS: 55%
Grønberg et al., 2021[46]	2014-2018	Phase II	Norway	HyperTRT	81	Median age(years): 65(60-72); Sex: male(42%), female(58%); Stage: I(5%), II-III(95%)	45	30	1.5	19	43.91	3D-CRT, IMRT	HyperTRT concurrent with the second cycles of EP or EC (a total of 6 cycles)	82.4	49	Median OS: 22.6 months;
					89	Median age(years): 65(58-71); Sex: male(44%), female(56%); Stage: II-III(95%)	60	40		26	58.28					Median OS: 37.2months;
Han et al., 2008[47]	2003-2006	Phase II	Korea	HyperTRT	76	Median age(years): 63(46-79); Sex: male(86%), female(14%)	45	30	1.5	19	43.91	No clearly description	Induction IP(2 cycles) and HyperTRT concurrent with EP(2 cycles) plus amifostine or epoetin-a	NA	25.3	Median OS: 22.6 months for the amifostine arm and 25.6 months for the epoetin-a arm
Han et al., 2005[48]	2001-2003	Phase II	Korea	HyperTRT	35	Median age(years): 63(38-75); Sex: male(94%), female(6%)	45	30	1.5	19	43.91	3D-CRT	Induction IP(2 cycles) and HyperTRT concurrent with EP(2 cycles)	42.9	26.5	Median OS: 25months; 1-yr OS: 86%; 2-yr OS: 54%
Hu et al., 2020[49]	2002-2017	Prospective	China	HyperTRT	309	Age range(years): 32-75; Sex: male(84%),	45	30	1.5	19	43.91	3D-CRT, IMRT	HyperTRT concurrent with the third cycle of EP(a total of 4-6 cycles). HyperTRT was	63.1	19.6	The study arm, median OS: 21.9 months, 3-yr OS:

						female(16%); Stage: I(1%), II-III(99%)							delivered to the postchemotherapy or prechemotherapy tumor volume.			30%, 5-yr OS: 23%; The control arm: median OS: 26.6 months, 3-yr OS: 37%, 5-yr OS: 28%.
Hügli et al., 2000[50]	1993-1998	Phase II	France	HyperTRT	52	Median age(years): 54(33-67); Sex: male(79%), female(21%)	45	30	1.5	19	43.91	2D-RT	HyperTRT concurrent with the first cycle of EP(a total of 6 cycles)	NA	46	Median OS: 18months; 1-yr OS: 74%; 3-yr OS: 34%; 5-yr OS: 32%
Jeremic et al., 1997[51]	1988-1992	Prospective randomized	Japan	HyperTRT	52(early)	Median age(years): 59(40-67); Sex: male(60%), female(40%)	54	36	1.5	24	52.20	2D-RT	HyperTRT concurrent with the first cycle of EC(2 cycles) and adjuvant EP(4 cycles)	NA	NA	Median OS: 34months; 5-yr OS: 30%
					51 (late)	Median age(years): 59(44-66); Sex: male(61%), female(39%)	54	36	1.5	24	52.20		Induction EP(2cycles) and HyperTRT concurrent EC(2 cycles) and adjuvant EP(2 cycles)			Median OS: 26months; 5-yr OS: 15%
Johnson et al., 1996[52]	1986-1993	Phase II	USA	HyperTRT	53	Median age(years): 59(34-72); Sex: male(69%), female(31%)	45	30	1.5	19	43.91	2D-RT	HyperTRT concurrent with the first cycle of EP(a total of 4 cycles) and adjuvant CAE (4 cycles) or an individualized chemotherapy regimen	35.2	58.8	Median OS: 21months; 1-yr OS: 83%; 2-yr OS: 43%; 5-yr OS: 19%
Kubota et al., 2014[53]	2002-2006	Phase III	Japan	HyperTRT	281	Median age(years): 61(32-70); Sex: male(81%), female(19%)	45	30	1.5	19	43.91	2D-RT/3D-CRT(no clear proportion)	HyperTRT concurrent with the first cycle of EP and adjuvant EP or IP(3 cycles)	60	75.6	Median OS: 2.9 years, 3-yr OS: 48%, 5-yr OS: 34%
Mennecier et al., 2000[54]	1992-1997	Phase II	France	HyperTRT	31	Median age(years): 61(39-81); Sex: male(77%), female(23%)	45	30	1.5	19	43.91	No clearly description	HyperTRT concurrent with the second cycle of EP(a total of 6 cycles)	90.3	16.3	Median OS: 16.3months; 2-yr OS: 25%; 3-yr OS: 19%; 5-yr OS: 14%
Saito et al., 2006[55]	2000-2002	Phase II	Japan	HyperTRT	49	Median age(years): 62(45-70); Sex: male(82%), female(18%); Stage: II-III(100%)	45	30	1.5	19	43.91	No clearly description	HyperTRT concurrent with EP(2 cycles) and adjuvant IP(3 cycles)	NA	29.9	Median OS: 29.9months; 2-yr OS: 49%; 3-yr OS: 30%
Schild et al., 2007[56]	1996-1999	Phase II	USA	HyperTRT	76	Median age(years): 63(38-77); Sex: male(51%), female(49%);	60	40	1.5	40	52.50	No clearly description	HyperTRT concurrent with the fourth cycle of EP(a total of 6 cycles)	80	60	Median OS: 20months; 2-yr OS: 43%; 5-yr OS: 24%
Skarlos et al., 2001[57]	1993-1999	Phase II	Greece	HyperTRT	42(early)	Median age(years): 61(40-76); Sex: male(93%), female(7%)	45	30	1.5	19	43.91	No clearly description	HyperTRT concurrent with the first cycle of EC(a total of 6 cycles)	NA	35	Median OS: 17.5months; 2-yr OS: 36%; 3-yr OS: 22%

					39(late)	Median age(years): 60(37-76); Sex: male(90%), female(10%)	45	30	1.5	19	43.91		HyperTRT concurrent with the fourth cycle of EC(a total of 6 cycles)			Median OS: 17months; 2-yr OS: 29%; 3-yr OS: 13%
Sorensen et al., 2008[58]	2000-2004	Phase II	Denmark	HyperTRT	40	Median age(years): 60(43-75); Sex: male(48%), female(52%);	45	30	1.5	19	43.91	3D-CRT	Induction topotecan/cisplatin(1 cycle) and HyperTRT concurrent with EC(2cycles）and adjuvant topotecan/cisplatin or EC(a total of 3 cycles)	35	NA	Median OS: 22.9months; 5-yr OS: 21%
Takada et al., 2002[59]	1991-1995	Phase III	Japan	HyperTRT	114(concurrent)	Median age(years): 65(39-74); Sex: male(80%), female(20%); Stage: II-III(100%)	45	30	1.5	19	43.91	2D-RT	HyperTRT concurrent with the first cycle of EP(a total of 4 cycles)	NA	NA	Median OS: 27.2months; 2-yr OS: 54%; 3-yr OS: 30%; 5-yr OS: 24%
					114(sequential)	Median age(years): 64(30-74); Sex: male(82%), female(18%); Stage: II-III(100%)	45	30	1.5	19	43.91		Induction EP(4 cycles) and sequential HyperTRT			Median OS: 19.7months; 2-yr OS: 35%; 3-yr OS: 20%; 5-yr OS: 18%
van Loon et al., 2010[60]	2004-2006	Prospective	Netherlands	HyperTRT	60	Median age(years): 66(48-55); Sex: male(67%), female(33%)	45	30	1.5	19	43.91	3D-CRT	HyperTRT concurrent with EP as early as possible (a total of 5 cycles, median SER was 39 days)	83.3	18.5	Median OS: 19months; 2-yr OS: 35%

TRT, thoracic radiotherapy; HypoTRT, hypofractionated thoracic; ConvTRT, conventional fractionated thoracic radiotherapy; HyperTRT, hyperfractionated thoracic radiotherapy; 2D-RT, two-dimensional radiotherapy; 3D-CRT, three-dimensional conformal radiotherapy; IMRT, intensity-modulated radiotherapy; VMAT, volumetric-modulated arc therapy; BED_{10}, biologically effective dose; CCRT, concurrent chemoradiotherapy; PCI, prophylactic cranial irradiation; SER, interval between start of chemotherapy and end of thoracic radiotherapy; OS, overall survival; EP, etoposide/cisplatin; EC, etoposide/carboplatin; CDE, cyclophosphamide/doxorubicin/etoposide; CAV, cyclophosphamide/doxorubicin/vincristine; CAE, cyclophosphamide/doxorubicin/etoposide; CAVE, cyclophosphamide/doxorubicin/vincristine/etoposide; IP, irinotecan/cisplatin; IC, irinotecan/carboplatin; PCE, cisplatin, cyclophosphamide, and etoposide; NA, not applicable.

Table S6. Summary of results from direct comparisons for overall survival.

Study	No. of events/No. of patients		HR	95%CI
	Experimental	Comparator		
Groenberg et al., 2016[8]	HypoTRT	HyperTRT	1.06	0.73-1.52
Qiu et al., 2021[9]	HypoTRT	HypoTRT	0.71	0.46-1.11
				Pooled (pair-wise) I^2=41.2%
				Pooled (network) I^2=5.3%
Turrisi et al., 1999[10]	ConvTRT	HyperTRT	1.2	1.10-1.60
Bonner et al., 1999[11]	HyperTRT	ConvTRT	1.12	0.85-1.52
Faivre-Finn et al., 2017[12]	ConvTRT	HyperTRT	1.18	0.95-1.45
Bogart et al., 2021[13]	ConvTRT	HyperTRT	0.94	0.76-1.20
				Pooled (pair-wise) I^2=44.5%
				Pooled (network) I^2=29.9%
Blackstock et al., 2005[14]	HypoTRT	ConvTRT	1.03	0.70-1.51

HR, hazard ratio; CI, confidence interval; HypoTRT, hypofractionated thoracic radiotherapy; ConvTRT, conventional fractionated thoracic radiotherapy; HyperTRT, hyperfractionated thoracic radiotherapy

Table S7. Network meta-regression results.

RCTs (The value of DICs for model without control is 11.78)

Variable	Estimate	95%CI	SD	DICs
**HyperTRT as the reference group**				
TRT started within 2 cycles of induction chemotherapy				
Yes	0.22	-0.10 to 0.57	0.17	6.83
Corrected BED_{10}	-0.11	-0.43 to 0.20	0.16	10.95
High-dose group	-0.03	-0.37 to 0.30	0.17	12.53
**ConvTRT as the reference group**				
TRT started within 2 cycles of induction chemotherapy				
Yes	-0.14	-0.54 to 0.24	0.20	10.54
Corrected BED_{10}	0.03	-0.33 to 0.38	0.17	12.43
High-dose group	-0.02	-0.35 to 0.31	0.17	12.56

Subgroup of 3D-CRT/IMRT (The value of DICs for model without control is 7.49)

Variable	Estimate	95%CI	SD	DICs
**HyperTRT as the reference group**				
TRT started within 2 cycles of induction chemotherapy				
Yes	0.19	-0.32 to 0.85	0.29	5.81
**ConvTRT as the reference group**				
TRT started within 2 cycles of induction chemotherapy				
Yes	0.01	-0.37 to 6.13	2.38	6.87

All enrolled patients received concurrent chemoradiotherapy.
RCTs, randomized controlled trials; DICs, deviance information criteria; CI, confidence interval; SD, standard deviation; BED_{10}, biologically effective dose; 3D-CRT, three-dimensional conformal; IMRT, intensity modulated radiotherapy.

Table S8. Late severe radiation-related adverse events.

Study	Group	Late Grade 3-5 toxicities	
		RE (n/N(%))	RP(n/N(%))
Qiu et al., 2021[9]	HypoTRT	NA	0/85
	HyperTRT	NA	0/92
Faivre-Finn et al., 2017[12]	ConvTRT	4/233	6/233
	HyperTRT	0/248	6/248
Socha et al., 2015[21]	ConvTRT	0/82	0/82
	HypoTRT	0/100	0/100
Gregor et al., 1997[24]	HypoTRT(alternating)	1/135	53/135
	HypoTRT(sequential)	3/143	53/143
Miller et al., 2007[39]	ConvTRT	5%	5%
Chen et al., 2005[44]	HyperTRT	0/57	3/57
Ettinger et al., 2005[45]	HyperTRT	4/51	0/51
Hu et al., 2020[49]	HyperTRT	NA	2/300
Hügli et al., 2000[50]	HyperTRT	2/52	0/52
Jeremic et al., 1997[51]	HyperTRT	2/103	1/103

RE, radiation esophagitis; RP, radiation pneumonitis; HypoTRT, hypofractionated thoracic radiotherapy; ConvTRT, conventional fractionated thoracic radiotherapy; HyperTRT, hyperfractionated thoracic radiotherapy.

Figure S1. Search strategy. Date of research: 07/31/2021

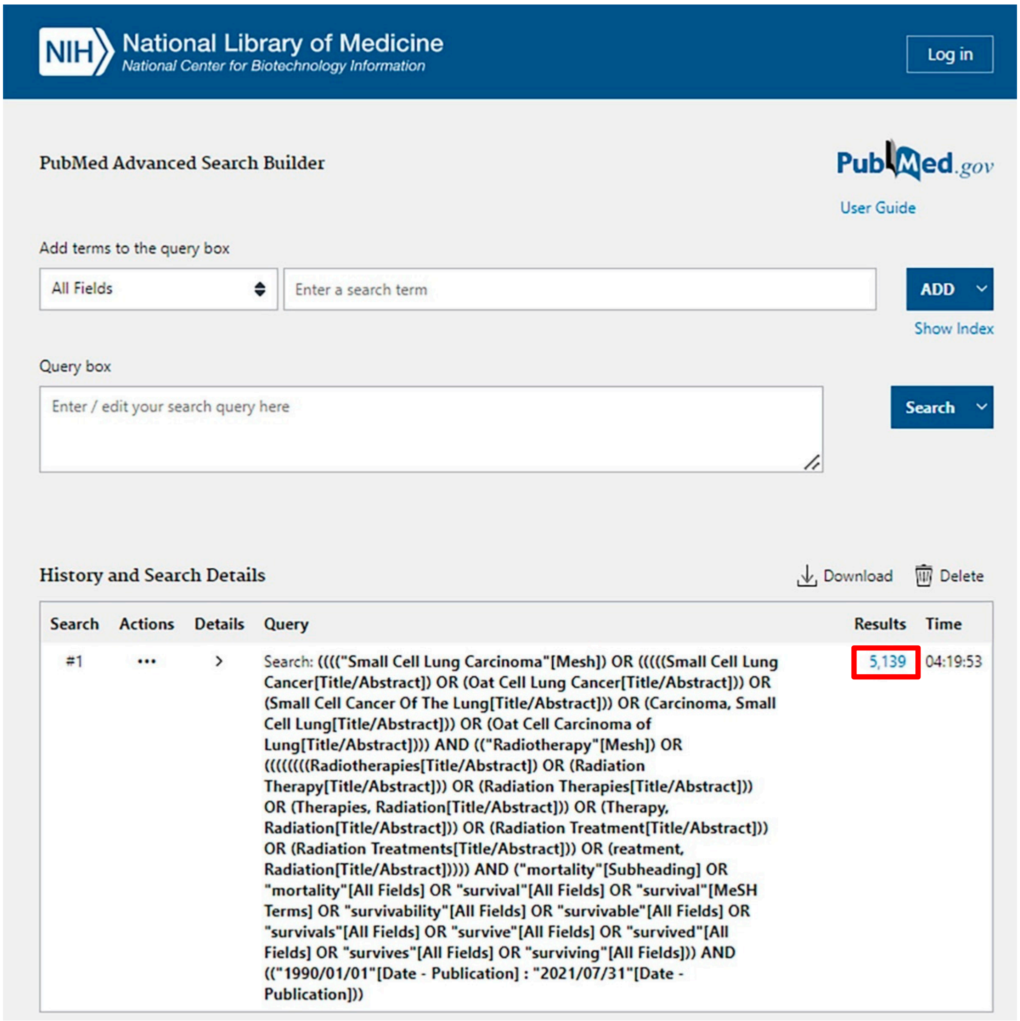

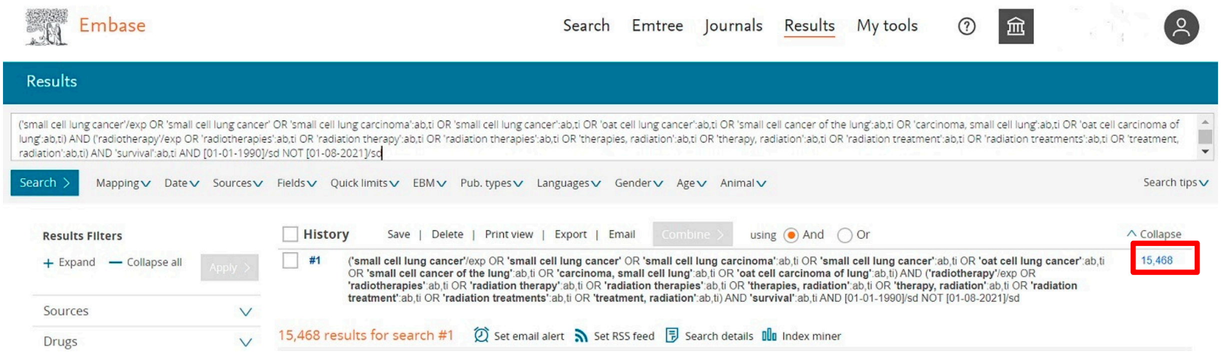

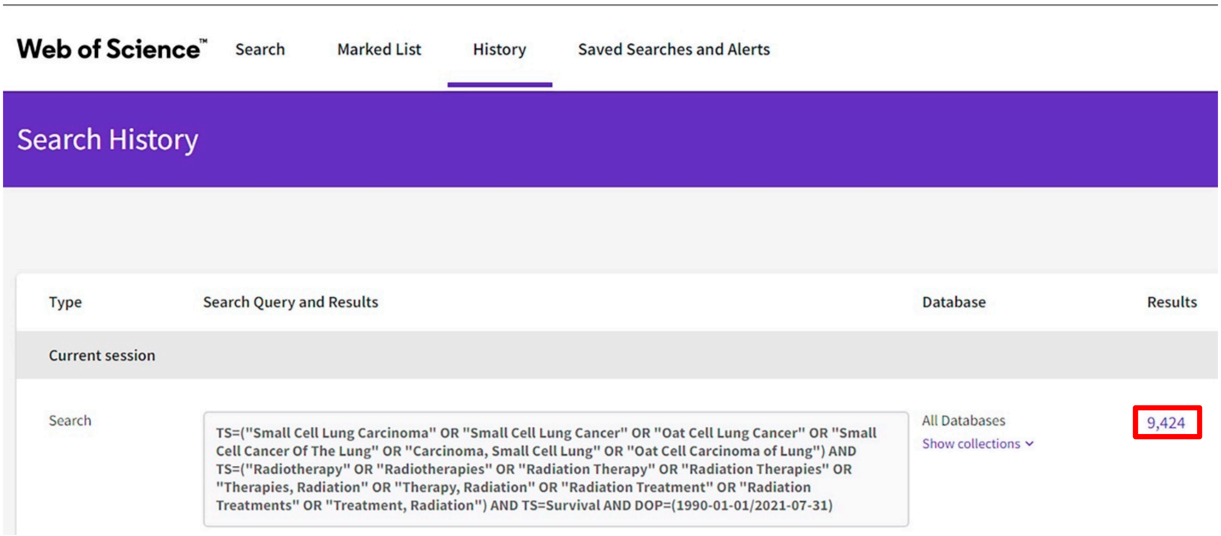
Web of Science™
Search
Marked List
History
Saved Searches and Alerts
Search History
Type
Search Query and Results
Database
Results
Current session
Search
TS=("Small Cell Lung Carcinoma" OR "Small Cell Lung Cancer" OR "Oat Cell Lung Cancer" OR "Small Cell Cancer Of The Lung" OR "Carcinoma, Small Cell Lung" OR "Oat Cell Carcinoma of Lung") AND TS=("Radiotherapy" OR "Radiotherapies" OR "Radiation Therapy" OR "Radiation Therapies" OR "Therapies, Radiation" OR "Therapy, Radiation" OR "Radiation Treatment" OR "Radiation Treatments" OR "Treatment, Radiation") AND TS=Survival AND DOP=(1990-01-01/2021-07-31)
All Databases
Show collections
9,424

Figure S2. Comparisons of reconstructed and original curves.

Original **Reconstructed**

Randomized controlled trials

Grønberg et al., 2016

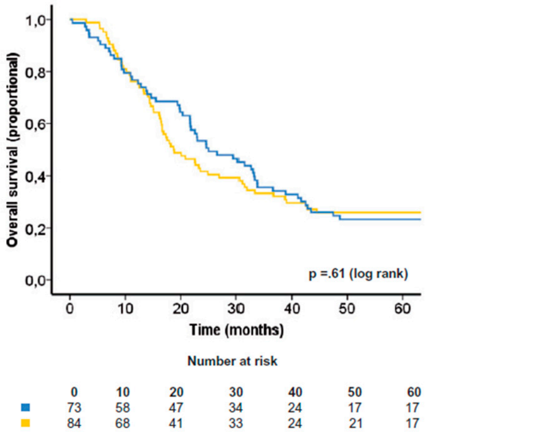

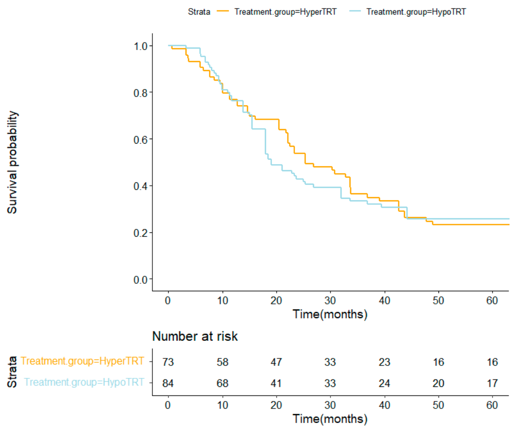

Qiu et al., 2021

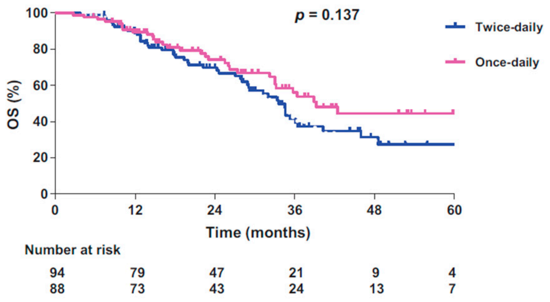

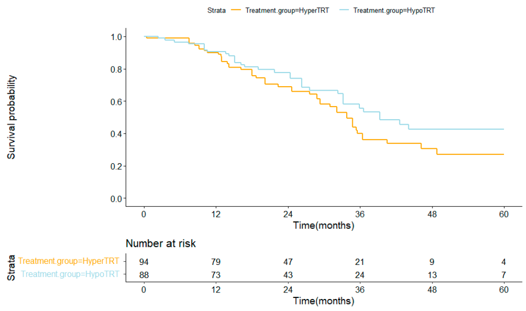

Andrew et al., 1999

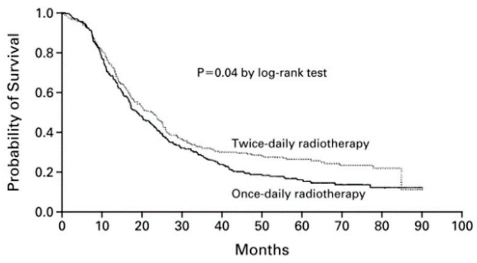

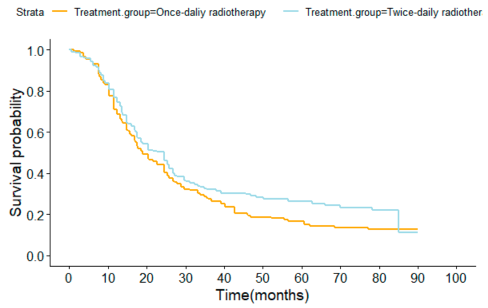

Bonner et al., 1999

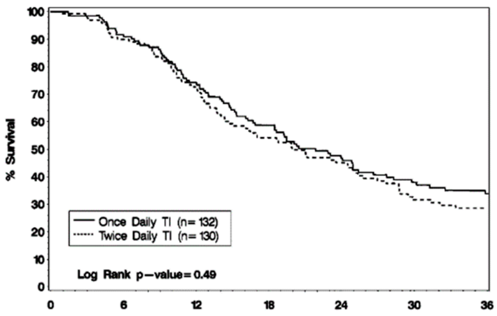

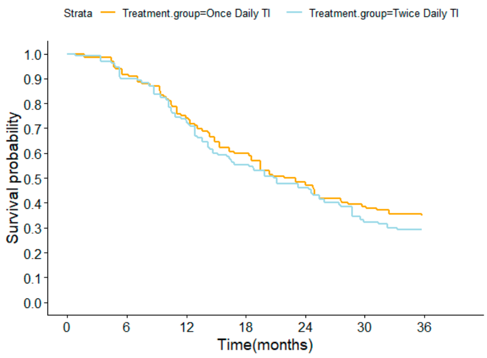

Faivre-Finn et al., 2017

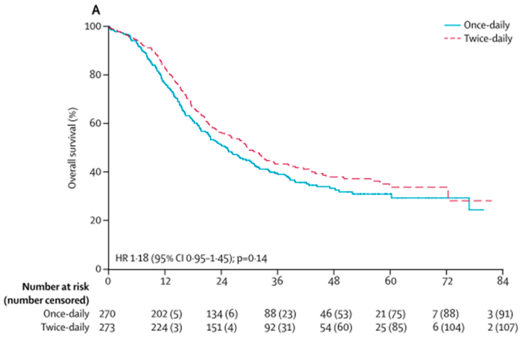

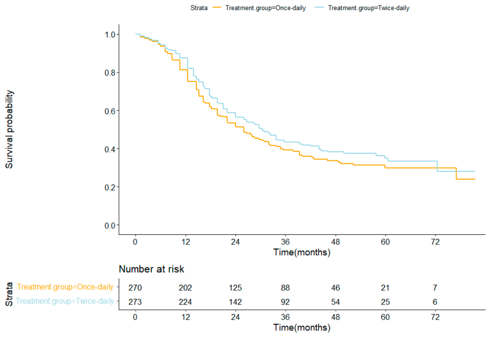

Bogart JA et al., 2021

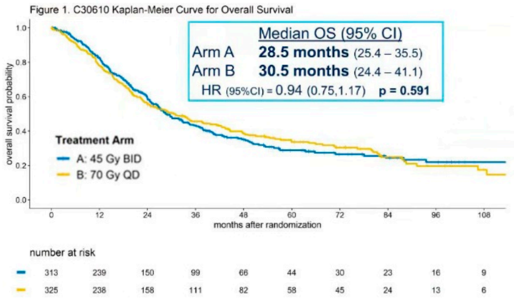

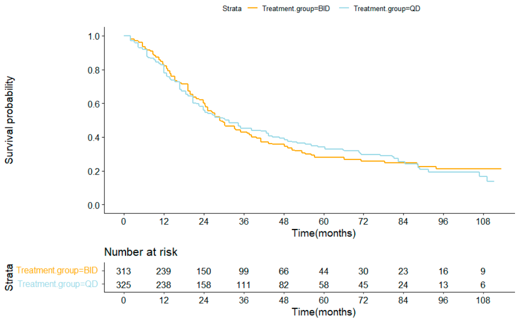

Blackstock et al., 2005

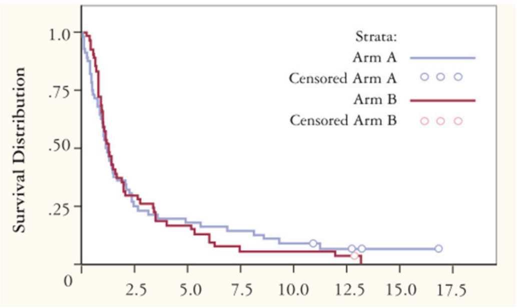

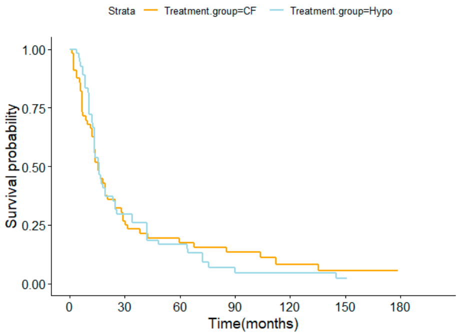

Observational studies

Yan et al., 2021

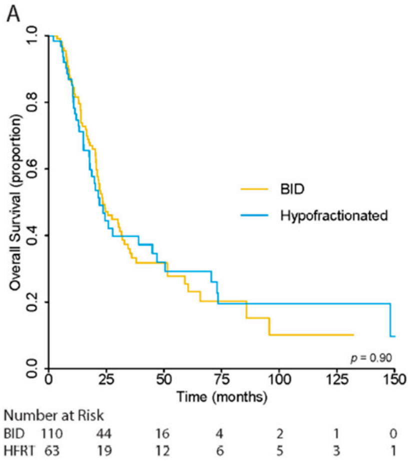

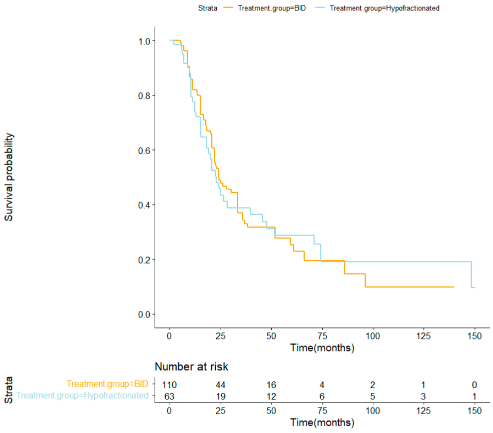

Gazula et al., 2014

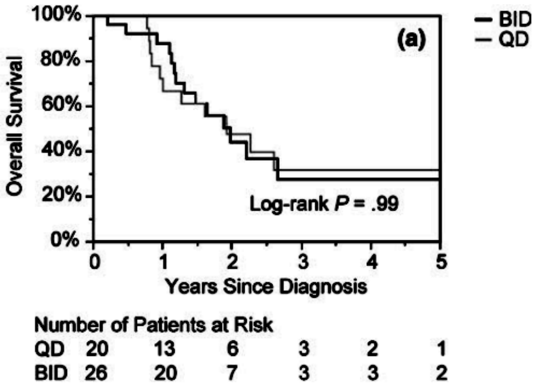

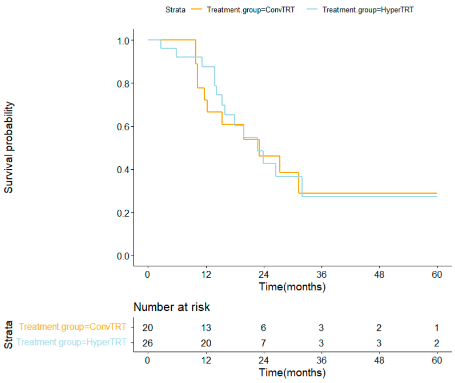

Han et al., 2015

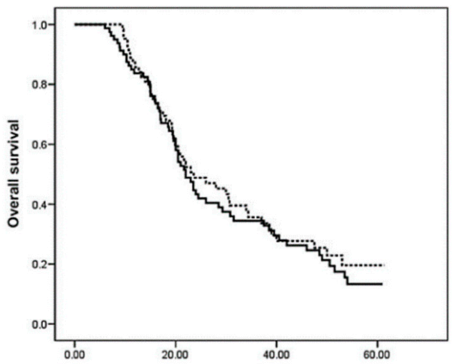

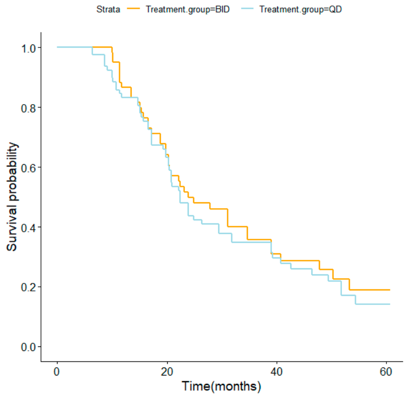

Tan et al., 2021

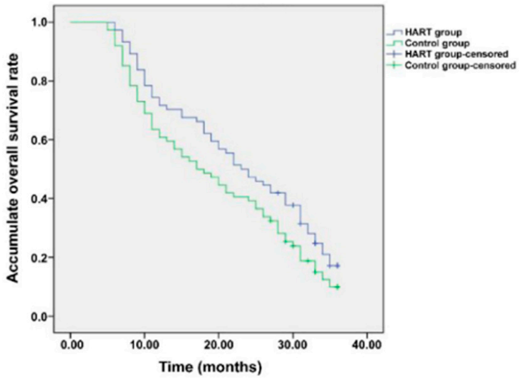

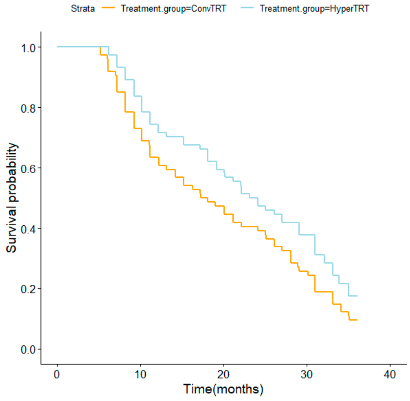

Tomita et al., 2010

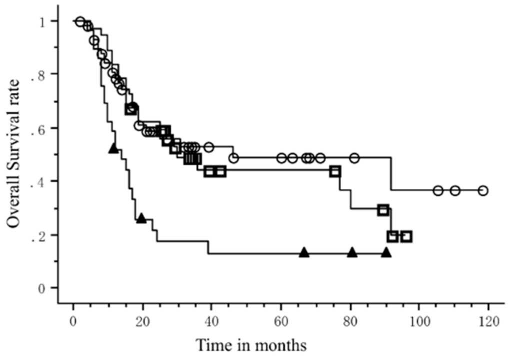

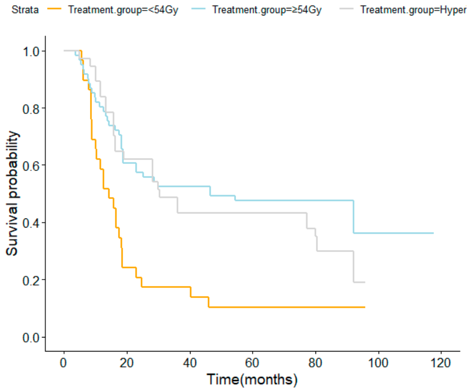

Socha et al., 2015

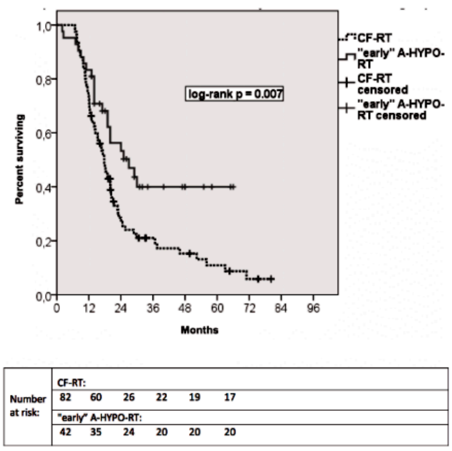

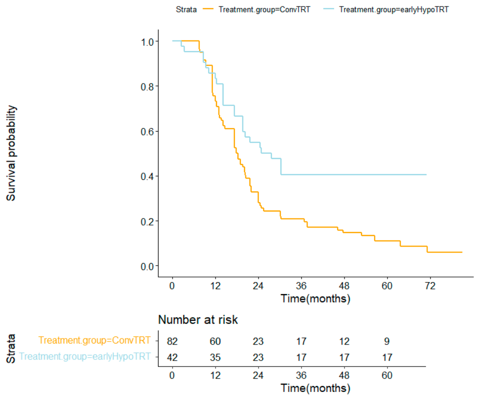

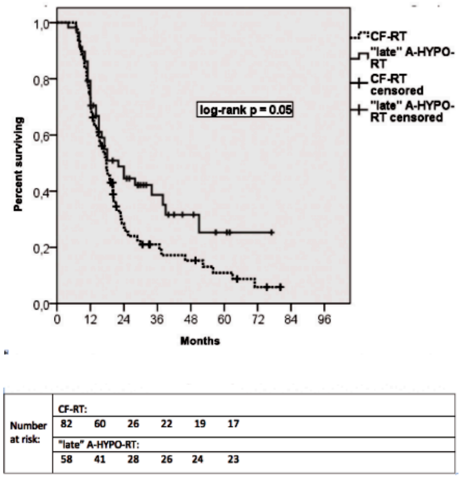

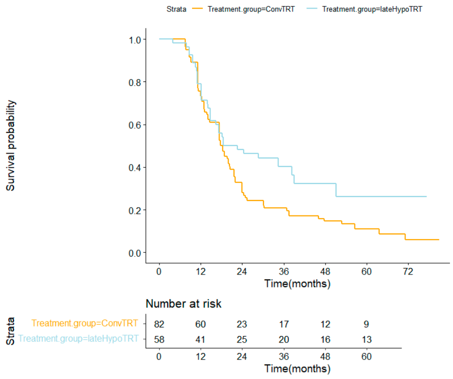

Videtic et al., 2003

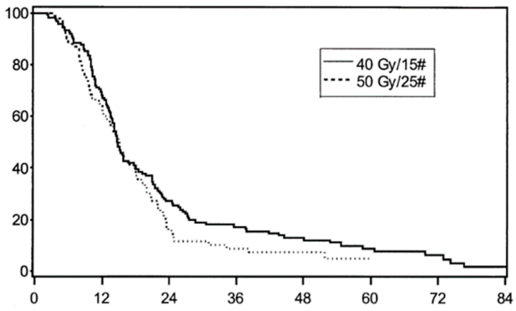

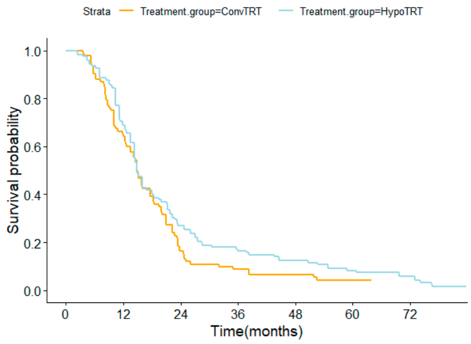

Zayed et al., 2020

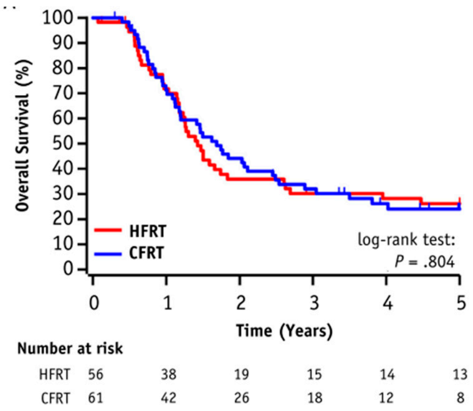

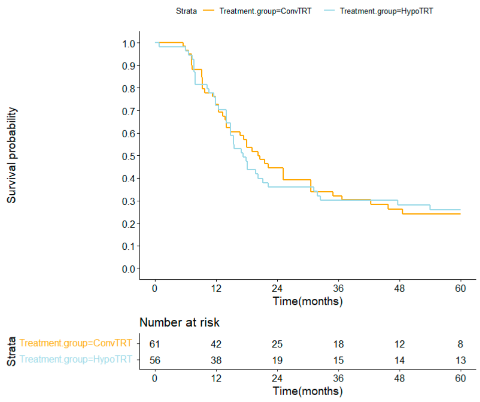

Zhang et al., 2017

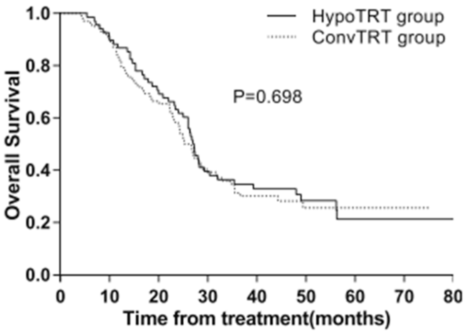

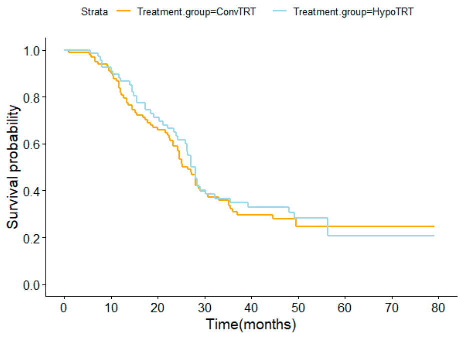

Prospective non-RCT studies

Gregor et al., 1997

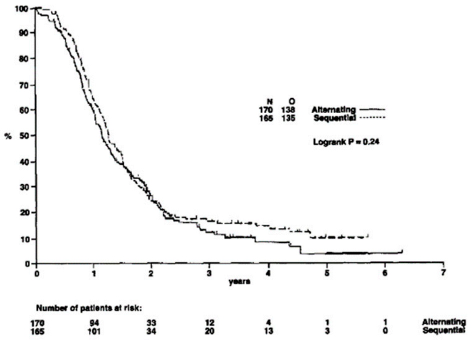

c

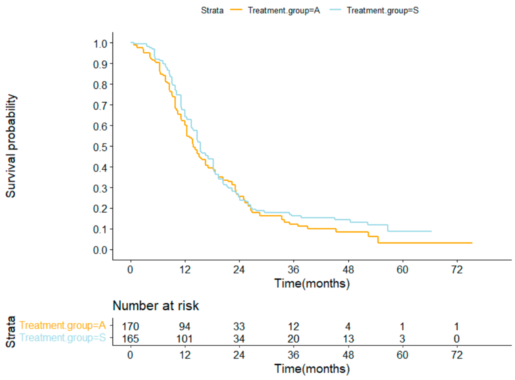

Jett et al., 1990

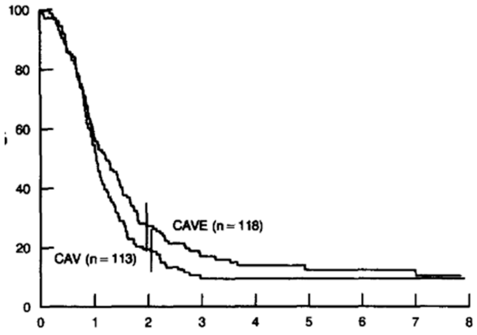

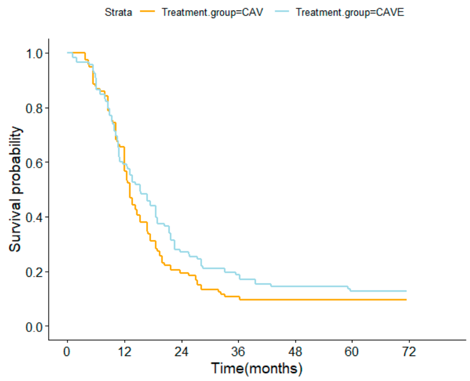

Murray et al., 1993

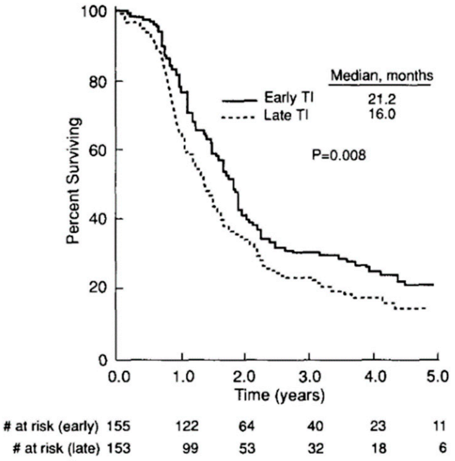

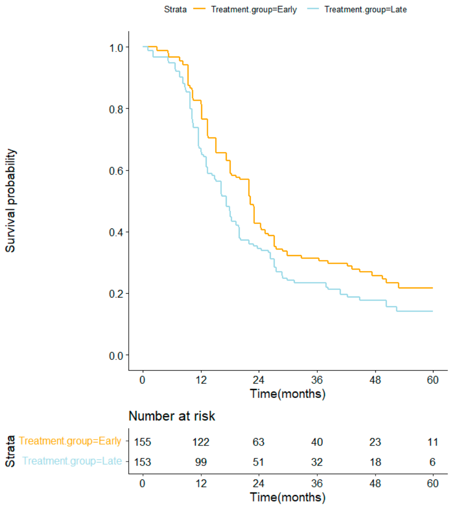

Sculier et al., 2008

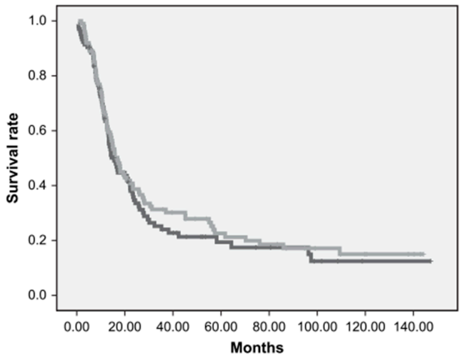

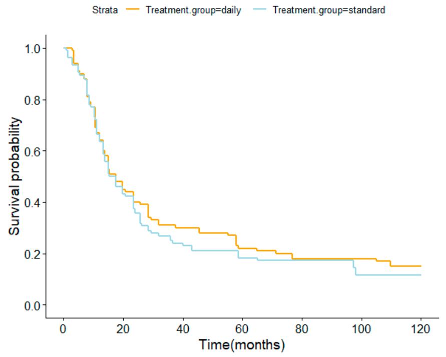

Xia et al., 2015

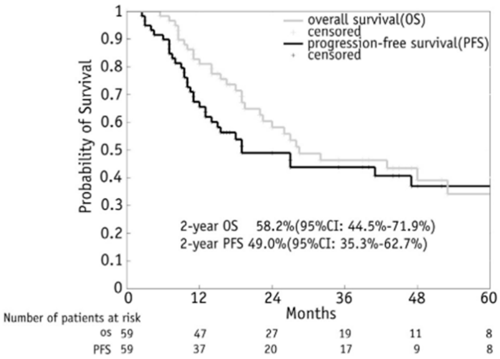

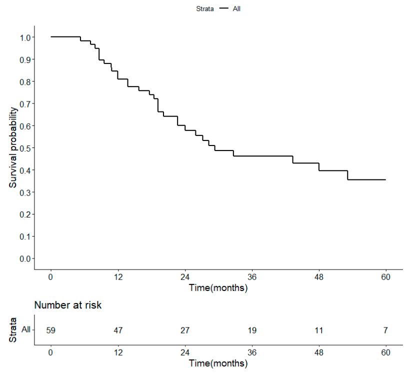

Baas et al., 2006

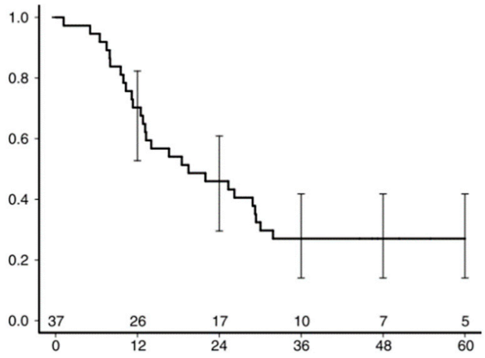

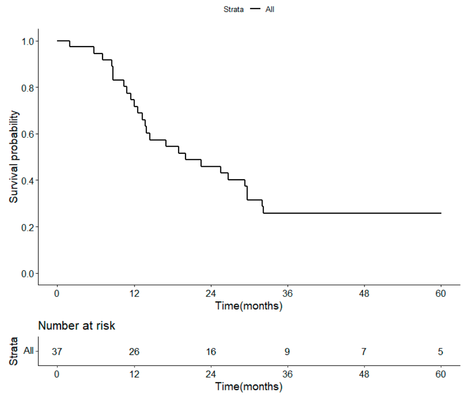

Bogart et al., 2004

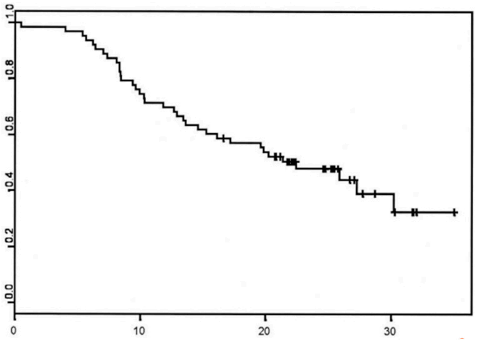

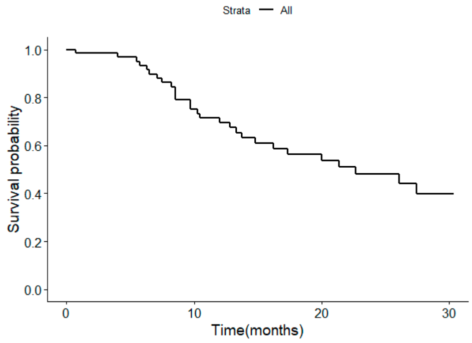

Bunn et al., 1995

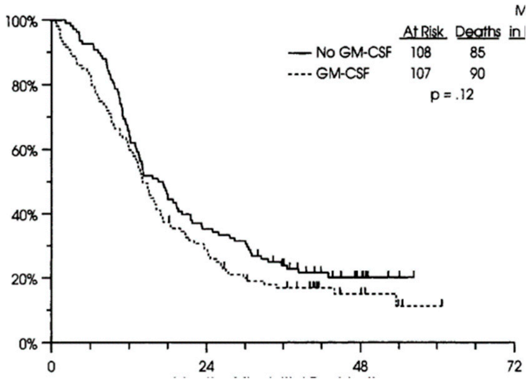

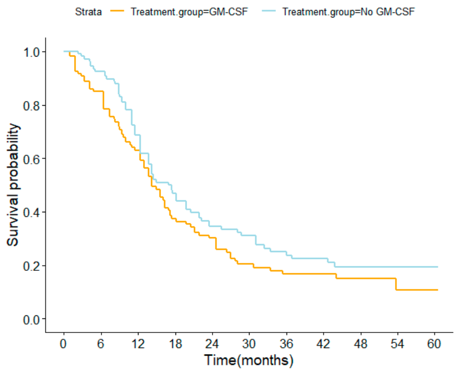

Elias et al., 1999

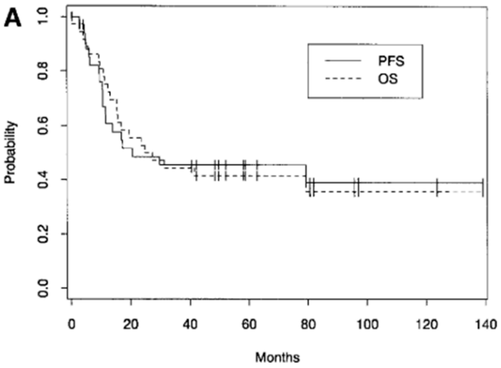

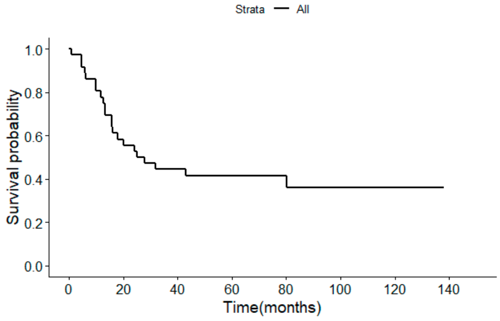

Hanna et al., 2002

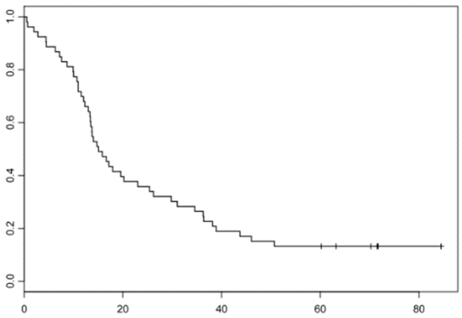

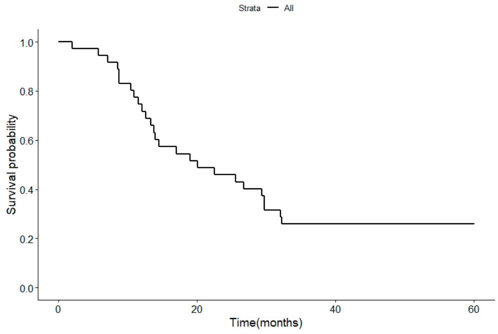

Kakolyris et al., 20c06

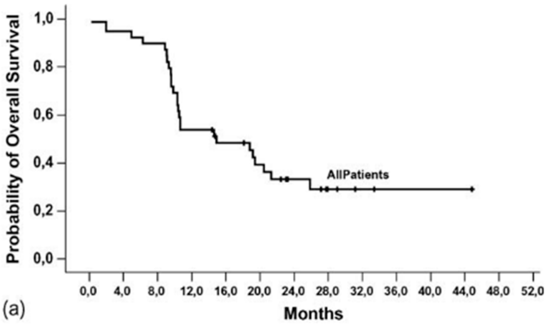

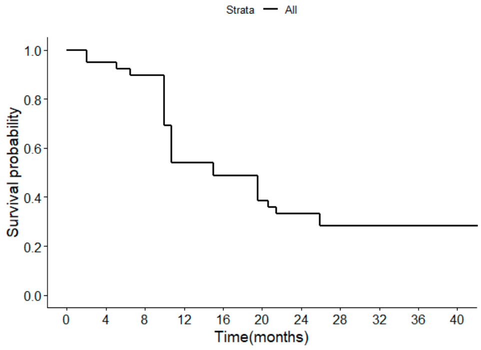

Kelley et al., 2013

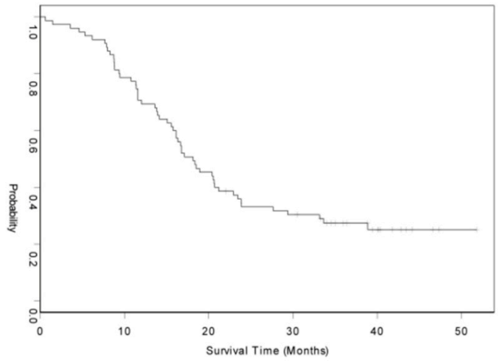

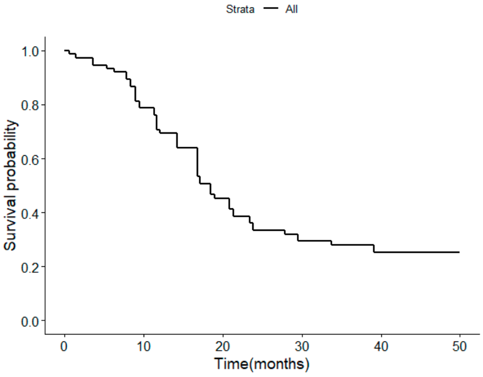

Le et al., 2009

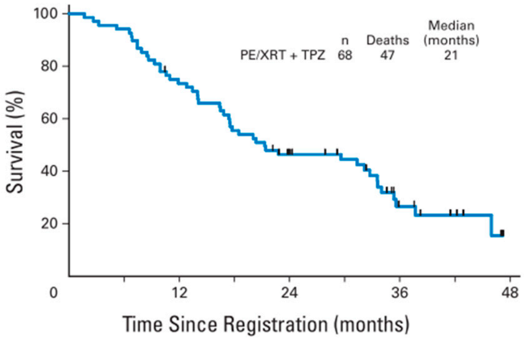

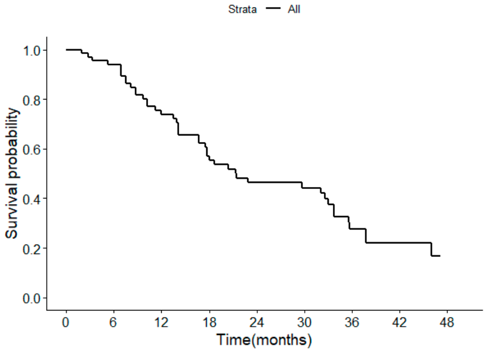

Maurer et al., 1997

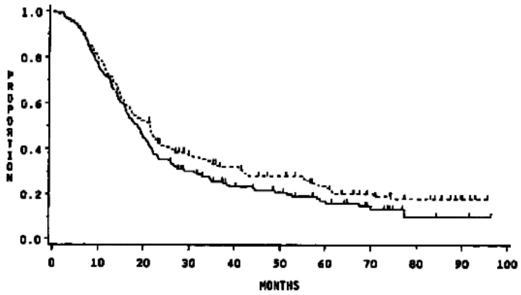

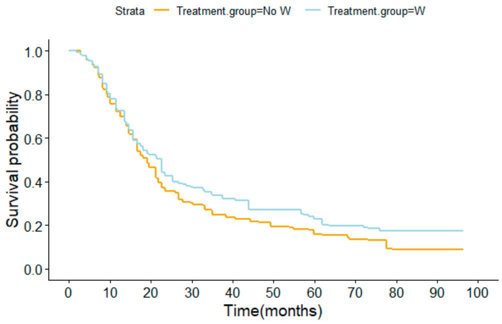

McClay et al., 2005

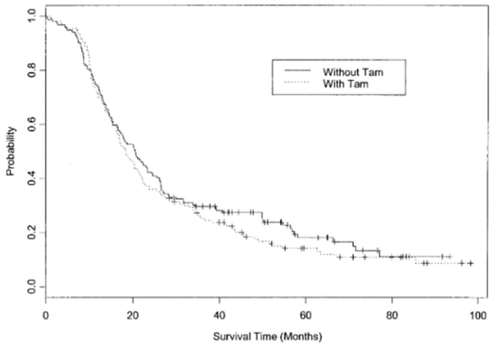

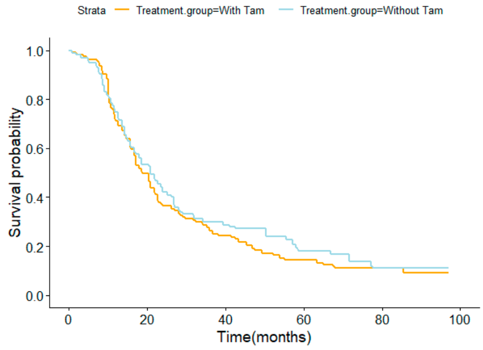

Miller et al., 2007

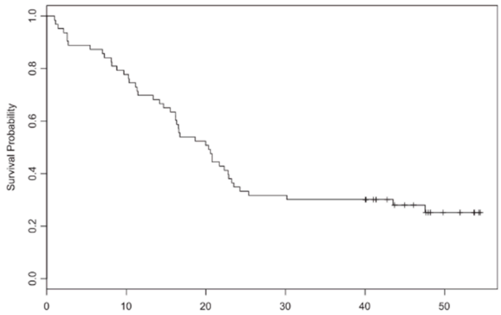

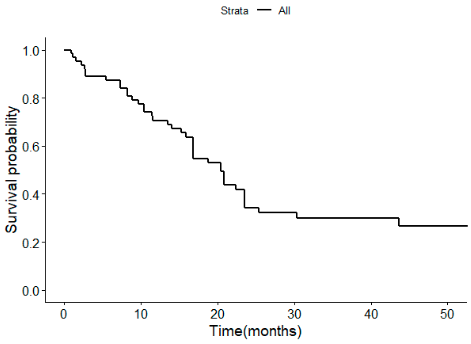

Sohn et al., 2007

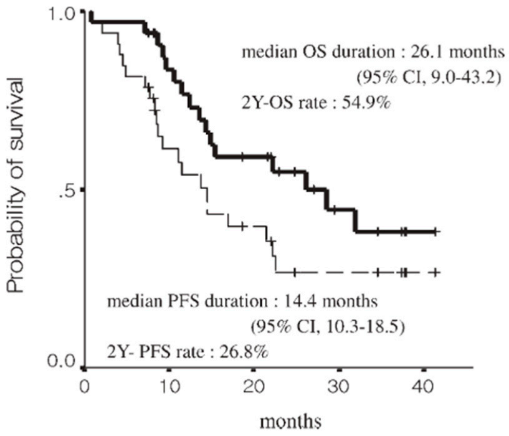

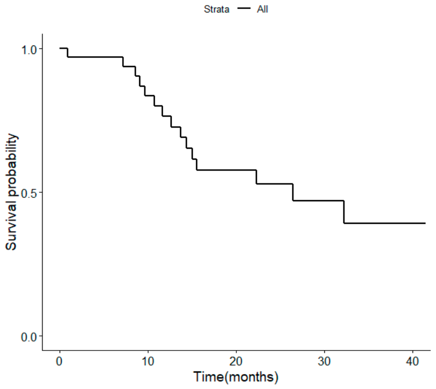

Sun et al., 2013

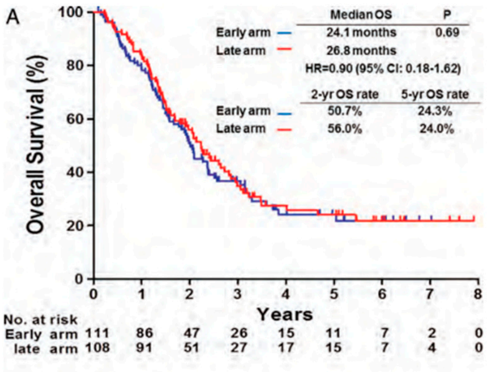

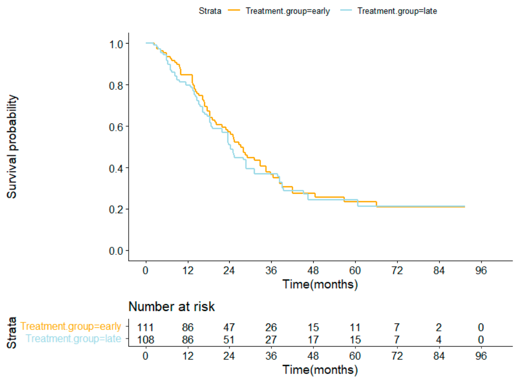

Wahba et al., 2012

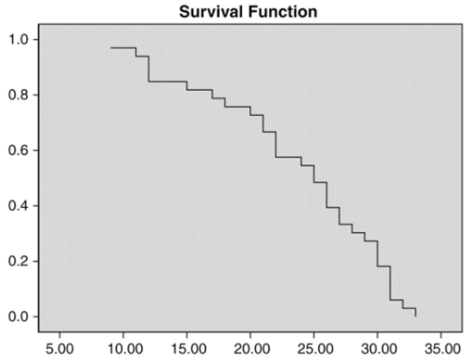

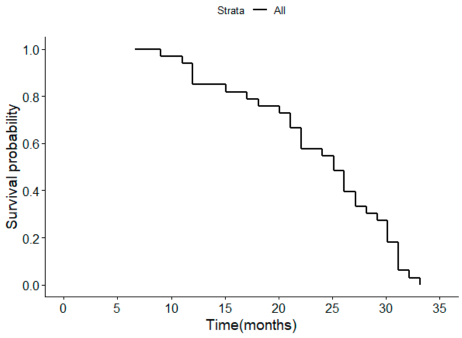

Xenidis et al., 2010

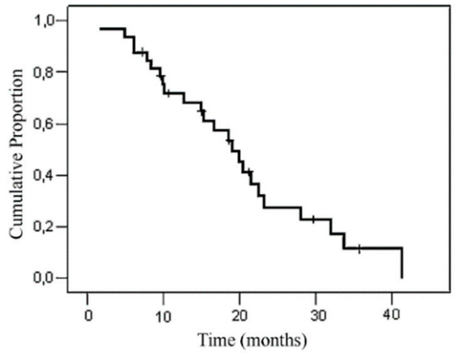

Fig. 2. Kaplan–Meier curve for overall survival in the intent-to-treat population.

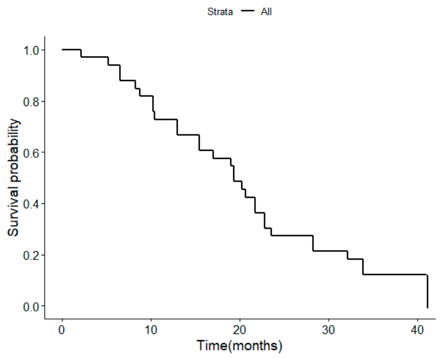

Chen et al., 2005

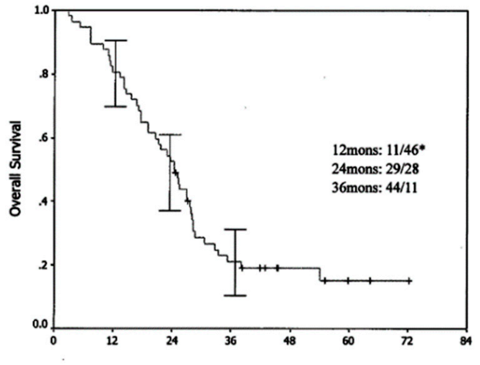

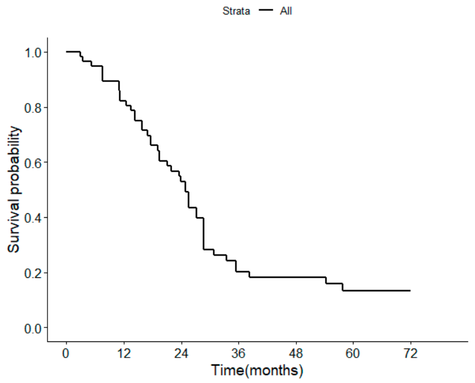

Ettinger et al., 2005

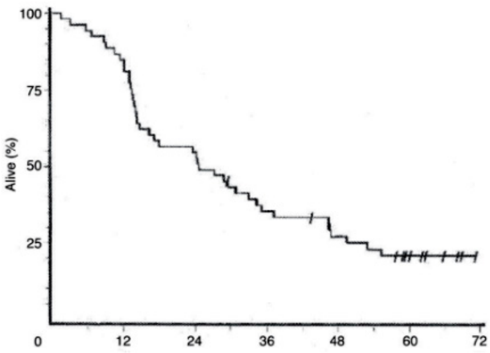

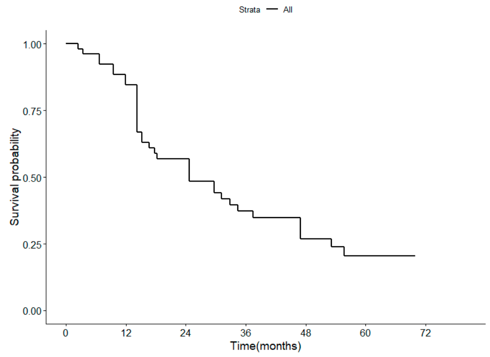

Grønberg et al., 2021

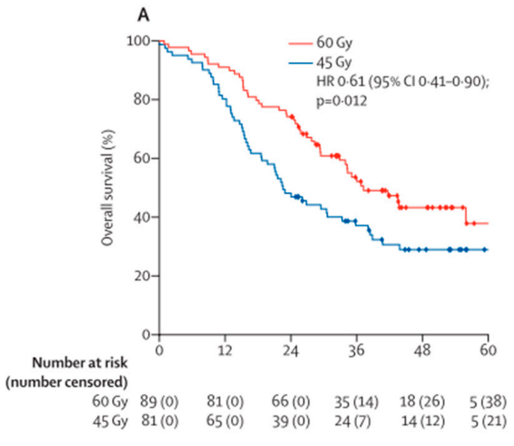

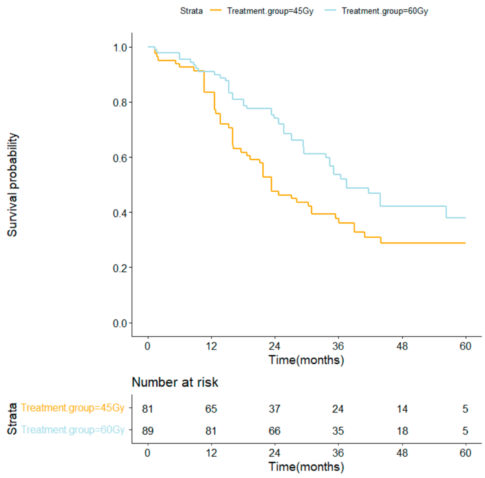

Han et al., 2008

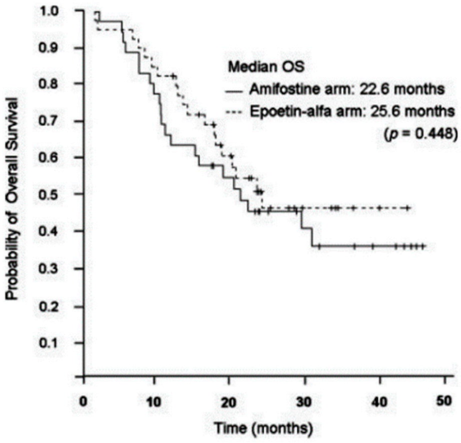

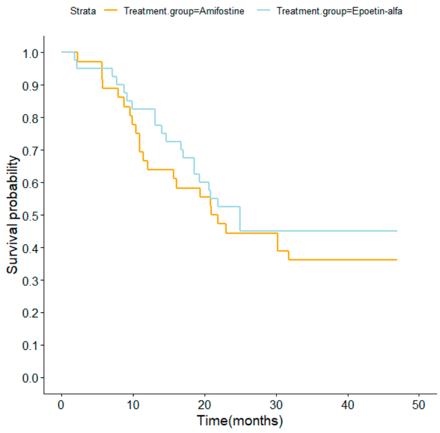

Han et al., 2005

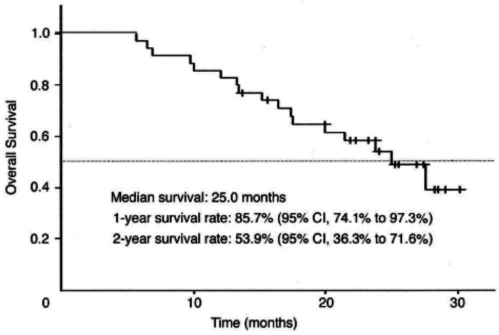

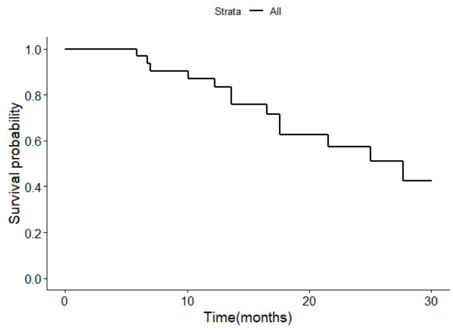

Hu et al., 2020

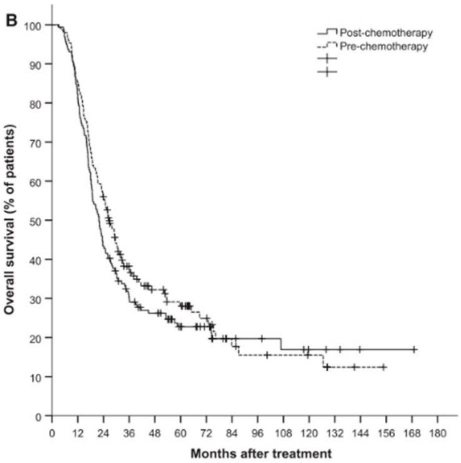

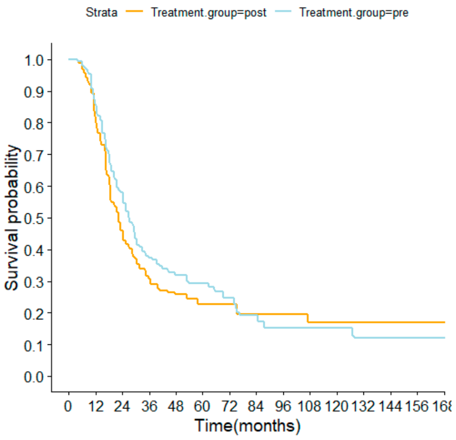

Hügli et al., 2000

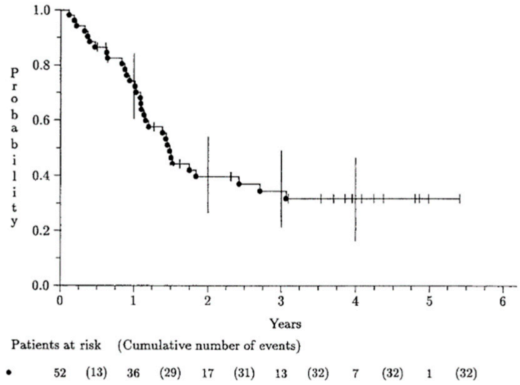

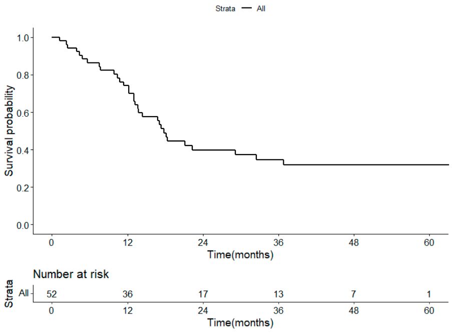

Jeremic et al., 1997

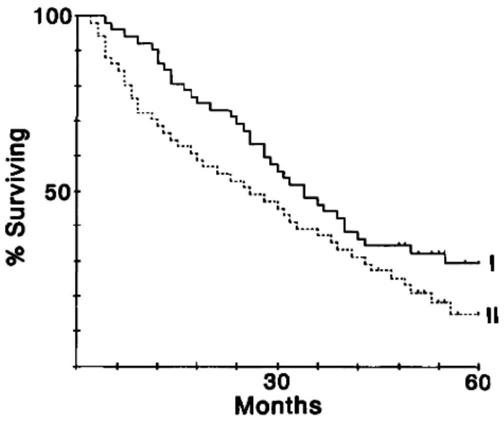

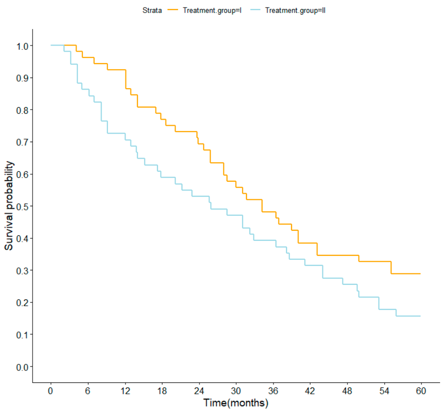

Johnson et al., 1996

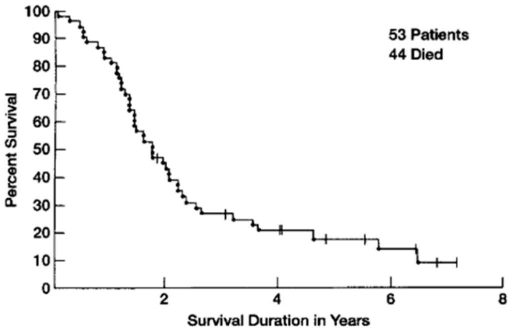

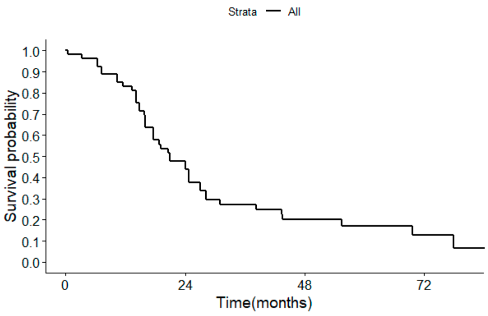

Kubota et al., 2014

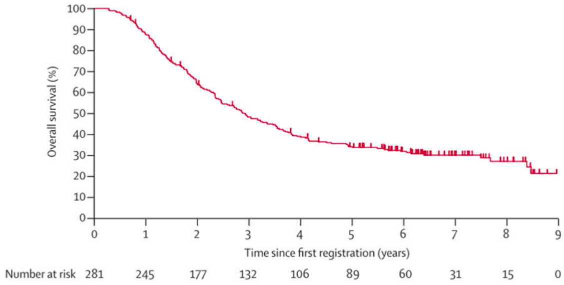

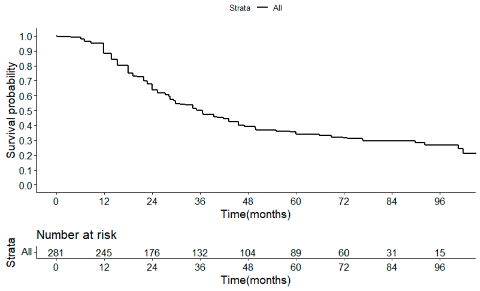

Mennecier et al., 2000

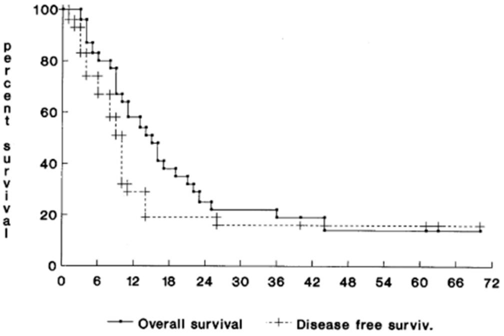

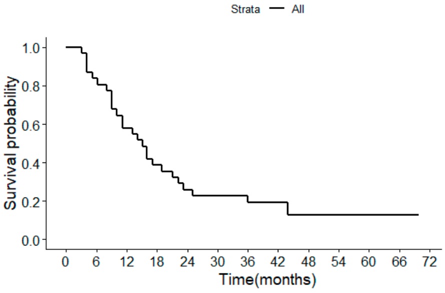

Saito et al., 2006

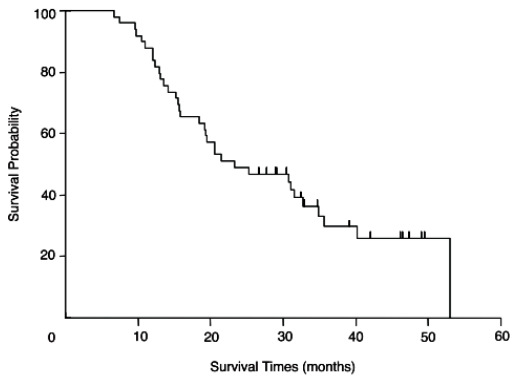

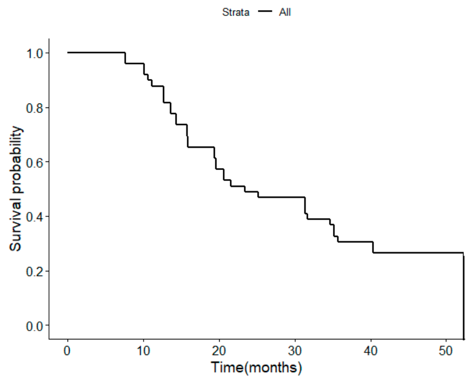

Schild et al., 2007

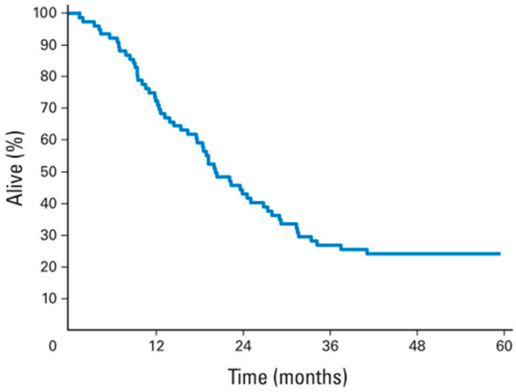

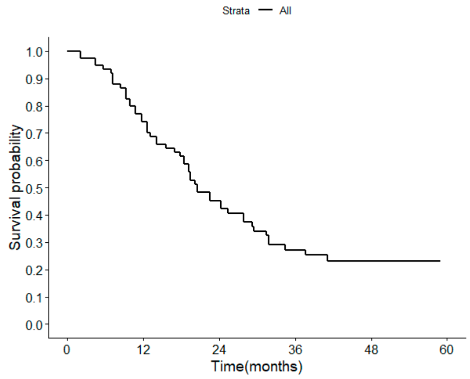

Skarlos et al., 2001

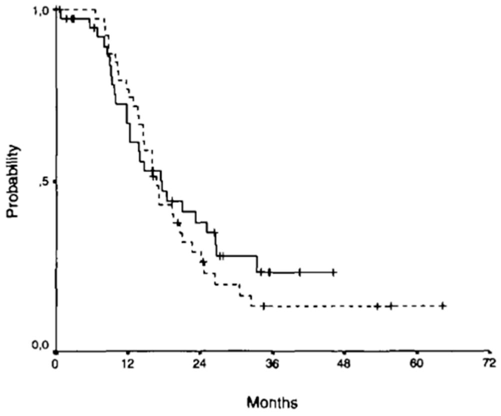

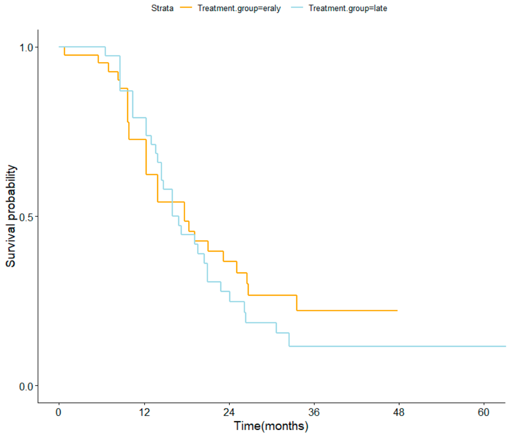

Sorensen et al., 2008

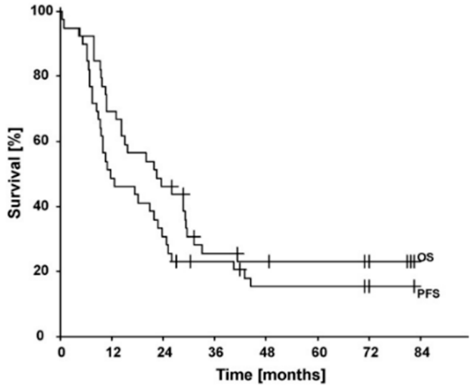

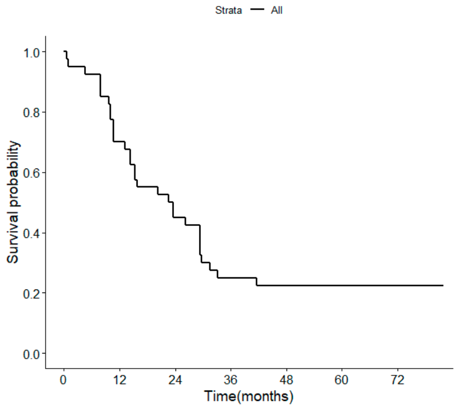

Takada et al., 2002

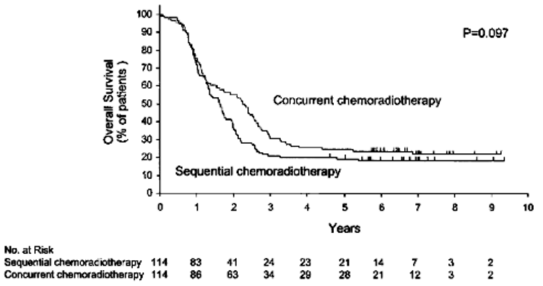

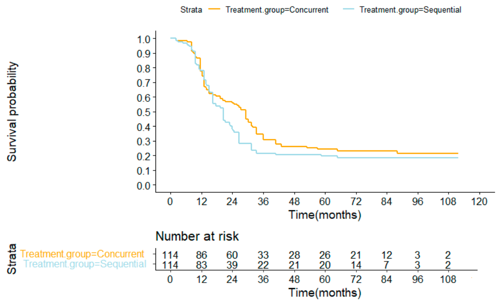

van Loon et al., 2010

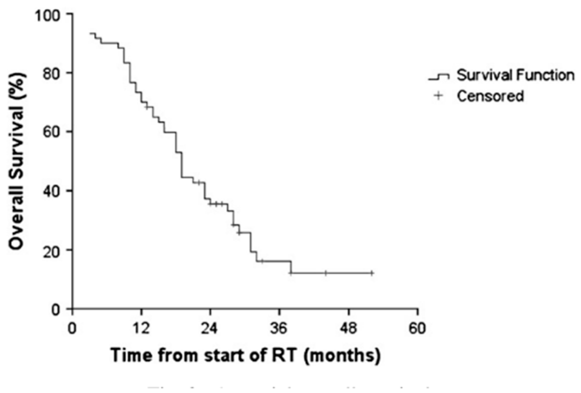

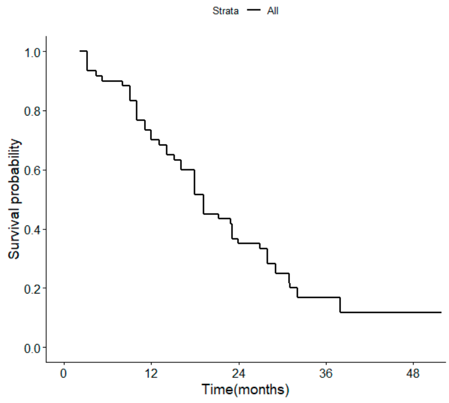

Figure S3. Network plots.

RCTs

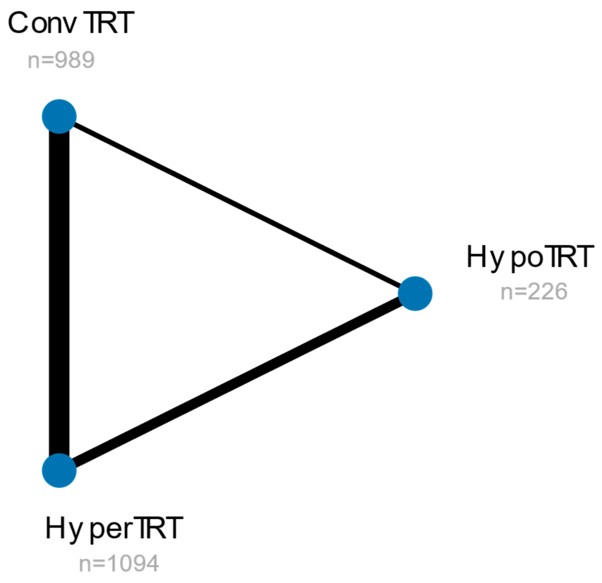

Subgroup of 3D-CRT/IMRT

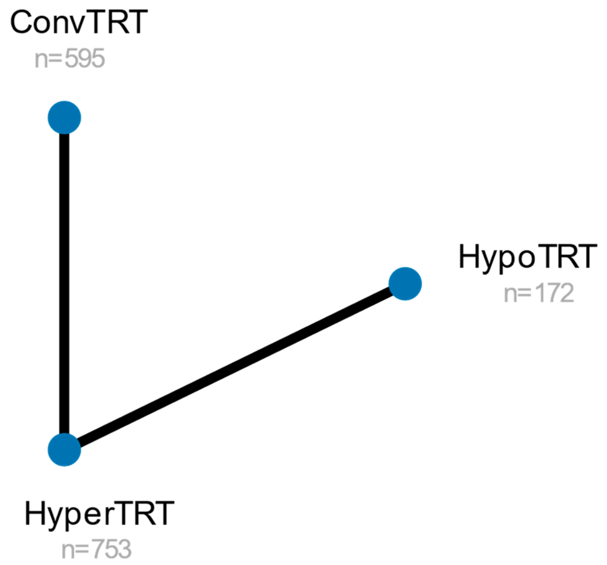

RCTs, randomized controlled trials; HypoTRT, hypofractionated thoracic radiotherapy; ConvTRT, conventional fractionated thoracic radiotherapy; HyperTRT, hyperfractionated thoracic radiotherapy; 3D-CRT, three-dimensional conformal; IMRT, intensity modulated radiotherapy.

Figure S4. Forest plots of severe radiation related events for altered fractionation modalities.

Grade 3-5 radiation esophagitis for all enrolled studies.

Study	RT	Grade 3−5 RE	95% CI	W.Fixed	W.Random
Study.types = Randomized controlled trials					
Groenberg et al., 2016	HypoTRT	0.31	[0.21; 0.42]	6.6%	9.9%
Qiu et al., 2021	HypoTRT	0.15	[0.08; 0.25]	6.6%	9.9%
Blackstock el al., 2005	HypoTRT	0.04	[0.00; 0.13]	4.2%	9.4%
Common efect model		0.17	[0.12; 0.22]	17.4%	--
Random efects model		0.15	[0.03; 0.34]	--	29.2%
Heterogeneity: $I^2 = 90\%$, $\tau^2 = 0.0318$, $\chi^2_2 = 19.28$ ($p < 0.01$)					
Study.types = Observational studies					
Yan et al., 2021	HypoTRT	0.14	[0.07; 0.25]	4.9%	9.6%
Socha et al., 2015	HypoTRT	0.00	[0.00; 0.04]	7.8%	10.1%
Zhang et al., 2017	HypoTRT	0.12	[0.05; 0.22]	5.4%	9.7%
Common efect model		0.05	[0.02; 0.08]	18.1%	--
Random efects model		0.06	[0.00; 0.21]	--	29.3%
Heterogeneity: $I^2 = 92\%$, $\tau^2 = 0.0332$, $\chi^2_2 = 24.37$ ($p < 0.01$)					
Study.types = Prospective non-RCT studies					
Gregor et al., 1997	HypoTRT	0.03	[0.01; 0.05]	26.0%	10.8%
Jett et al., 1990	HypoTRT	0.05	[0.02; 0.08]	18.0%	10.6%
Sculier et al., 2008	HypoTRT	0.06	[0.03; 0.11]	15.9%	10.6%
Xia et al., 2015	HypoTRT	0.25	[0.15; 0.38]	4.6%	9.5%
Common efect model		0.05	[0.04; 0.07]	64.4%	--
Random efects model		0.08	[0.02; 0.18]	--	41.4%
Heterogeneity: $I^2 = 89\%$, $\tau^2 = 0.0207$, $\chi^2_3 = 26.1$ ($p < 0.01$)					
**Common effect model**		**0.07**	**[0.05; 0.08]**	**100.0%**	**--**
**Random effects model**		**0.09**	**[0.04; 0.16]**	**--**	**100.0%**

Heterogeneity: $I^2 = 91\%$, $\tau^2 = 0.0250$, $\chi^2_9 = 97.62$ ($p < 0.01$)
Test for subgroup diferences (fixed effect): $\chi^2_2 = 27.87$, df = 2 ($p < 0.01$)
Test for subgroup diferences (random efects): $\chi^2_2 = 1.04$, df = 2 ($p = 0.59$)

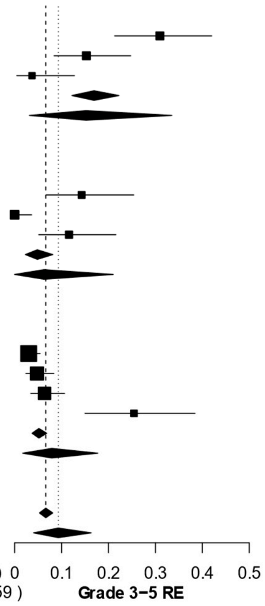

Study	RT	Grade 3−5 RE	95% CI	W.Fixed	W.Random
Study.types = Randomized controlled trials					
Turrisi et al., 1999	ConvTRT	0.16	[0.11; 0.22]	9.4%	5.6%
Bonner et al., 1999	ConvTRT	0.05	[0.02; 0.11]	6.1%	5.4%
Faivre−Finn et al., 2017	ConvTRT	0.19	[0.14; 0.25]	11.4%	5.6%
Bogart et al., 2021	ConvTRT	0.19	[0.14; 0.23]	13.9%	5.7%
Blackstock el al., 2005	ConvTRT	0.09	[0.03; 0.20]	2.5%	4.9%
Common efect model		0.15	[0.13; 0.18]	43.3%	--
Random efects model		0.14	[0.08; 0.20]	--	27.2%
Heterogeneity: $I^2 = 81\%$, $\tau^2 = 0.0069$, $\chi^2_4 = 20.66$ ($p < 0.01$)					
Study.types = Observational studies					
Gazula et al., 2014	ConvTRT	0.00	[0.00; 0.18]	0.9%	3.7%
Han et al., 2015	ConvTRT	0.06	[0.02; 0.14]	3.7%	5.2%
Socha et al., 2015	ConvTRT	0.00	[0.00; 0.04]	3.8%	5.2%
Zhang et al., 2017	ConvTRT	0.10	[0.05; 0.17]	4.7%	5.3%
Common efect model		0.04	[0.02; 0.06]	13.1%	--
Random efects model		0.03	[0.00; 0.10]	--	19.4%
Heterogeneity: $I^2 = 80\%$, $\tau^2 = 0.0136$, $\chi^2_3 = 15.07$ ($p < 0.01$)					
Study.types = Prospective non-RCT studies					
Baas et al., 2006	ConvTRT	0.27	[0.14; 0.44]	1.7%	4.5%
Hanna et al., 2002	ConvTRT	0.04	[0.00; 0.13]	2.5%	4.9%
Kakolyris et al., 2006	ConvTRT	0.15	[0.05; 0.32]	1.5%	4.4%
Kelley et al., 2013	ConvTRT	0.22	[0.13; 0.34]	3.3%	5.1%
Le et al., 2009	ConvTRT	0.25	[0.15; 0.37]	3.2%	5.1%
McClay et al., 2005	ConvTRT	0.21	[0.16; 0.26]	14.2%	5.7%
Miller et al., 2007	ConvTRT	0.32	[0.20; 0.47]	2.3%	4.8%
Sohn et al., 2007	ConvTRT	0.18	[0.07; 0.35]	1.5%	4.4%
Sun et al., 2013	ConvTRT	0.02	[0.01; 0.05]	10.1%	5.6%
Wahba et al., 2012	ConvTRT	0.03	[0.00; 0.16]	1.5%	4.4%
Xenidis et al., 2010	ConvTRT	0.00	[0.00; 0.11]	1.5%	4.4%
Common efect model		0.13	[0.11; 0.15]	43.6%	--
Random efects model		0.13	[0.07; 0.21]	--	53.3%
Heterogeneity: $I^2 = 90\%$, $\tau^2 = 0.0273$, $\chi^2_{10} = 100.84$ ($p < 0.01$)					
**Common effect model**		**0.12**	**[0.11; 0.14]**	**100.0%**	**--**
**Random effects model**		**0.11**	**[0.07; 0.16]**	**--**	**100.0%**

Heterogeneity: $I^2 = 89\%$, $\tau^2 = 0.0220$, $\chi^2_{19} = 168.77$ ($p < 0.01$)
Test for subgroup diferences (fixed effect): $\chi^2_2 = 32.20$, df = 2 ($p < 0.01$)
Test for subgroup diferences (random efects): $\chi^2_2 = 6.01$, df = 2 ($p = 0.05$)

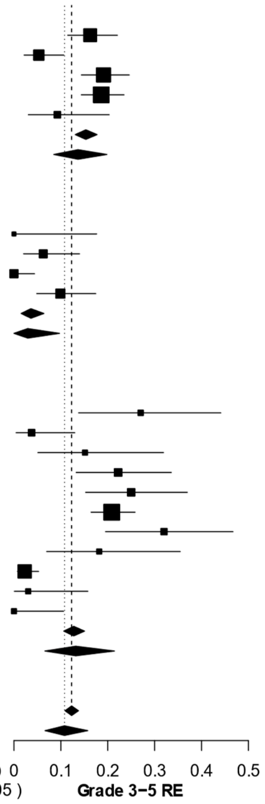

Study	RT	Grade 3−5 RE	95%CI	W.Fixed	W.Random
Study.types = Randomized controlled trials					
Groenberg et al., 2016	HyperTRT	0.33	[0.22; 0.45]	2.5%	3.8%
Qiu et al., 2021	HyperTRT	0.17	[0.10; 0.27]	3.1%	4.0%
Turrisi et al., 1999	HyperTRT	0.33	[0.26; 0.39]	6.9%	4.2%
Bonner et al., 1999	HyperTRT	0.12	[0.07; 0.19]	4.4%	4.1%
Faivre−Finn et al., 2017	HyperTRT	0.19	[0.14; 0.24]	8.5%	4.2%
Bogart JA et al., 2021	HyperTRT	0.17	[0.13; 0.21]	9.9%	4.3%
Common efect model		0.20	[0.18; 0.23]	35.3%	--
Random efects model		0.21	[0.15; 0.28]	--	24.6%
Heterogeneity: I^2= 84% , τ^2= 0.0086 , χ^2_5= 31.33 (p < 0.01)					
Study.types = Observational studies					
Yan et al., 2021	HyperTRT	0.13	[0.07; 0.20]	3.7%	4.0%
Gazula et al., 2014	HyperTRT	0.00	[0.00; 0.13]	0.9%	3.1%
Han et al., 2015	HyperTRT	0.19	[0.10; 0.31]	2.1%	3.8%
Common efect model		0.12	[0.07; 0.17]	6.7%	--
Random efects model		0.09	[0.01; 0.24]	--	10.9%
Heterogeneity: I^2= 80% , τ^2= 0.0257 , χ^2_2= 9.81 (p < 0.01)					
Study.types = Prospective non-RCT studies					
Chen et al., 2005	HyperTRT	0.07	[0.02; 0.17]	1.9%	3.7%
Ettinger et al., 2005	HyperTRT	0.36	[0.23; 0.50]	1.8%	3.7%
Groenberg et al., 2021	HyperTRT	0.20	[0.14; 0.27]	5.6%	4.1%
Han et al., 2008	HyperTRT	0.21	[0.12; 0.32]	2.4%	3.8%
Han et al., 2005	HyperTRT	0.31	[0.17; 0.49]	1.2%	3.4%
Hu et al., 2020	HyperTRT	0.11	[0.07; 0.15]	10.1%	4.3%
Hügli et al., 2000	HyperTRT	0.38	[0.25; 0.53]	1.8%	3.7%
Jeremic et al., 1997	HyperTRT	0.27	[0.19; 0.37]	3.5%	4.0%
Johnson et al., 1996	HyperTRT	0.26	[0.15; 0.40]	1.8%	3.7%
Kubota et al., 2014	HyperTRT	0.02	[0.01; 0.04]	9.2%	4.2%
Mennecier et al., 2000	HyperTRT	0.50	[0.31; 0.69]	1.0%	3.3%
Saito et al., 2006	HyperTRT	0.02	[0.00; 0.11]	1.7%	3.6%
Schild et al., 2007	HyperTRT	0.25	[0.16; 0.36]	2.6%	3.9%
Skarlos et al., 2001	HyperTRT	0.10	[0.04; 0.19]	2.7%	3.9%
Sorensen et al., 2008	HyperTRT	0.26	[0.13; 0.44]	1.2%	3.4%
Takada et al., 2002	HyperTRT	0.06	[0.03; 0.10]	7.5%	4.2%
van Loon et al., 2010	HyperTRT	0.12	[0.05; 0.23]	2.0%	3.7%
Common efect model		0.13	[0.11; 0.14]	58.0%	--
Random efects model		0.18	[0.12; 0.25]	--	64.5%
Heterogeneity: I^2= 91% , τ^2= 0.0286 , χ^2_{16}= 185.12 (p < 0.01)					
**Common effect model**		**0.15**	**[0.14; 0.17]**	**100.0%**	**--**
**Random effects model**		**0.18**	**[0.13; 0.23]**	**--**	**100.0%**

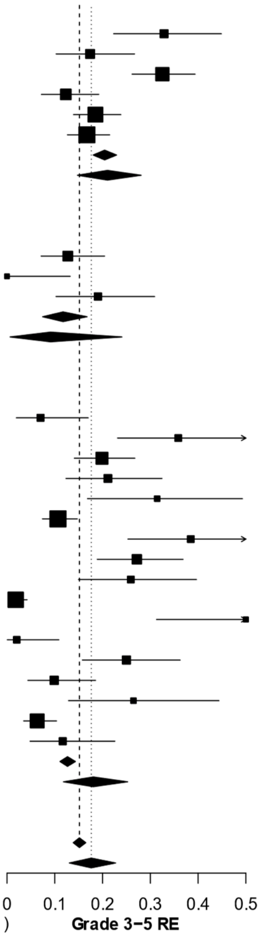

Heterogeneity: I^2= 90% , τ^2= 0.0228 , χ^2_{25}= 253.84 (p < 0.01)
Test for subgroup diferences (fixed effect): χ^2_2= 27.58, df = 2 (p < 0.01)
Test for subgroup diferences (random efects): χ^2_2= 2.13, df = 2 (p = 0.34)

Grade 3-5 radiation pneumonia for all enrolled studies.

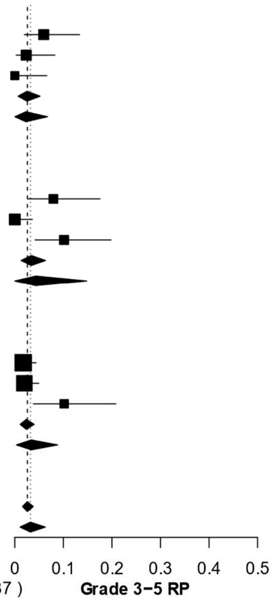

Study	RT	Grade 3−5 RP	95% CI	W.Fixed	W.Random
Study.types = Randomized controlled trials					
Groenberg et al., 2016	HypoTRT	0.06	[0.02; 0.13]	8.9%	11.0%
Qiu et al., 2021	HypoTRT	0.02	[0.00; 0.08]	9.0%	11.1%
Blackstock el al., 2005	HypoTRT	0.00	[0.00; 0.07]	5.7%	9.6%
Common efect model		0.03	[0.01; 0.05]	23.5%	--
Random efects model		0.02	[0.00; 0.07]	--	31.7%
Heterogeneity: I^2= 58% , τ^2= 0.0049 , χ^2_2= 4.78 (p= 0.09)					
Study.types = Observational studies					
Yan et al., 2021	HypoTRT	0.08	[0.03; 0.18]	6.7%	10.1%
Socha et al., 2015	HypoTRT	0.00	[0.00; 0.04]	10.5%	11.5%
Zhang et al., 2017	HypoTRT	0.10	[0.04; 0.20]	7.3%	10.4%
Common efect model		0.03	[0.01; 0.06]	24.5%	--
Random efects model		0.04	[0.00; 0.15]	--	32.1%
Heterogeneity: I^2= 88% , τ^2= 0.0213 , χ^2_2= 16.4 (p< 0.01)					
Study.types = Prospective non-RCT studies					
Jett et al., 1990	HypoTRT	0.02	[0.00; 0.04]	24.3%	13.3%
Sculier et al., 2008	HypoTRT	0.02	[0.01; 0.05]	21.4%	13.1%
Xia et al., 2015	HypoTRT	0.10	[0.04; 0.21]	6.2%	9.9%
Common efect model		0.02	[0.01; 0.04]	52.0%	--
Random efects model		0.03	[0.00; 0.09]	--	36.3%
Heterogeneity: I^2= 74% , τ^2= 0.0078 , χ^2_2= 7.71 (p= 0.02)					
**Common effect model**		**0.03**	**[0.02; 0.04]**	**100.0%**	**--**
**Random effects model**		**0.03**	**[0.01; 0.06]**	**--**	**100.0%**

Heterogeneity: I^2= 73% , τ^2= 0.0081 , χ^2_8= 29.66 (p< 0.01)
Test for subgroup diferences (fixed effect): χ^2_2= 0.77, df = 2 (p= 0.68)
Test for subgroup diferences (random efects): χ^2_2= 0.28, df = 2 (p= 0.87)

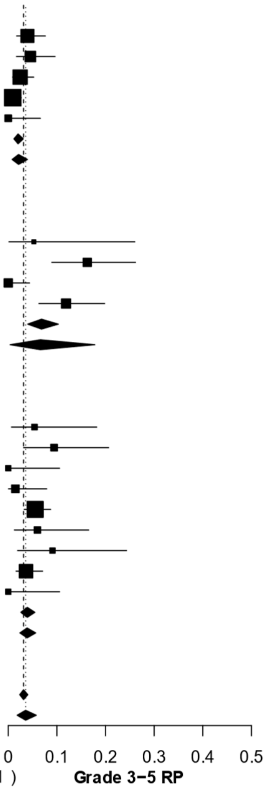

Study	RT	Grade 3−5 RP	95% CI	W.Fixed	W.Random
Study.types = Randomized controlled trials					
Turrisi et al., 1999	ConvTRT	0.04	[0.02; 0.08]	9.8%	7.0%
Bonner et al., 1999	ConvTRT	0.05	[0.02; 0.10]	6.4%	6.5%
Faivre−Finn et al., 2017	ConvTRT	0.02	[0.01; 0.05]	11.8%	7.2%
Bogart et al., 2021	ConvTRT	0.01	[0.00; 0.03]	15.6%	7.4%
Blackstock el al., 2005	ConvTRT	0.00	[0.00; 0.07]	2.6%	5.0%
Common efect model		0.02	[0.01; 0.03]	46.2%	--
Random efects model		0.02	[0.01; 0.04]	--	33.1%
Heterogeneity: I^2= 59% , τ^2= 0.0019 , χ^2_4= 9.68 (p= 0.05)					
Study.types = Observational studies					
Gazula et al., 2014	ConvTRT	0.05	[0.00; 0.26]	0.9%	3.0%
Han et al., 2015	ConvTRT	0.16	[0.09; 0.26]	3.9%	5.7%
Socha et al., 2015	ConvTRT	0.00	[0.00; 0.04]	4.0%	5.8%
Zhang et al., 2017	ConvTRT	0.12	[0.06; 0.20]	4.9%	6.1%
Common efect model		0.07	[0.04; 0.10]	13.6%	--
Random efects model		0.07	[0.00; 0.18]	--	20.7%
Heterogeneity: I^2= 88% , τ^2= 0.0243 , χ^2_3= 25.5 (p< 0.01)					
Study.types = Prospective non-RCT studies					
Baas et al., 2006	ConvTRT	0.05	[0.01; 0.18]	1.8%	4.3%
Hanna et al., 2002	ConvTRT	0.09	[0.03; 0.21]	2.6%	5.0%
Kakolyris et al., 2006	ConvTRT	0.00	[0.00; 0.11]	1.6%	4.1%
Le et al., 2009	ConvTRT	0.01	[0.00; 0.08]	3.3%	5.5%
McClay et al., 2005	ConvTRT	0.06	[0.03; 0.09]	14.8%	7.3%
Miller et al., 2007	ConvTRT	0.06	[0.01; 0.17]	2.4%	4.9%
Sohn et al., 2007	ConvTRT	0.09	[0.02; 0.24]	1.6%	4.1%
Sun et al., 2013	ConvTRT	0.04	[0.02; 0.07]	10.5%	7.1%
Wahba et al., 2012	ConvTRT	0.00	[0.00; 0.11]	1.6%	4.1%
Common efect model		0.04	[0.03; 0.05]	40.2%	--
Random efects model		0.04	[0.02; 0.06]	--	46.3%
Heterogeneity: I^2= 32% , τ^2= 0.0004 , χ^2_8= 11.72 (p= 0.16)					
**Common effect model**		**0.03**	**[0.02; 0.04]**	**100.0%**	**--**
**Random effects model**		**0.04**	**[0.02; 0.06]**	**--**	**100.0%**

Heterogeneity: I^2= 73% , τ^2= 0.0075 , χ^2_{17}= 63.77 (p< 0.01)
Test for subgroup diferences (fixed effect): χ^2_2= 16.86, df = 2 (p< 0.01)
Test for subgroup diferences (random efects): χ^2_2= 4.42, df = 2 (p= 0.11)

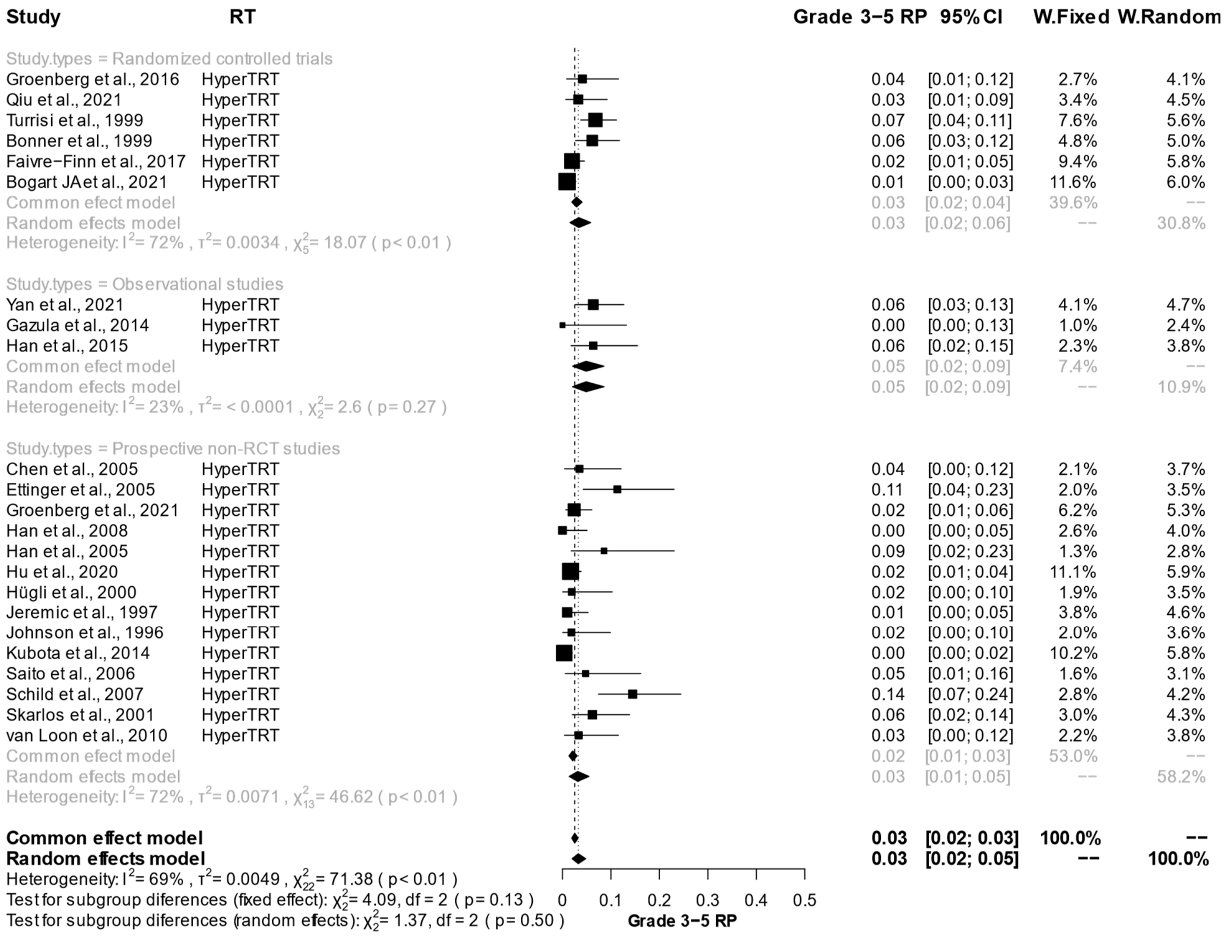
Study RT Grade 3−5 RP 95% CI W.Fixed W.Random
Study.types = Randomized controlled trials
Groenberg et al., 2016 HyperTRT 0.04 [0.01; 0.12] 2.7% 4.1%
Qiu et al., 2021 HyperTRT 0.03 [0.01; 0.09] 3.4% 4.5%
Turrisi et al., 1999 HyperTRT 0.07 [0.04; 0.11] 7.6% 5.6%
Bonner et al., 1999 HyperTRT 0.06 [0.03; 0.12] 4.8% 5.0%
Faivre−Finn et al., 2017 HyperTRT 0.02 [0.01; 0.05] 9.4% 5.8%
Bogart JA et al., 2021 HyperTRT 0.01 [0.00; 0.03] 11.6% 6.0%
Common efect model 0.03 [0.02; 0.04] 39.6% --
Random efects model 0.03 [0.02; 0.06] -- 30.8%
Heterogeneity: I^2 = 72% , τ^2 = 0.0034 , χ^2_5 = 18.07 (p < 0.01)
Study.types = Observational studies
Yan et al., 2021 HyperTRT 0.06 [0.03; 0.13] 4.1% 4.7%
Gazula et al., 2014 HyperTRT 0.00 [0.00; 0.13] 1.0% 2.4%
Han et al., 2015 HyperTRT 0.06 [0.02; 0.15] 2.3% 3.8%
Common efect model 0.05 [0.02; 0.09] 7.4% --
Random efects model 0.05 [0.02; 0.09] -- 10.9%
Heterogeneity: I^2 = 23% , τ^2 = < 0.0001 , χ^2_2 = 2.6 (p = 0.27)
Study.types = Prospective non-RCT studies
Chen et al., 2005 HyperTRT 0.04 [0.00; 0.12] 2.1% 3.7%
Ettinger et al., 2005 HyperTRT 0.11 [0.04; 0.23] 2.0% 3.5%
Groenberg et al., 2021 HyperTRT 0.02 [0.01; 0.06] 6.2% 5.3%
Han et al., 2008 HyperTRT 0.00 [0.00; 0.05] 2.6% 4.0%
Han et al., 2005 HyperTRT 0.09 [0.02; 0.23] 1.3% 2.8%
Hu et al., 2020 HyperTRT 0.02 [0.01; 0.04] 11.1% 5.9%
Hügli et al., 2000 HyperTRT 0.02 [0.00; 0.10] 1.9% 3.5%
Jeremic et al., 1997 HyperTRT 0.01 [0.00; 0.05] 3.8% 4.6%
Johnson et al., 1996 HyperTRT 0.02 [0.00; 0.10] 2.0% 3.6%
Kubota et al., 2014 HyperTRT 0.00 [0.00; 0.02] 10.2% 5.8%
Saito et al., 2006 HyperTRT 0.05 [0.01; 0.16] 1.6% 3.1%
Schild et al., 2007 HyperTRT 0.14 [0.07; 0.24] 2.8% 4.2%
Skarlos et al., 2001 HyperTRT 0.06 [0.02; 0.14] 3.0% 4.3%
van Loon et al., 2010 HyperTRT 0.03 [0.00; 0.12] 2.2% 3.8%
Common efect model 0.02 [0.01; 0.03] 53.0% --
Random efects model 0.03 [0.01; 0.05] -- 58.2%
Heterogeneity: I^2 = 72% , τ^2 = 0.0071 , χ^2_{13} = 46.62 (p < 0.01)
Common effect model 0.03 [0.02; 0.03] 100.0% --
Random effects model 0.03 [0.02; 0.05] -- 100.0%
Heterogeneity: I^2 = 69% , τ^2 = 0.0049 , χ^2_{22} = 71.38 (p < 0.01)
Test for subgroup diferences (fixed effect): χ^2_2 = 4.09, df = 2 (p = 0.13)
Test for subgroup diferences (random efects): χ^2_2 = 1.37, df = 2 (p = 0.50)
0 0.1 0.2 0.3 0.4 0.5
Grade 3−5 RP

Grade 3-5 radiation esophagitis for subgroup of 3D-CRT/IMRT

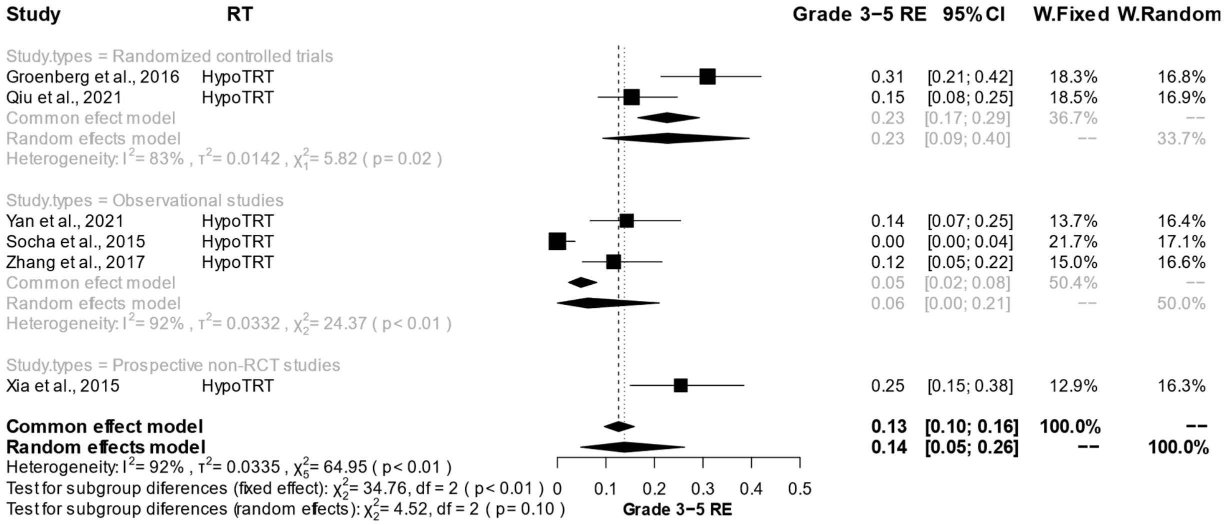

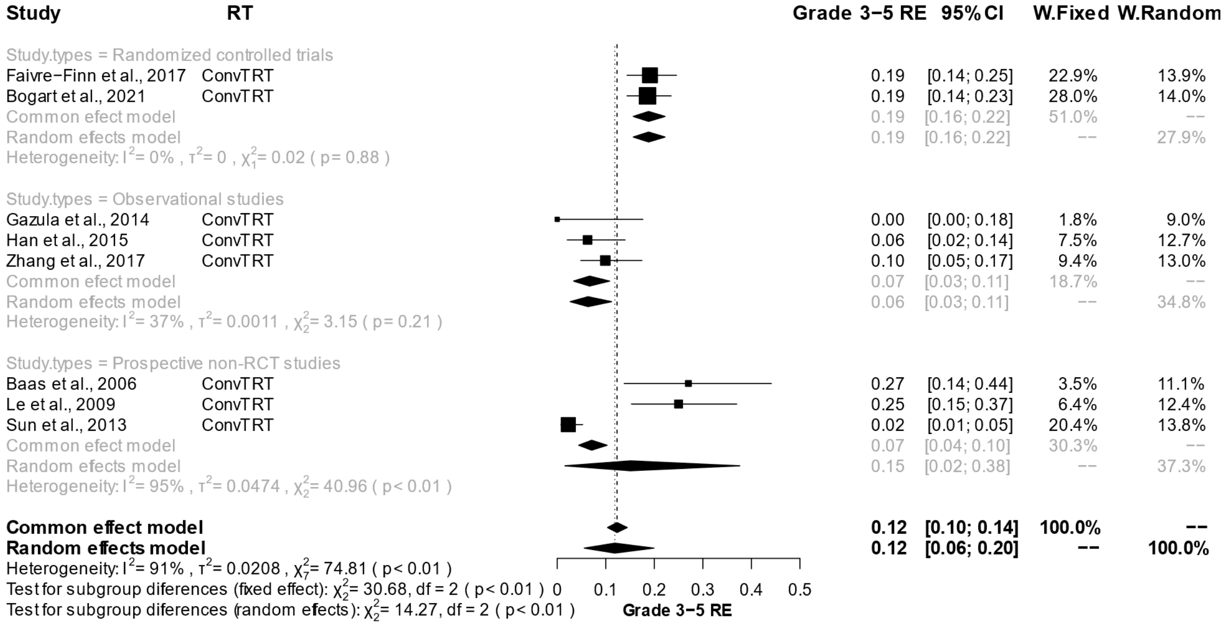

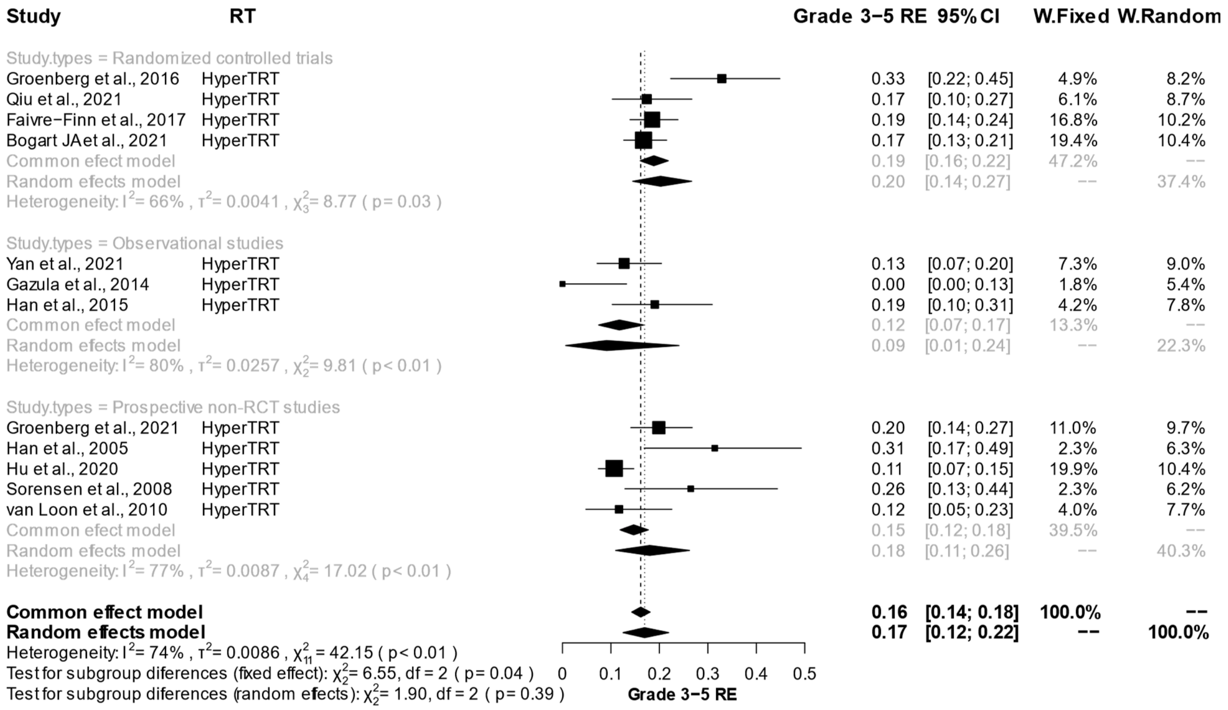
Study RT Grade 3−5 RE 95% CI W.Fixed W.Random
Study.types = Randomized controlled trials
Groenberg et al., 2016 HyperTRT 0.33 [0.22; 0.45] 4.9% 8.2%
Qiu et al., 2021 HyperTRT 0.17 [0.10; 0.27] 6.1% 8.7%
Faivre−Finn et al., 2017 HyperTRT 0.19 [0.14; 0.24] 16.8% 10.2%
Bogart JA et al., 2021 HyperTRT 0.17 [0.13; 0.21] 19.4% 10.4%
Common efect model 0.19 [0.16; 0.22] 47.2% --
Random efects model 0.20 [0.14; 0.27] -- 37.4%
Heterogeneity: I^2= 66% , τ^2= 0.0041 , χ^2_3= 8.77 (p= 0.03)
Study.types = Observational studies
Yan et al., 2021 HyperTRT 0.13 [0.07; 0.20] 7.3% 9.0%
Gazula et al., 2014 HyperTRT 0.00 [0.00; 0.13] 1.8% 5.4%
Han et al., 2015 HyperTRT 0.19 [0.10; 0.31] 4.2% 7.8%
Common efect model 0.12 [0.07; 0.17] 13.3% --
Random efects model 0.09 [0.01; 0.24] -- 22.3%
Heterogeneity: I^2= 80% , τ^2= 0.0257 , χ^2_2= 9.81 (p< 0.01)
Study.types = Prospective non-RCT studies
Groenberg et al., 2021 HyperTRT 0.20 [0.14; 0.27] 11.0% 9.7%
Han et al., 2005 HyperTRT 0.31 [0.17; 0.49] 2.3% 6.3%
Hu et al., 2020 HyperTRT 0.11 [0.07; 0.15] 19.9% 10.4%
Sorensen et al., 2008 HyperTRT 0.26 [0.13; 0.44] 2.3% 6.2%
van Loon et al., 2010 HyperTRT 0.12 [0.05; 0.23] 4.0% 7.7%
Common efect model 0.15 [0.12; 0.18] 39.5% --
Random efects model 0.18 [0.11; 0.26] -- 40.3%
Heterogeneity: I^2= 77% , τ^2= 0.0087 , χ^2_4= 17.02 (p< 0.01)
Common effect model 0.16 [0.14; 0.18] 100.0% --
Random effects model 0.17 [0.12; 0.22] -- 100.0%
Heterogeneity: I^2= 74% , τ^2= 0.0086 , χ^2_{11} = 42.15 (p< 0.01)
Test for subgroup diferences (fixed effect): χ^2_2= 6.55, df = 2 (p= 0.04)
Test for subgroup diferences (random efects): χ^2_2= 1.90, df = 2 (p= 0.39)
0 0.1 0.2 0.3 0.4 0.5
Grade 3−5 RE

Grade 3-5 radiation pneumonia for subgroup of 3D-CRT/IMRT

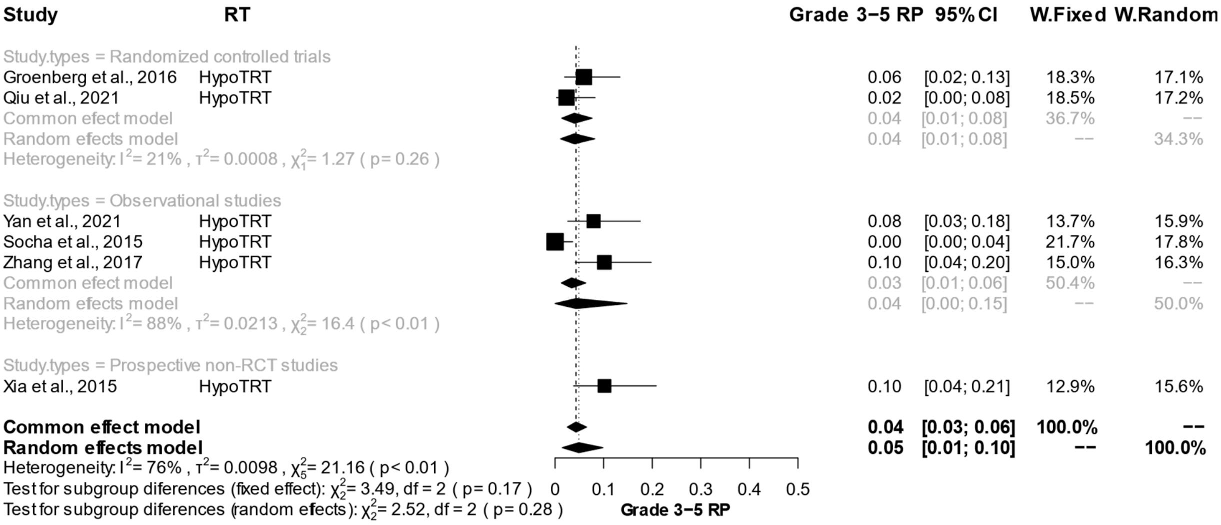

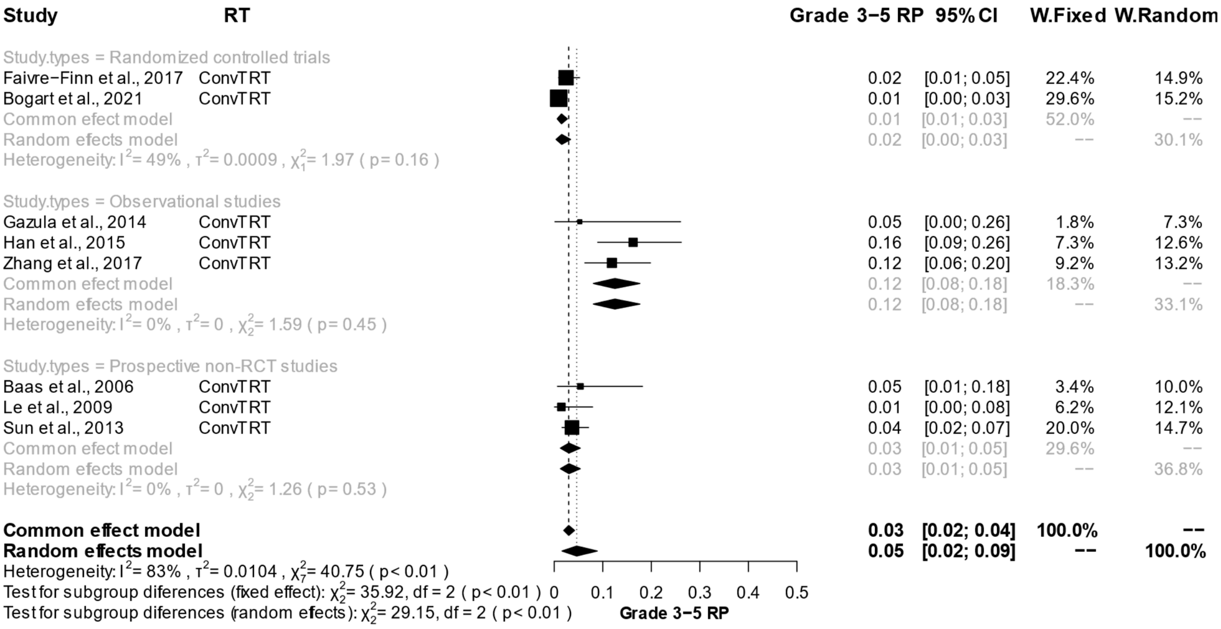

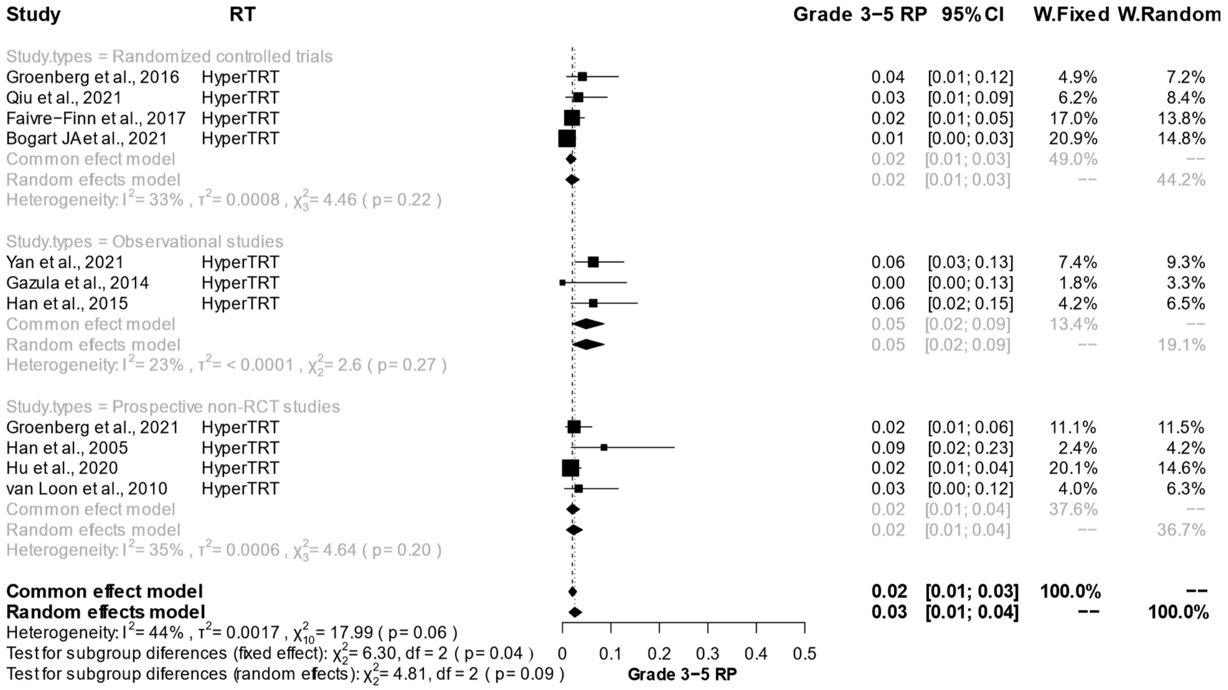

RT, radiation technique; RE, radiation esophagitis; RP, radiation pneumonia; CI, confidence interval; HypoTRT, hypofractionated thoracic radiotherapy; ConvTRT, conventional fractionated thoracic radiotherapy; HyperTRT, hyperfractionated thoracic radiotherapy; 3D-CRT, three-dimensional conformal; IMRT, intensity modulated radiotherapy.

Figure S5. Funnel plot according to double arcsine transformed proportion for publication bias.

Grade 3-5 radiation esophagitis for all enrolled studies.

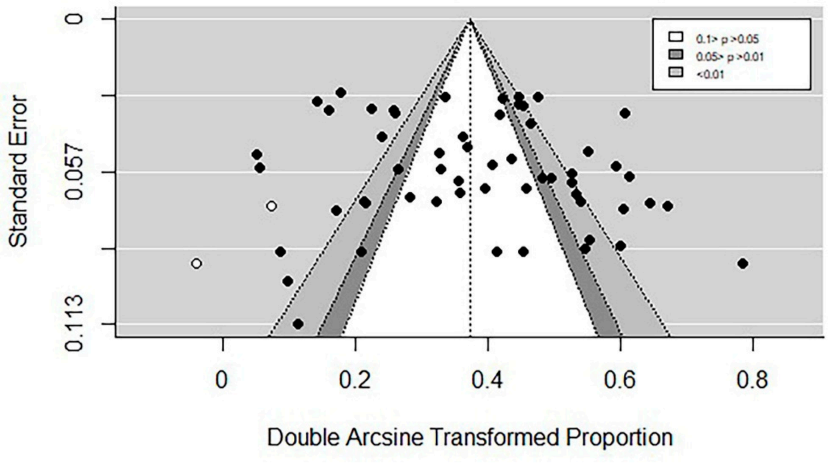

Grade 3-5 radiation pneumonia for all enrolled studies.

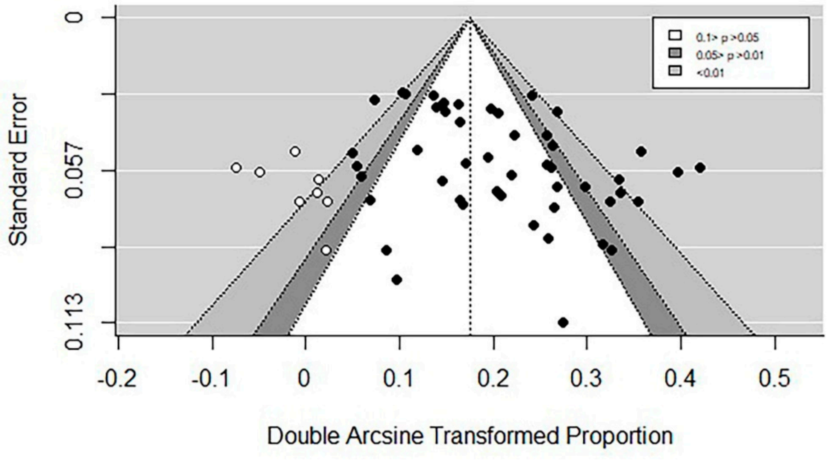

Supplementary References

1. Fowler, J.F. The linear-quadratic formula and progress in fractionated radiotherapy. *Br J Radiol* **1989**, *62*, 679-694.
2. Shibamoto, Y.; Ike, O.; Mizuno, H.; Fukuse, T.; Hitomi, S.; Takahashi, M. Proliferative activity and micronucleus frequency after radiation of lung cancer cells as assessed by the cytokinesis-block method and their relationship to clinical outcome. *Clin Cancer Res* **1998**, *4*, 677-682.
3. Tinnemans, M.M.; Schutte, B.; Lenders, M.H.; Ten Velde, G.P.; Ramaekers, F.C.; Blijham, G.H. Cytokinetic analysis of lung cancer by in vivo bromodeoxyuridine labelling. *British Journal of Cancer* **1993**, *67*, 1217-1222.
4. Ganti, A.K.P.; Loo, B.W.; Bassetti, M.; Blakely, C.; Chiang, A.; D'Amico, T.A.; D'Avella, C.; Dowlati, A.; Downey, R.J.; Edelman, M., et al. Small Cell Lung Cancer, Version 2.2022, NCCN Clinical Practice Guidelines in Oncology. *J Natl Compr Canc Netw* **2021**, *19*, 1441-1464, doi:10.6004/jnccn.2021.0058.
5. Shim, S.R.; Kim, S.-J.; Lee, J.; Rücker, G. Network meta-analysis: application and practice using R software. *Epidemiol Health* **2019**, *41*, e2019013, doi:10.4178/epih.e2019013.
6. Higgins, J.P.; Thompson, S.G. Quantifying heterogeneity in a meta-analysis. *Stat Med* **2002**, *21*, 1539-1558, doi:10.1002/sim.1186.
7. Barendregt, J.J.; Doi, S.A.; Lee, Y.Y.; Norman, R.E.; Vos, T. Meta-analysis of prevalence. *Journal of epidemiology and community health* **2013**, *67*, 974-978, doi:10.1136/jech-2013-203104.
8. Grønberg, B.H.; Halvorsen, T.O.; Fløtten, Ø.; Brustugun, O.T.; Brunsvig, P.F.; Aasebø, U.; Bremnes, R.M.; Tollåli, T.; Hornslien, K.; Aksnessæther, B.Y., et al. Randomized phase II trial comparing twice daily hyperfractionated with once daily hypofractionated thoracic radiotherapy in limited disease small cell lung cancer. *Acta Oncol* **2016**, *55*, 591-597, doi:10.3109/0284186X.2015.1092584.
9. Qiu, B.; Li, Q.; Liu, J.; Huang, Y.; Pang, Q.; Zhu, Z.; Yang, X.; Wang, B.; Chen, L.; Fang, J., et al. Moderately Hypofractionated Once-Daily Compared With Twice-Daily Thoracic Radiation Therapy Concurrently With Etoposide and Cisplatin in Limited-Stage Small Cell Lung Cancer: A Multicenter, Phase II, Randomized Trial. *Int J Radiat Oncol Biol Phys* **2021**, *111*, 424-435, doi:10.1016/j.ijrobp.2021.05.003.
10. Turrisi, A.T.; Kim, K.; Blum, R.; Sause, W.T.; Livingston, R.B.; Komaki, R.; Wagner, H.; Aisner, S.; Johnson, D.H. Twice-daily compared with once-daily thoracic radiotherapy in limited small-cell lung cancer treated concurrently with cisplatin and etoposide. *N Engl J Med* **1999**, *340*, 265-271.
11. Bonner, J.A.; Sloan, J.A.; Shanahan, T.G.; Brooks, B.J.; Marks, R.S.; Krook, J.E.; Gerstner, J.B.; Maksymiuk, A.; Levitt, R.; Mailliard, J.A., et al. Phase III comparison of twice-daily split-course irradiation versus once-daily irradiation for patients with limited stage small-cell lung carcinoma. *J Clin Oncol* **1999**, *17*, 2681-2691, doi:10.1200/jco.1999.17.9.2681.
12. Faivre-Finn, C.; Snee, M.; Ashcroft, L.; Appel, W.; Barlesi, F.; Bhatnagar, A.; Bezjak, A.; Cardenal, F.; Fournel, P.; Harden, S., et al. Concurrent once-daily versus twice-daily chemoradiotherapy in patients with limited-stage small-cell lung cancer (CONVERT): an open-label, phase 3, randomised, superiority trial. *Lancet Oncol* **2017**, *18*, 1116-1125, doi:10.1016/S1470-2045(17)30318-2.
13. Bogart, J.A.; Wang, X.F.; Masters, G.A.; Gao, J.; Komaki, R.; Kuzma, C.S.; Heymach, J.; Petty, W.J.; Gaspar, L.E.; Waqar, S.N., et al. Phase 3 comparison of high-dose once-daily (QD) thoracic radiotherapy (TRT) with standard twice-daily (BID) TRT in limited stage small cell lung cancer (LSCLC): CALGB 30610 (Alliance)/RTOG 0538. *Journal of Clinical Oncology* **2021**, *39*, 8505-8505, doi:10.1200/JCO.2021.39.15_suppl.8505.
14. Blackstock, A.W.; Bogart, J.A.; Matthews, C.; Lovato, J.F.; McCoy, T.; Livengood, K.; Ho, C.; White, D.; Atkins, J.N.; Miller, A.A. Split-course versus continuous thoracic radiation therapy for limited-stage small-cell lung cancer: final report of a randomized phase III trial. *Clin Lung Cancer* **2005**, *6*, 287-292, doi:10.3816/CLC.2005.n.007.
15. Bettington, C.S.; Tripcony, L.; Bryant, G.; Hickey, B.; Pratt, G.; Fay, M. A retrospective analysis of survival outcomes for two different radiotherapy fractionation schedules given in the same

overall time for limited stage small cell lung cancer. *J Med Imaging Radiat Oncol* **2013**, *57*, 105-112, doi:10.1111/j.1754-9485.2012.02470.x.

16. Yan, M.; Sigurdson, S.; Greifer, N.; Kennedy, T.A.C.; Toh, T.S.; Lindsay, P.E.; Weiss, J.; Hueniken, K.; Yeung, C.; Sugumar, V., et al. A Comparison of Hypofractionated and Twice-Daily Thoracic Irradiation in Limited-Stage Small-Cell Lung Cancer: An Overlap-Weighted Analysis. *Cancers (Basel)* **2021**, *13*, doi:10.3390/cancers13122895.
17. Gazula, A.; Baldini, E.H.; Chen, A.; Kozono, D. Comparison of once and twice daily radiotherapy for limited stage small-cell lung cancer. *Lung* **2014**, *192*, 151-158, doi:10.1007/s00408-013-9518-9.
18. Han, D.; Hao, S.; Tao, C.; Zhao, Q.; Wei, Y.; Song, Z.; Li, B. Comparison of once daily radiotherapy to 60 Gy and twice daily radiotherapy to 45 Gy for limited stage small-cell lung cancer. *Thorac Cancer* **2015**, *6*, 643-648, doi:10.1111/1759-7714.12262.
19. Tan, Y.; Zhu, Q. Curative effect of hyperfractionated accelerated radiotherapy combined with EP chemotherapy regimen on limited-stage small cell lung cancer. *J buon* **2021**, *26*, 837-843.
20. Tomita, N.; Kodaira, T.; Hida, T.; Tachibana, H.; Nakamura, T.; Nakahara, R.; Inokuchi, H. The impact of radiation dose and fractionation on outcomes for limited-stage small-cell lung cancer. *Int J Radiat Oncol Biol Phys* **2010**, *76*, 1121-1126, doi:10.1016/j.ijrobp.2009.03.069.
21. Socha, J.; Guzowska, A.; Tyc-Szczepaniak, D.; Wierzchowski, M.; Sprawka, A.; Szczesna, A.; Kepka, L. Accelerated hypofractionated thoracic radiotherapy in limited disease small cell lung cancer : comparison with the results of conventionally fractionated radiotherapy. *J BUON* **2015**, *20*, 146-157.
22. Zayed, S.; Chen, H.; Ali, E.; Rodrigues, G.B.; Warner, A.; Palma, D.A.; Louie, A.V. Is There a Role for Hypofractionated Thoracic Radiation Therapy in Limited-Stage Small Cell Lung Cancer? A Propensity Score Matched Analysis. *Int J Radiat Oncol Biol Phys* **2020**, *108*, 575-586, doi:10.1016/j.ijrobp.2020.06.008.
23. Zhang, J.; Fan, M.; Liu, D.; Zhao, K.-L.; Wu, K.-L.; Zhao, W.-X.; Zhu, Z.-F.; Fu, X.-L. Hypo- or conventionally fractionated radiotherapy combined with chemotherapy in patients with limited stage small cell lung cancer. *Radiat Oncol* **2017**, *12*, 51, doi:10.1186/s13014-017-0788-x.
24. Gregor, A.; Drings, P.; Burghouts, J.; Postmus, P.E.; Morgan, D.; Sahmoud, T.; Kirkpatrick, A.; Dalesio, O.; Giaccone, G. Randomized trial of alternating versus sequential radiotherapy/chemotherapy in limited-disease patients with small-cell lung cancer: a European Organization for Research and Treatment of Cancer Lung Cancer Cooperative Group Study. *J Clin Oncol* **1997**, *15*, 2840-2849, doi:10.1200/jco.1997.15.8.2840.
25. Jett, J.R.; Everson, L.; Therneau, T.M.; Krook, J.E.; Dalton, R.J.; Marschke, R.F., Jr.; Veeder, M.H.; Brunk, S.F.; Mailliard, J.A.; Twito, D.I., et al. Treatment of limited-stage small-cell lung cancer with cyclophosphamide, doxorubicin, and vincristine with or without etoposide: a randomized trial of the North Central Cancer Treatment Group. *J Clin Oncol* **1990**, *8*, 33-38, doi:10.1200/jco.1990.8.1.33.
26. Murray, N.; Coy, P.; Pater, J.L.; Hodson, I.; Arnold, A.; Zee, B.C.; Payne, D.; Kostashuk, E.C.; Evans, W.K.; Dixon, P., et al. Importance of timing for thoracic irradiation in the combined modality treatment of limited-stage small-cell lung cancer. The National Cancer Institute of Canada Clinical Trials Group. *J Clin Oncol* **1993**, *11*, 336-344, doi:10.1200/jco.1993.11.2.336.
27. Sculier, J.P.; Lafitte, J.J.; Efremidis, A.; Florin, M.C.; Lecomte, J.; Berchier, M.C.; Richez, M.; Berghmans, T.; Scherpereel, A.; Meert, A.P., et al. A phase III randomised study of concomitant induction radiochemotherapy testing two modalities of radiosensitisation by cisplatin (standard versus daily) for limited small-cell lung cancer. *Ann Oncol* **2008**, *19*, 1691-1697, doi:10.1093/annonc/mdn354.
28. Xia, B.; Hong, L.Z.; Cai, X.W.; Zhu, Z.F.; Liu, Q.; Zhao, K.L.; Fan, M.; Mao, J.F.; Yang, H.J.; Wu, K.L., et al. Phase 2 study of accelerated hypofractionated thoracic radiation therapy and concurrent chemotherapy in patients with limited-stage small-cell lung cancer. *Int J Radiat Oncol Biol Phys* **2015**, *91*, 517-523, doi:10.1016/j.ijrobp.2014.09.042.
29. Baas, P.; Belderbos, J.S.; Senan, S.; Kwa, H.B.; van Bochove, A.; van Tinteren, H.; Burgers, J.A.; van Meerbeeck, J.P. Concurrent chemotherapy (carboplatin, paclitaxel, etoposide) and involved-

field radiotherapy in limited stage small cell lung cancer: a Dutch multicenter phase II study. *Br J Cancer* **2006**, *94*, 625-630, doi:10.1038/sj.bjc.6602979.
30. Bogart, J.A.; Herndon, J.E., 2nd; Lyss, A.P.; Watson, D.; Miller, A.A.; Lee, M.E.; Turrisi, A.T.; Green, M.R. 70 Gy thoracic radiotherapy is feasible concurrent with chemotherapy for limited-stage small-cell lung cancer: analysis of Cancer and Leukemia Group B study 39808. *Int J Radiat Oncol Biol Phys* **2004**, *59*, 460-468, doi:10.1016/j.ijrobp.2003.10.021.
31. Bunn, P.A., Jr.; Crowley, J.; Kelly, K.; Hazuka, M.B.; Beasley, K.; Upchurch, C.; Livingston, R.; Weiss, G.R.; Hicks, W.J.; Gandara, D.R., et al. Chemoradiotherapy with or without granulocyte-macrophage colony-stimulating factor in the treatment of limited-stage small-cell lung cancer: a prospective phase III randomized study of the Southwest Oncology Group. *J Clin Oncol* **1995**, *13*, 1632-1641, doi:10.1200/jco.1995.13.7.1632.
32. Elias, A.; Ibrahim, J.; Skarin, A.T.; Wheeler, C.; McCauley, M.; Ayash, L.; Richardson, P.; Schnipper, L.; Antman, K.H.; Frei, E., 3rd. Dose-intensive therapy for limited-stage small-cell lung cancer: long-term outcome. *J Clin Oncol* **1999**, *17*, 1175, doi:10.1200/jco.1999.17.4.1175.
33. Hanna, N.; Ansari, R.; Fisher, W.; Shen, J.; Jung, S.H.; Sandler, A. Etoposide, ifosfamide and cisplatin (VIP) plus concurrent radiation therapy for previously untreated limited small cell lung cancer (SCLC): a Hoosier Oncology Group (HOG) phase II study. *Lung Cancer* **2002**, *35*, 293-297, doi:10.1016/s0169-5002(01)00429-9.
34. Kakolyris, S.; Agelidou, A.; Androulakis, N.; Tsaroucha, E.; Kouroussis, C.; Agelidou, M.; Karvounis, N.; Veslemes, M.; Christophylakis, C.; Argyraki, A., et al. Cisplatin plus etoposide chemotherapy followed by thoracic irradiation and paclitaxel plus cisplatin consolidation therapy for patients with limited stage small cell lung carcinoma. *Lung Cancer* **2006**, *53*, 59-65, doi:10.1016/j.lungcan.2006.03.011.
35. Kelley, M.J.; Bogart, J.A.; Hodgson, L.D.; Ansari, R.H.; Atkins, J.N.; Pang, H.; Green, M.R.; Vokes, E.E. Phase II study of induction cisplatin and irinotecan followed by concurrent carboplatin, etoposide, and thoracic radiotherapy for limited-stage small-cell lung cancer, CALGB 30206. *J Thorac Oncol* **2013**, *8*, 102-108, doi:10.1097/JTO.0b013e31827628e1.
36. Le, Q.T.; Moon, J.; Redman, M.; Williamson, S.K.; Lara, P.N., Jr.; Goldberg, Z.; Gaspar, L.E.; Crowley, J.J.; Moore, D.F., Jr.; Gandara, D.R. Phase II study of tirapazamine, cisplatin, and etoposide and concurrent thoracic radiotherapy for limited-stage small-cell lung cancer: SWOG 0222. *J Clin Oncol* **2009**, *27*, 3014-3019, doi:10.1200/jco.2008.21.3868.
37. Maurer, L.H.; Herndon, J.E., 2nd; Hollis, D.R.; Aisner, J.; Carey, R.W.; Skarin, A.T.; Perry, M.C.; Eaton, W.L.; Zacharski, L.L.; Hammond, S., et al. Randomized trial of chemotherapy and radiation therapy with or without warfarin for limited-stage small-cell lung cancer: a Cancer and Leukemia Group B study. *J Clin Oncol* **1997**, *15*, 3378-3387, doi:10.1200/jco.1997.15.11.3378.
38. McClay, E.F.; Bogart, J.; Herndon, J.E., 2nd; Watson, D.; Evans, L.; Seagren, S.L.; Green, M.R. A phase III trial evaluating the combination of cisplatin, etoposide, and radiation therapy with or without tamoxifen in patients with limited-stage small cell lung cancer: Cancer and Leukemia Group B Study (9235). *Am J Clin Oncol* **2005**, *28*, 81-90, doi:10.1097/01.coc.0000139940.52625.d0.
39. Miller, A.A.; Wang, X.F.; Bogart, J.A.; Hodgson, L.D.; Rocha Lima, C.M.; Radford, J.E.; Vokes, E.E.; Green, M.R. Phase II trial of paclitaxel-topotecan-etoposide followed by consolidation chemoradiotherapy for limited-stage small cell lung cancer: CALGB 30002. *J Thorac Oncol* **2007**, *2*, 645-651, doi:10.1097/JTO.0b013e318074bbf5.
40. Sohn, J.H.; Moon, Y.W.; Lee, C.G.; Kim, G.E.; Chung, K.Y.; Chang, J.; Kim, S.K.; Kim, Y.S.; Choi, B.W.; Choi, H.J., et al. Phase II trial of irinotecan and cisplatin with early concurrent radiotherapy in limited-disease small-cell lung cancer. *Cancer* **2007**, *109*, 1845-1950, doi:10.1002/cncr.22621.
41. Sun, J.M.; Ahn, Y.C.; Choi, E.K.; Ahn, M.J.; Ahn, J.S.; Lee, S.H.; Lee, D.H.; Pyo, H.; Song, S.Y.; Jung, S.H., et al. Phase III trial of concurrent thoracic radiotherapy with either first- or third-cycle chemotherapy for limited-disease small-cell lung cancer. *Ann Oncol* **2013**, *24*, 2088-2092, doi:10.1093/annonc/mdt140.

42. Wahba, H.A.; Halim, A.A.; El-Hadaad, H.A. Irinotecan plus cisplatin chemotherapy followed by concurrent thoracic irradiation with low-dose weekly cisplatin for limited-disease small-cell lung cancer. *Med Oncol* **2012**, *29*, 199-204, doi:10.1007/s12032-010-9772-0.
43. Xenidis, N.; Kotsakis, A.; Kalykaki, A.; Christophyllakis, C.; Giassas, S.; Kentepozidis, N.; Polyzos, A.; Chelis, L.; Vardakis, N.; Vamvakas, L., et al. Etoposide plus cisplatin followed by concurrent chemo-radiotherapy and irinotecan plus cisplatin for patients with limited-stage small cell lung cancer: A multicenter phase II study. *Lung Cancer* **2010**, *68*, 450-454, doi:10.1016/j.lungcan.2009.08.012.
44. Chen, G.Y.; Jiang, G.L.; Wang, L.J.; Qian, H.; Fu, X.L.; Yang, H.; Wu, K.L.; Zhao, S. Cisplatin/etoposide chemotherapy combined with twice daily thoracic radiotherapy for limited small-cell lung cancer: a clinical phase II trial. *Int J Radiat Oncol Biol Phys* **2005**, *61*, 70-75, doi:10.1016/j.ijrobp.2004.04.058.
45. Ettinger, D.S.; Berkey, B.A.; Abrams, R.A.; Fontanesi, J.; Machtay, M.; Duncan, P.J.; Curran, W.J., Jr.; Movsas, B.; Byhardt, R.W. Study of paclitaxel, etoposide, and cisplatin chemotherapy combined with twice-daily thoracic radiotherapy for patients with limited-stage small-cell lung cancer: a Radiation Therapy Oncology Group 9609 phase II study. *J Clin Oncol* **2005**, *23*, 4991-4998, doi:10.1200/jco.2005.00.414.
46. Grønberg, B.H.; Killingberg, K.T.; Fløtten, Ø.; Brustugun, O.T.; Hornslien, K.; Madebo, T.; Langer, S.W.; Schytte, T.; Nyman, J.; Risum, S., et al. High-dose versus standard-dose twice-daily thoracic radiotherapy for patients with limited stage small-cell lung cancer: an open-label, randomised, phase 2 trial. *Lancet Oncol* **2021**, *22*, 321-331, doi:10.1016/s1470-2045(20)30742-7.
47. Han, J.Y.; Lee, D.H.; Song, J.E.; Lee, S.Y.; Kim, H.Y.; Kim, H.T.; Lee, J.S. Randomized phase 2 study of irinotecan plus cisplatin versus gemcitabine plus vinorelbine as first-line chemotherapy with second-line crossover in patients with advanced nonsmall cell lung cancer. *Cancer* **2008**, *113*, 388-395, doi:10.1002/cncr.23582.
48. Han, J.Y.; Cho, K.H.; Lee, D.H.; Kim, H.Y.; Kim, E.A.; Lee, S.Y.; Lee, J.S. Phase II study of irinotecan plus cisplatin induction followed by concurrent twice-daily thoracic irradiation with etoposide plus cisplatin chemotherapy for limited-disease small-cell lung cancer. *J Clin Oncol* **2005**, *23*, 3488-3494, doi:10.1200/jco.2005.01.082.
49. Hu, X.; Bao, Y.; Xu, Y.J.; Zhu, H.N.; Liu, J.S.; Zhang, L.; Guo, Y.; Jin, Y.; Wang, J.; Ma, H.L., et al. Final report of a prospective randomized study on thoracic radiotherapy target volume for limited-stage small cell lung cancer with radiation dosimetric analyses. *Cancer* **2020**, *126*, 840-849, doi:10.1002/cncr.32586.
50. Hügli, A.; Moro, D.; Mermillod, B.; Bolla, M.; Alberto, P.; Bonnefoi, H.; Miralbell, R. Phase II trial of up-front accelerated thoracic radiotherapy combined with chemotherapy and optional up-front prophylactic cranial irradiation in limited small-cell lung cancer. Groupe d'Oncologie Thoracique des Régions Alpines. *J Clin Oncol* **2000**, *18*, 1662-1667, doi:10.1200/jco.2000.18.8.1662.
51. Jeremic, B.; Shibamoto, Y.; Acimovic, L.; Milisavljevic, S. Initial versus delayed accelerated hyperfractionated radiation therapy and concurrent chemotherapy in limited small-cell lung cancer: a randomized study. *J Clin Oncol* **1997**, *15*, 893-900, doi:10.1200/jco.1997.15.3.893.
52. Johnson, B.E.; Bridges, J.D.; Sobczeck, M.; Gray, J.; Linnoila, R.I.; Gazdar, A.F.; Hankins, L.; Steinberg, S.M.; Edison, M.; Frame, J.N., et al. Patients with limited-stage small-cell lung cancer treated with concurrent twice-daily chest radiotherapy and etoposide/cisplatin followed by cyclophosphamide, doxorubicin, and vincristine. *J Clin Oncol* **1996**, *14*, 806-813, doi:10.1200/jco.1996.14.3.806.
53. Kubota, K.; Hida, T.; Ishikura, S.; Mizusawa, J.; Nishio, M.; Kawahara, M.; Yokoyama, A.; Imamura, F.; Takeda, K.; Negoro, S., et al. Etoposide and cisplatin versus irinotecan and cisplatin in patients with limited-stage small-cell lung cancer treated with etoposide and cisplatin plus concurrent accelerated hyperfractionated thoracic radiotherapy (JCOG0202): a randomised phase 3 study. *Lancet Oncol* **2014**, *15*, 106-113, doi:10.1016/s1470-2045(13)70511-4.
54. Mennecier, B.; Jacoulet, P.; Dubiez, A.; Westeel, V.; Bosset, J.F.; Magnin, V.; Depierre, A. Concurrent cisplatin/etoposide chemotherapy plus twice daily thoracic radiotherapy in limited

stage small cell lung cancer: a phase II study. *Lung Cancer* **2000**, *27*, 137-143, doi:10.1016/s0169-5002(99)00103-8.

55. Saito, H.; Takada, Y.; Ichinose, Y.; Eguchi, K.; Kudoh, S.; Matsui, K.; Nakagawa, K.; Takada, M.; Negoro, S.; Tamura, K., et al. Phase II study of etoposide and cisplatin with concurrent twice-daily thoracic radiotherapy followed by irinotecan and cisplatin in patients with limited-disease small-cell lung cancer: West Japan Thoracic Oncology Group 9902. *J Clin Oncol* **2006**, *24*, 5247-5252, doi:10.1200/jco.2006.07.1605.
56. Schild, S.E.; Bonner, J.A.; Hillman, S.; Kozelsky, T.F.; Vigliotti, A.P.; Marks, R.S.; Graham, D.L.; Soori, G.S.; Kugler, J.W.; Tenglin, R.C., et al. Results of a phase II study of high-dose thoracic radiation therapy with concurrent cisplatin and etoposide in limited-stage small-cell lung cancer (NCCTG 95-20-53). *J Clin Oncol* **2007**, *25*, 3124-3129, doi:10.1200/jco.2006.09.9606.
57. Skarlos, D.V.; Samantas, E.; Briassoulis, E.; Panoussaki, E.; Pavlidis, N.; Kalofonos, H.P.; Kardamakis, D.; Tsiakopoulos, E.; Kosmidis, P.; Tsavdaridis, D., et al. Randomized comparison of early versus late hyperfractionated thoracic irradiation concurrently with chemotherapy in limited disease small-cell lung cancer: a randomized phase II study of the Hellenic Cooperative Oncology Group (HeCOG). *Ann Oncol* **2001**, *12*, 1231-1238, doi:10.1023/a:1012295131640.
58. Sorensen, M.; Lassen, U.; Palshof, T.; Jensen, B.B.; Johansen, J.; Jensen, P.B.; Langer, S.W. Topotecan and cisplatin in combination with concurrent twice-daily chemoradiation in limited disease small cell lung cancer-a Danish Oncological Lung Cancer Group (DOLG) phase II trial. *Lung Cancer* **2008**, *60*, 252-258, doi:10.1016/j.lungcan.2007.09.022.
59. Takada, M.; Fukuoka, M.; Kawahara, M.; Sugiura, T.; Yokoyama, A.; Yokota, S.; Nishiwaki, Y.; Watanabe, K.; Noda, K.; Tamura, T., et al. Phase III study of concurrent versus sequential thoracic radiotherapy in combination with cisplatin and etoposide for limited-stage small-cell lung cancer: results of the Japan Clinical Oncology Group Study 9104. *J Clin Oncol* **2002**, *20*, 3054-3060, doi:10.1200/jco.2002.12.071.
60. van Loon, J.; De Ruysscher, D.; Wanders, R.; Boersma, L.; Simons, J.; Oellers, M.; Dingemans, A.M.; Hochstenbag, M.; Bootsma, G.; Geraedts, W., et al. Selective nodal irradiation on basis of (18)FDG-PET scans in limited-disease small-cell lung cancer: a prospective study. *Int J Radiat Oncol Biol Phys* **2010**, *77*, 329-336, doi:10.1016/j.ijrobp.2009.04.075.
